# Reduced binding of apoE4 to complement factor H promotes amyloid‐β oligomerization and neuroinflammation

**DOI:** 10.15252/embr.202256467

**Published:** 2023-05-08

**Authors:** Larisa Chernyaeva, Giorgio Ratti, Laura Teirilä, Satoshi Fudo, Uni Rankka, Anssi Pelkonen, Paula Korhonen, Katarzyna Leskinen, Salla Keskitalo, Kari Salokas, Christina Gkolfinopoulou, Katrina E Crompton, Matti Javanainen, Lotta Happonen, Markku Varjosalo, Tarja Malm, Ville Leinonen, Angeliki Chroni, Päivi Saavalainen, Seppo Meri, Tommi Kajander, Adam JM Wollman, Eija Nissilä, Karita Haapasalo

**Affiliations:** ^1^ Department of Bacteriology and Immunology, Medicum and Human Microbiome Research Program, Faculty of Medicine University of Helsinki Helsinki Finland; ^2^ Humanitas University Milano Italy; ^3^ A.I. Virtanen Institute for Molecular Sciences, Faculty of Health Sciences University of Eastern Finland Kuopio Finland; ^4^ Institute of Biotechnology, HiLIFE Helsinki Institute of Life Science University of Helsinki Helsinki Finland; ^5^ Institute of Biosciences and Applications National Center for Scientific Research “Demokritos” Athens Greece; ^6^ Biosciences Institute, Newcastle University Newcastle‐Upon‐Tyne UK; ^7^ Division of Infection Medicine, Department of Clinical Sciences Lund University Lund Sweden; ^8^ Institute of Clinical Medicine – Neurosurgery University of Eastern Finland and Department of Neurosurgery, Kuopio University Hospital Kuopio Finland

**Keywords:** apoE, dementia, factor H, inflammation, neurodegeneration, Immunology, Molecular Biology of Disease, Neuroscience

## Abstract

The *APOE4* variant of apolipoprotein E (apoE) is the most prevalent genetic risk allele associated with late‐onset Alzheimer's disease (AD). ApoE interacts with complement regulator factor H (FH), but the role of this interaction in AD pathogenesis is unknown. Here we elucidate the mechanism by which isoform‐specific binding of apoE to FH alters Aβ1‐42‐mediated neurotoxicity and clearance. Flow cytometry and transcriptomic analysis reveal that apoE and FH reduce binding of Aβ1‐42 to complement receptor 3 (CR3) and subsequent phagocytosis by microglia which alters expression of genes involved in AD. Moreover, FH forms complement‐resistant oligomers with apoE/Aβ1‐42 complexes and the formation of these complexes is isoform specific with apoE2 and apoE3 showing higher affinity to FH than apoE4. These FH/apoE complexes reduce Aβ1‐42 oligomerization and toxicity, and colocalize with complement activator C1q deposited on Aβ plaques in the brain. These findings provide an important mechanistic insight into AD pathogenesis and explain how the strongest genetic risk factor for AD predisposes for neuroinflammation in the early stages of the disease pathology.

## Graphical abstract

The major complement regulator, factor H, binds Aβ‐associated apoE isoforms apoE2 and apoE3 but not the Alzheimer's disease‐associated isoform apoE4. This interaction reduces formation of toxic Aβ oligomers, inhibits apoE receptor binding, and regulates complement activation.

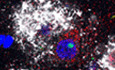

## Introduction

According to current understanding, accumulation of Aβ in the brain is caused by increased Aβ aggregation and impaired clearance of Aβ. The current findings on the association of complement markers with Aβ plaques and complement genes with risk of AD highlight the role of complement system in the disease pathogenesis (Lambert *et al*, 2009). Previous mouse models have demonstrated that the classical pathway (CP) components C1q and C4 contribute to microglia‐mediated loss of brain synapses in early AD (Hong *et al*, 2016). Aβ aggregates are known to activate the complement system in the brain, which triggers microglia activation. These activated microglial cells promote neuroinflammation and neurotoxicity and are involved in Aβ phagocytosis (Shen & Meri, 2003; Zhang *et al*, 2011).

Binding of C1q to apolipoprotein E (apoE) has been suggested to either initiate CP activation on surfaces or reduce the activation in solution, implicating that apoE, a major protein related to AD, is important in target recognition (Yin *et al*, 2019; Vogt *et al*, 2020). From the three allelic apoE isoforms (apoE2, apoE3, and apoE4), apoE4 is strongly associated with AD, while apoE2 is protective (Saunders *et al*, 1993). Idiopathic hydrocephalus (iNPH) is a neurodegenerative disease seen in aging population. The frontal cortex biopsy sample taken from these patients at shunt placement shows high frequency of amyloid β (Aβ) pathology and markers that correlate with early Alzheimer's disease (AD; Huang *et al*, 2021a). The apoE‐related AD risk also applies to patients with iNPH. ApoE is found abundantly in Aβ plaques. Several studies suggest that apoE and Aβ form complexes in fluid phase and the stability of this complex formation is isoform‐specific being higher with apoE3 compared to apoE4 (Bentley *et al*, 2002). There is also evidence that apoE influences Aβ cellular uptake through competitive binding to cell surface receptors (Verghese *et al*, 2013). ApoE is secreted as a lipid‐free form mainly by astrocytes and lipidated by ATP‐binding cassette transporters (Yamazaki *et al*, 2019). A small portion of apoE, however, is found associated with lipid‐poor proteins, from which especially apoE4 is susceptible to aggregation (Hatters *et al*, 2006). The current understanding is that apoE is involved in Aβ clearance, but the mechanisms underlying these effects are not fully known.

ApoE interacts with complement regulator factor H (FH) and reduces complement activation in plasma (Haapasalo *et al*, 2015). FH is found abundantly in plasma but is also expressed at lower levels in the brain (Huang *et al*, 2021a). It is essential for keeping complement activation under control and preventing complement attack toward host tissues (Walport, 2001). It acts as a cofactor to factor I in inactivation of C3b to iC3b, which is the ligand for complement receptor CR3, and thereby promotes non‐inflammatory clearance of unwanted material by macrophages (Gershov *et al*, 2000). FH is able to distinguish self‐cells from non‐self‐cells through recognition of host‐specific markers such as *N*‐acetylneuraminic acid, which is abundantly expressed on human cell surfaces. It also binds to self‐surfaces indirectly via C‐reactive protein (CRP; Haapasalo *et al*, 2015). When bound to surface‐associated CRP, it inhibits alternative pathway (AP) activation and C3b generation, which can act as an amplification loop through all the three complement activation pathways (Walport, 2001). FH consists of 20 homologous domains, which form an elongated modular structure. The surface recognition sites on FH are located in domains 5–7 and 19–20. Therefore, several known mutations and polymorphisms in FH and anti‐FH antibodies directed against these domains are associated with diseases (Jokiranta, 2017). Interestingly, there are some indications that a Y402H polymorphism in domain 7 of FH, which significantly increases the risk to age‐related macular degeneration (AMD), could also be associated with AD, although conflicting results on this association have been found in different genetic studies (Zetterberg *et al*, 2008; Le Fur *et al*, 2010).

This study was undertaken to provide insight into a potential mechanism by which interactions between FH and apoE work to reduce Aβ toxicity, plaque formation, and neuroinflammation, which have been implicated in the pathogenesis of AD. Here we show that interaction between apoE and FH, which is apoE isoform‐specific both *in vitro* and *in vivo*, alters their concerted action in reducing Aβ1‐42 oligomerization, interaction with the phagocytic receptor CR3 and Aβ1‐42 mediated inflammatory responses and cytotoxicity. The unique data obtained from biopsy tissue from living iNPH patients show colocalization of apoE and FH near Aβ plaques and complement activator C1q. Consistent with our *in vitro* findings, the increased expression of complement and AD‐associated proteins in the brain of at‐risk apoE4 allele carriers provides further evidence of the protective role of apoE‐FH interaction in Aβ‐induced neuroinflammation.

## Results

### Affinity between apoE and FH is apoE isoform‐specific both *in vivo* and *in vitro*

**Figure 1:**
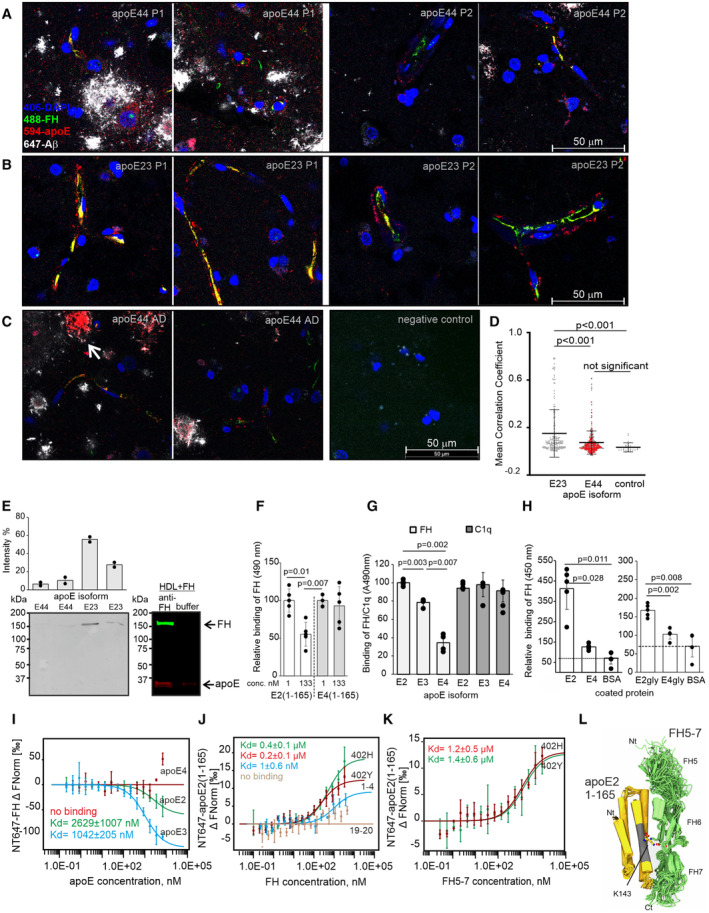
ApoE‐FH interaction is apoE isoform‐specific Immunofluorescence staining of apoE44 (*n* = 3) and apoE23 (*n* = 2) genotyped iNPH patient right frontal cortex biopsy samples (See Appendix Table S1). Two representative microscopy images from six separate images show:
Colocalization of (red) apoE44 and (green) FH in the presence of (white) Aβ plaques. Scale bar = 50 μm.Colocalization of (red) apoE23 and (green) FH around brain capillaries. The blue nuclei were detected using DAPI staining. See also Fig EV1 and Appendix Fig S1. Scale bar = 50 μm.Colocalization of (red) apoE44 and (green) FH in the presence of (white) Aβ plaques from a biopsy sample obtained from an iNPH patient diagnosed with Alzheimer's clinical syndrome (ACS). Colocalization of apoE on Aβ is shown with an arrow. The negative staining control is shown. See also Appendix Fig S2. Scale bar = 50 μm.ApoE‐FH colocalization analysis of all detected colocalized foci in six microscope images from the apoE44 (*n* = 3) and apoE23 (*n* = 2) genotyped iNPH patient biopsy samples. Six microscope images from Alzheimer's clinical syndrome (ACS) biopsy sample are included in the apoE44 dataset (red dots).(left above) Quantified intensities of two separate WBs showing presence of endogenous FH on HDL particles isolated from apoE44 (*n* = 2) and apoE23 (*n* = 2) genotyped iNPH patients (left below) in one representative membrane (See loading control and apoE staining in Fig EV1G). (right) Co‐immunoprecipitation of exogenously added FH with apoE23 from HDL particles.ApoE2(1–165) specific inhibition of FH binding to HDL‐associated apoE coated on ELISA plates.Binding of FH and C1q to different apoE isoforms.Binding of FH to non‐glycosylated (*E. coli* expression) and glycosylated (mammalian cell expression) human apoE2, and apoE4 and the negative control BSA.MST showing binding of NT647‐labeled full‐length FH to different apoE isoforms,NT647‐labeled apoE2(1–165) fragment to increasing concentrations of FH402Y and FH402H variants and FH domains 1–4 and 19–20.NT647‐labeled recombinant N‐terminal apoE2(1–165) to increasing concentrations of FH5‐7402Y and FH5‐7402H fragments.Structural model for apoE2(1–165)/FH5‐7 complex based on crosslinking mass spectrometry and molecular docking and molecular dynamics simulations. The best model ensemble after molecular dynamics simulation run of 250 ns is presented (apoE yellow, FH5‐7 green), apoE LDL receptor (LDLR) binding regions 135–150 shown in gray. The residues at the interface conserved in simulations including K143 of apoE are shown as sticks (Nt, N‐terminus; Ct, C‐terminus). See also Fig EV2A and B and Dataset EV1. Data information: Error bars indicate SD values calculated from pooled data (*n* = 1–3 samples/assay) obtained from repeated experiments (*n* = 3–4). Statistics was calculated using two‐tailed one‐way ANOVA supplemented with (D) Tukey's or (F–H) Dunnett's multiple comparison test using SPSS software or (I–K) MO. Affinity Analysis Software (NanoTemper) MST signal is shown as change in the normalized fluorescence (∆FNorm ‰) calculated by MO. Affinity Analysis Software. See also statistics in Appendix Table S2. Source data are available online for this figure.

**Figure EV1:**
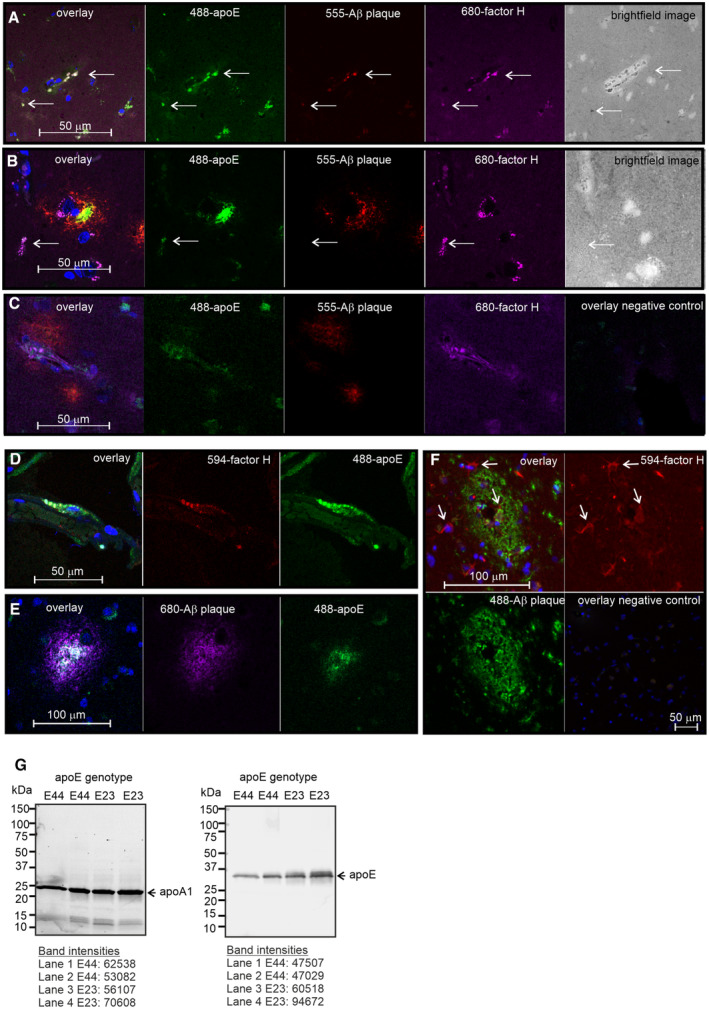
Localization of apoE and FH in brain Aβ plaques. Related to Fig 1 A, BImmunofluorescence staining of apoE33 and apoE34 geotyped iNPH patient biopsy samples showing (A) (arrow) colocalization of (green) apoE, (red) Aβ and (purple) FH in the brain, (B) colocalization of (green) apoE and (purple) FH around and away from brain capillaries surrounded by (red) Aβ plaques. The areas of colocalization are shown in the bright field images. Scale bar = 50 μm.CColocalization of (green) apoE, and (purple) FH around brain capillaries in the vicinity of (red) Aβ plaques. Scale bar = 50 μm.DColocalization of (green) apoE and (red) FH around cells covering capillaries in the brain, indicating protection of the blood–brain barrier (BBB) and endothelial cells from complement attack. The blue nuclei were detected using DAPI staining. Scale bar = 50 μm.EColocalization of (green) apoE in the (purple) Aβ plaque core. Scale bar = 50 μm.FA dense (green) amyloid plaque surrounded by cells showing localization of (red) FH around the cells and the Aβ core. (arrow) Localization of FH is shown.GRelated to Fig 1E. (left) Silver staining of a loading control showing the major apoA1 band of HDL and (right) apoE WB of HDL particles isolated from apoE genotyped iNPH patient plasma. HDL samples (5–20 μl based on protein concentration) were incubated with 1 × Bolt™ LDS Sample Buffer and 1 × Bolt™ Sample Reducing Agent (Thermo Fisher) for 10 min at 95°C and run on gel (Mini‐PROTEAN TGX Stain‐Free precast gels 4–20%, Bio‐Rad), 60 min, 120 V in TGS buffer (Bio‐Rad). (left) The gel was imaged with GelDoc system (Bio‐Rad). (right) WB was performed using 1:5,000 polyclonal antibody against purified human apoE (kind gift from Dr. Matti Jauhiainen) and IRDye® 680RD labeled anti‐Rabbit IgG (Cat#926‐68073, LI‐COR). (left) The major protein, ApoAI, at 25 kDa was found at similar levels in the samples. (right) The 37 kDa band of apoE is indicated.

**Figure EV2:**
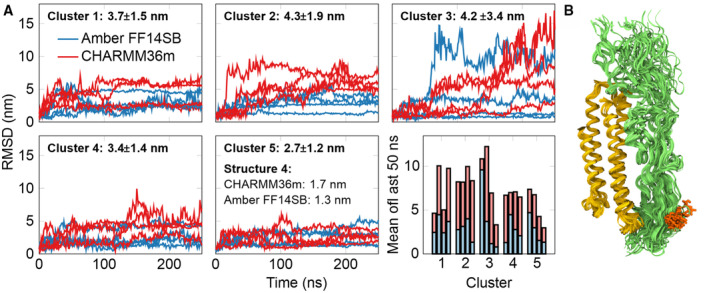
The trajectories of atomistic MD simulations for the different HADDOCK‐predicted structures. Related to Fig 1L The root mean square deviation (RMSD) is calculated for FH5‐7 after RMSD‐fitting apoE2 to its original conformation. Input data included five clusters each with four structures. MD Data for the two force fields are shown with different colors, and the numbers show the mean RMSD value and its standard deviation for each cluster. The bottom right panel shows the average values of the RMSD curves during the last 50 ns of simulation with the same coloring. Many initial structures were not stable, and the dimer dissociated rapidly during the simulation. Still, we observed that cluster 5 was on average the most stable, and no outliers were observed with either force field. Structure number 4 in this cluster had the smallest total RMSD value from simulations of the two force fields (values provided in the insert) and was thus chosen for further analysis on hydrogen bonds formed at the dimer interface.The best MD ensemble structure with residue 402 shown in red, pointing away from the protein interface between apoE2 (yellow) and FH5‐7 (green).

To gain insight into the interaction between apoE and FH, we studied binding of FH to apoE *in vivo* and whether these polymorphisms could affect this interaction. We used cortical brain biopsy samples obtained from apoE genotyped iNPH patients as a tool to understand the Aβ induced neuroinflammation *in vivo*, as 50% of iNPH patients develop lesions of Aβ plaques leading eventually to development of AD (Luikku *et al*, 2019; Appendix Table S1, patient data). The immunohistochemistry analysis revealed colocalization of apoE and FH associated with Aβ and on the surroundings of Aβ plaques and brain capillaries (Fig EV1A–F). Immunostaining and colocalization analysis showed significantly increased apoE‐FH colocalization in the presence of apoE23 when compared to apoE44 (Fig 1A–D). Interestingly, some but not all Aβ plaques contained an apoE core (Fig 1C). In the brain, the lipidation of secreted apoE varies from lipidated to non‐lipid or lipid‐poor form, whereas the profile and degree of apoE glycosylation varies depending on location. This variation did not, however, affect the apoE isoform specificity of apoE‐FH interaction as this finding was confirmed using immunoblotting of HDL particles isolated from plasma of apoE23 and apoE44 carrying iNPH patients (Figs 1E and EV1G). This interaction was apoE specific as the exogenously added FH co‐immunoprecipitated with apoE from the HDL. The observation that binding of FH to HDL was significantly inhibited by the N‐terminal fragment of apoE2(1–165) but not by apoE4(1–165; Fig 1F) showed that the C‐terminal lipid binding domain is not essential for apoE‐FH interaction. As expected, binding of immobilized recombinant apoE to FH *in vitro* was also apoE isoform specific as shown by significantly increased binding of apoE2 and significantly reduced binding of apoE4 to FH (Fig 1G). As published before, C1q bound all the apoE isoforms equally well (Yin *et al*, 2019). A similar difference between binding of FH to apoE2 and apoE4 was observed when both *E. coli* expressed and glycosylated mammalian cell expressed apoE recombinants were used (Fig 1H). Consistently, apoE2 and apoE3 bound FH with affinities of 2.6 and 1.0 μM in Microscale thermophoresis (MST), respectively, while apoE4 did not interact with full‐length FH (Fig 1I and Appendix Table S2, MST statistics). The N‐terminal fragment of apoE2 bound both the wild‐type FH402Y and the FH402H AMD risk variant equally well, while a lower affinity binding was observed to FH domains 1–4 (Fig 1J). ApoE2(1–165), however, did not bind FH domains 19–20 (FH19‐20). As expected, apoE2(1–165) bound the recombinant fragments of FH domains 5–7 (FH5‐7402Y and FH5‐7402H) equally well (Fig 1K). Together, these data suggest that binding of apoE to FH is apoE isoform‐specific both *in vivo* and *in vitro* with apoE4 displaying diminished FH binding capacity. The apoE binding site in FH is located within domains 5–7, similarly as published before (Haapasalo *et al*, 2015), but it may expand to domain 4 while the N‐terminal fragment of apoE is sufficient for apoE‐FH interaction. More specifically, a molecular model of the interaction complex of apoE with FH5‐7 based on crosslinking mass spectrometry and molecular docking combined with molecular dynamics simulations suggests the N‐terminal LDLR receptor binding region (136–150) of apoE2 is the major binding site for FH5‐7 (Figs 1L and EV2A and B; Dataset EV1; Chen *et al*, 2021). Also, the structural model suggests that the Y402 is outside the binding interface, in agreement with the observation that mutation Y402H does not affect the binding affinity.

### ApoE isoform‐specific binding of FH to apoE/Aβ1‐42 complexes reduces Aβ aggregation

**Figure 2:**
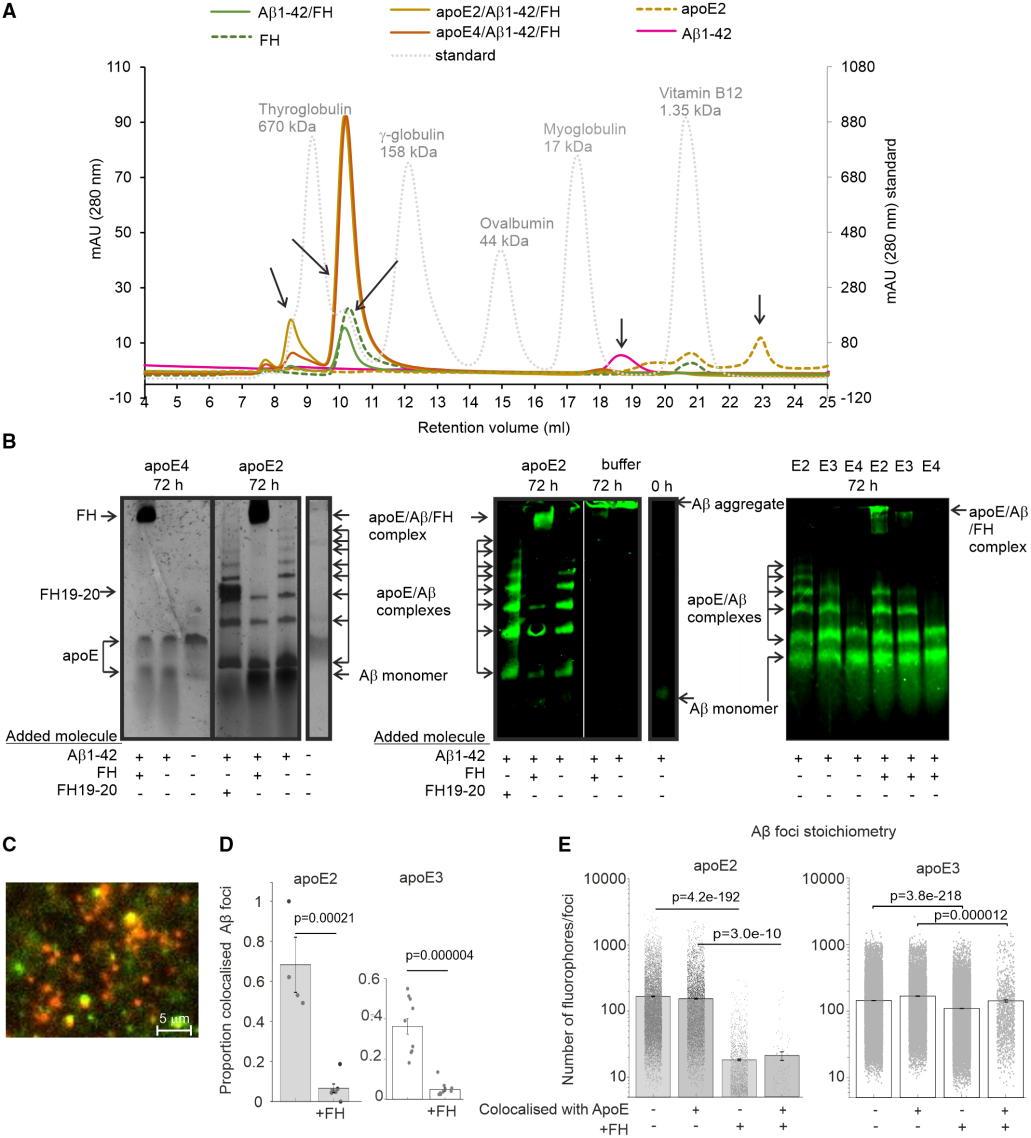
Binding of FH to apoE/Aβ1‐42 complexes reduces Aβ aggregation ApoE2, Aβ1‐42 and FH (apoE2/Aβ1‐42/FH), apoE4, Aβ1‐42 and FH (apoE4/Aβ1‐42/FH) and Aβ1‐42 and FH (Aβ1‐42/FH) were incubated for 72 h, and the complexes were isolated by size exclusion chromatography. The elution profiles of only FH, only Aβ1‐42 and only apoE2 and the size exclusion standard proteins are also shown.Different mixtures of apoE isoforms, FH, FH19‐20 and Aβ1‐42 were incubated for 72 h, and the presence of stable complexes was analyzed by running the sample in Tris‐glycine PAGE for (left and middle) 90 or (right) 60 min at 100 V in the presence of 1 x Bolt™ LDS Sample Buffer (Thermo Fisher). Gels were subjected to silver staining (left) or WB using anti‐Aβ antibody (middle and right). ApoE isoform and incubation time are shown on the top. The presence of each protein is indicated with arrows. Aβ1‐42 aggregate and the stable complexes formed between apoE, Aβ1‐42 and FH are shown near the top of the gel, while apoE2/Aβ1‐42 formed in the absence of FH are shown in several bands below apoE/Aβ1‐42/FH complexes. See also Fig EV3 for ELISA, anti‐FH/anti‐apoE dual fluorescence labeling, SDS–PAGE and native PAGE and Appendix Table S3 for band intensities.Single‐molecule TIRF micrograph of Hylite 488 labeled Aβ1‐42 and Alexa Fluor 568 C5 Maleimide labeled apoE2. Scale bar = 5 μm.Jitter plot of proportion of colocalized Aβ1‐42/apoE2 and Aβ1‐42/apoE3 over multiple fields of view in the presence and absence of FH. Statistics was calculated using Student's *t*‐test from multiple images (*n* = 12) in each sample. Error bars indicate SD values.Jitter plot of Aβ1‐42 foci stoichiometry in number of fluorophores colocalized or not with apoE2 and apoE3 and in the presence and absence of FH. Statistics of multiple images was calculated using two‐sided Student's *t*‐test. Statistics was calculated using Student's *t*‐test from all detected colocalized foci in multiple images (*n* = 12) in each sample. Error bars indicate SD values. See also Fig EV4. Source data are available online for this figure.

**Figure EV3:**
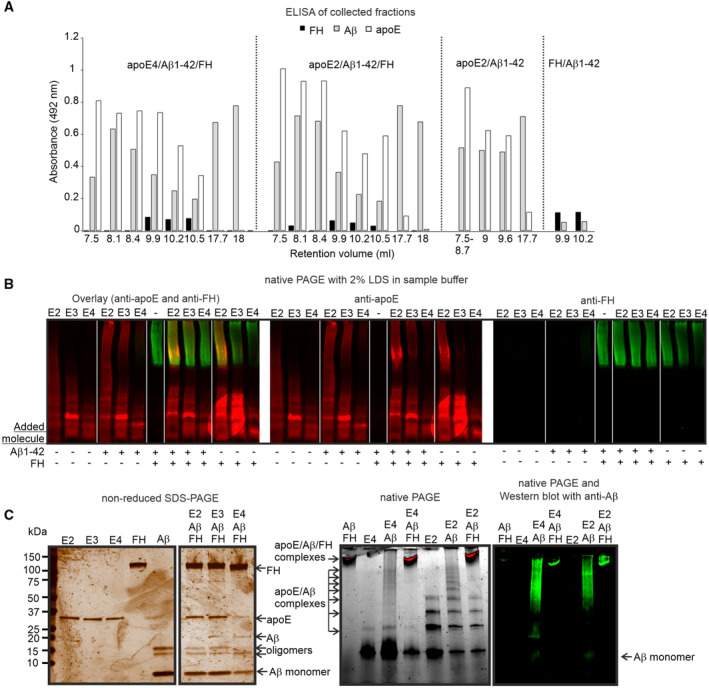
Analyzing FH, apoE and Aβ1‐42 complexes ELISA of collected fractions at different retention volumes (x‐axis) from size exclusion chromatography of apoE and 488‐Aβ1‐42‐incubated samples with and without FH shown in Fig 2A. The collected fractions were diluted 1:2 in sodium bicarbonate buffer, pH 9.6 and coated in 96‐well maxisorp plates. The wells were blocked with 3% fatty acid‐free BSA in PBS for 2 h and after one wash with PBS, incubated with 1:3,000 goat anti‐FH (Calbiochem), 1:3,000 rabbit polyclonal antibody against purified human apoE (from Dr. Matti Jauhiainen) or 1:1,000 rabbit anti‐Aβ (Invitrogen) antibodies in 0.3% BSA in PBS for 1 h at 37°C. The wells were washed three times with PBS and incubated with 1:10,000 diluted HRP‐conjugated anti‐goat or anti‐rabbit IgG antibodies (Jackson Immunoresearch) for 45 min at 37°C. After four washes with PBS, the OPD substrate was added in the wells, and the reaction was stopped after sufficient development of color with 3 M H_2_SO_4_ solution. The absorbance was measured at 492 nm.Related to Fig 2B. After 72 h incubation the samples were run on gels to conduct (above) native PAGE with Bolt LDS Sample Buffer (Thermo Fisher Scientific) and western blot of the samples using rabbit anti‐apoE and goat anti‐FH antibodies followed with dual labeling using IRDye® 680RD Donkey anti‐Rabbit IgG (Cat# 926‐68073, LI‐COR) and IRDye®800CW labeled anti‐Goat IgG (Cat# 926‐32213, LI‐COR) as described in Materials and Methods. Colocalization of FH with apoE2 and apoE3 (apoE2 > apoE3) but not with apoE4 can be detected in the presence and absence of Aβ1‐42.Aβ1‐42 and FH were all separated in (left) SDS–PAGE, while clear complexes were detected in (right) native PAGE. The presence of Aβ1‐42 in native PAGE was detected by WB using anti‐Aβ antibody. Loss of Aβ1‐42 monomer in the presence of FH and low intensity of Aβ1‐42 in complex with FH indicates minimal interaction between FH and Aβ1‐42 and aggregation of Aβ1‐42 in the absence of apoE. The intensity of apoE/Aβ/FH complexes indicates higher affinity of FH to apoE2/Aβ and then to apoE4/Aβ.

It has been suggested that native apoE forms stable apoE/Aβ complexes in an isoform‐specific manner and thereby affects Aβ clearance (Bilousova *et al*, 2019). In the fluorescent images of iNPH biopsy samples we detected colocalization of apoE and FH with small Aβ plaques but not with large Aβ plaques (Fig EV1A–C) suggesting that FH could affect the stability of apoE/Aβ complexes. To study apoE/Aβ complex formation in solution, we fractionated the proteins and protein complexes by size exclusion chromatography and analyzed the fractions by ELISA. We observed that both apoE2 and apoE4 eluted in association with Aβ1‐42 and FH, while FH without apoE eluted mainly as a single molecule (Figs 2A and EV3A). In SDS gels, the stability of apoE/Aβ was isoform‐specific ranging from apoE2 being most stable via apoE3 to apoE4 being less stable (Figs 2B and EV3B and C; Appendix Table S3). Consistent with the observed apoE isoform‐specific interactions between apoE and FH, FH formed stable complexes with apoE2 and apoE3 but not with apoE4 or Aβ1‐42 alone irrespective of the presence of Aβ1‐42 in the complex indicating that FH binds apoE on the complex.

**Figure EV4:**
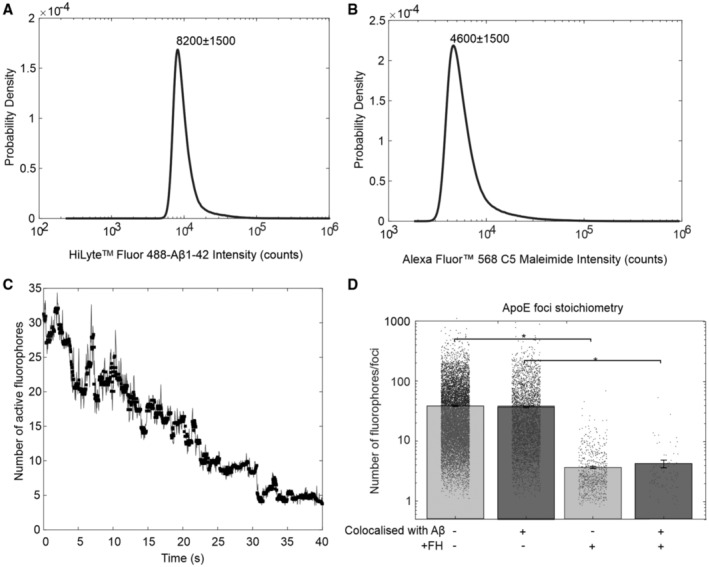
Single‐molecule characterization of apoE and Aβ1‐42 complexes. Related to Fig 2C–E A, BCharacteristic intensity distribution of single Hylite 488 and AlexaFluor 568 molecules.CStep‐wise photobleach trace of Aβ1‐42 oligomer foci. Raw intensity data plotted as line with Chung‐Kennedy filtered intensity overlaid as squares (Chung & Kennedy, 1991).DJitter plot of apoE2 foci stoichiometry in number of fluorophores colocalized or not with Aβ1‐42 and in the presence and absence of FH. Statistics (*P < 0.05) was calculated using Student's *t*‐test from each colocalized foci in multiple images (*n* = 12) in each sample. Error bars indicate SD values.

To verify the oligomeric state of Aβ1‐42 and stoichiometry of apoE2/Aβ1‐42 and apoE3/Aβ1‐42 complexes, we performed single molecule total internal reflection fluorescence (TIRF) imaging using fluorescently tagged Aβ1‐42 and apoE2 in the presence and absence of FH. Distinct foci of Aβ1‐42 and apoE were detected in the green and red channels respectively. The majority of foci were colocalized (Fig 2C and D) consistent with apoE2/Aβ1‐42 complexes except when FH was also included which significantly decreased the proportion of colocalized foci, consistent with FH disruption of the complex formation (Figs 2C–E and EV4A–D). Reduction in the number of fluorophores/foci that measures the stoichiometry of the apoE/Aβ1‐42 complex was more striking on apoE2/Aβ1‐42 complexes than apoE3/Aβ1‐42 complexes in the presence of FH. Molecular counting analysis of foci using step‐wise photobleaching (Fig EV4) revealed that both apoE2/Aβ1‐42 and apoE3/Aβ1‐42 from 10 to nearly 1,000 fluorescent molecules (Fig 2E), dropping to only a few 10s apoE2/Aβ1‐42 complexes in the presence of FH. These data indicate that FH binds to apoE on apoE/Aβ1‐42 complex in an isoform‐specific manner (apoE2 > apoE3 > apoE4). Binding of FH to apoE restricts the size of soluble Aβ aggregates and may thereby facilitate clearance of apoE/Aβ1‐42 complexes. This finding may explain why toxic Aβ aggregates are formed preferentially in the presence of apoE4 (Hatters *et al*, 2006).

### FH can be found in the vicinity of phagocytic cells and Aβ plaques *in vivo* and competes for binding with apoE and soluble Aβ1‐42 to CR3 *in vitro*

**Figure 3:**
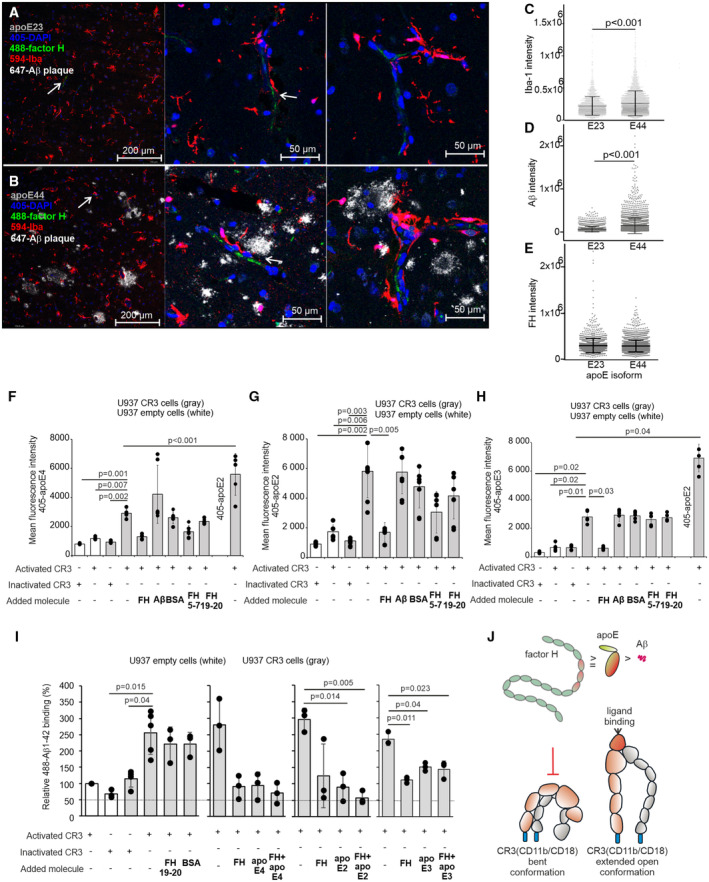
FH localizes near phagocytic cells and Aβ plaques and competes for binding with apoE and Aβ1‐42 to CR3 A, BImmunofluorescence staining of apoE44 (*n* = 2) and apoE23 (*n* = 2) genotyped iNPH patient right frontal cortex biopsy samples (See Appendix Table S1) showing localization of FH (green) with Iba‐1 stained cells (red) and clustering around cells and brain capillaries. Presence of (white) Aβ plaques in apoE44 samples and (arrow) FH in both samples are shown.C–E(C) Iba‐1, (D) Aβ, and (E) FH intensity analysis was calculated from all detected foci in six microscope images from the apoE44 (*n* = 2) and apoE23 (*n* = 2) genotyped iNPH patient biopsy samples.FFlow cytometric experiments showing binding of (left) 405‐labeled apoE4 and (right) 405‐labeled apoE2 to activated or inactivated CR3 on U937 empty and U937‐CR3 cells in the presence of FH, FH fragments 19–20 and 5–7, BSA and Aβ1‐42.GBinding of 405‐labeled apoE2 to activated or inactivated CR3 on U937 empty and U937‐CR3 cells in the presence of FH, FH fragments 19–20 and 5–7, BSA and Aβ1‐42.HBinding of (left) 405‐labeled apoE3 and (right) 405‐labeled apoE2 to activated or inactivated CR3 on U937 empty and U937‐CR3 cells in the presence of FH, FH fragments 19–20 and 5–7, BSA and Aβ1‐42.IBinding of 488‐labeled Aβ1‐42 to (left) activated or inactivated CR3 on U937 empty and U937‐CR3 cells in the presence of FH19‐20, BSA, with combinations of apoE4 and FH, apoE2 and FH or apoE3 and FH. The fluorescence intensities of 488‐Aβ1‐42 in each experiment were normalized against activated U937 empty cells sample. See also Appendix Fig S3A for gating and histograms.JSchematic presentation of apoE, Aβ and FH binding to CR3 (CD11b in brown color and CD18 in gray color) in the inactivated bent conformation (+EDTA) and the activated extended open conformation (+Mn^2+^). The competitive flow cytometric binding assay suggests that both apoE2 and FH have higher affinity to the ligand binding I‐domain in activated CR3 than Aβ1‐42, as they could outcompete Aβ1‐42 from CR3. FH domains 5–7 (red) bind both apoE and CD11b of CR3 (Nissilä *et al*, 2018). N‐terminal domain of apoE (red) interacts with FH and Aβ1‐42. Data information: Error bars indicate SD values calculated from pooled data (*n* = 1–3 samples/assay) obtained from repeated experiments (*n* = 3–4). Statistics was calculated using (C–E) Student's *t*‐test or (F–I) two‐tailed one‐way ANOVA supplemented with Dunnett's multiple comparison test using SPSS software. Source data are available online for this figure.

Increasing evidence suggests that extracellular accumulation of toxic Aβ aggregates is caused by imbalance between Aβ production and clearance. ApoE forms a complex with Aβ that is efficiently taken up by microglia (Yeh *et al*, 2016). Cellular uptake of Aβ is mediated through various cellular receptors such as the phagocytic receptor CR3 (Doens & Fernandez, 2014). Expression of CR3, however, has been shown to increase rather than reduce Aβ accumulation *in vivo* (Czirr *et al*, 2017). CR3 has an important role in mediating phagocytosis of iC3b‐opsonized particles but it also interacts with various ligands including Aβ and FH. Moreover, CR3 is also involved in reducing extracellular Aβ degradation and mediates neurotoxic effects (Zhang *et al*, 2011; Doens & Fernandez, 2014; Nissilä *et al*, 2018). Because of the suggested harmful role of Aβ‐CR3 interaction, we wanted to study whether FH in the presence or absence of apoE could selectively block binding of soluble Aβ to CR3. First, we examined whether FH, apoE and Aβ are located in the vicinity of phagocytic cells *in vivo* in the biopsy samples from apoE genotyped iNPH patients. We observed clusters of activated cells (microglia, or peripheral‐derived macrophages), as demonstrated by Iba‐1 staining, around but did not colocalize with FH coated surfaces (Fig 3A and B) and Aβ plaques (Fig 3B). The apoE44 samples had increased levels of both Aβ and Iba‐1 (Fig 3C–E). The quantity of FH, however, was the same between these samples (Fig 3E) although a significant difference between apoE‐FH colocalization was detected (Fig 1A–D). Aβ plaques frequently colocalized with apoE and contained an apoE protein core. FH did colocalize with apoE and Aβ plaques and was also observed between Aβ and apoE core as well as on the cells and surfaces surrounding the plaques (Fig EV1A–F).

We used genetically uniform U937 cells overexpressing CR3 to study the role of FH and apoE isoforms in Aβ binding and phagocytosis. The CR3 receptor consists of one α‐chain (CD11b) and one β‐chain (CD18) from which CD11b interacts with multiple ligands including fibrinogen, ICAM, FH, and the inactivated form of C3b, iC3b. The ligand binding site in CD11b is located in the N‐terminal αI‐domain that is exposed when the receptor is activated and adopt the extended open conformation (Lamers *et al*, 2021). In the *in vitro* flow cytometric analysis, all apoE isoforms bound to CR3, when the receptor was in active extended open conformation, suggesting that apoE interacts with the ligand‐binding I‐domain of CD11b (Fig 3F–H; Chen *et al*, 2010). The binding of apoE4 to CR3 was, however, significantly reduced when compared to apoE2. The full‐length FH but not the fragments of FH, Aβ1‐42 or BSA inhibited binding of apoE2 to CR3, indicating competitive binding between apoE2 and FH to CR3 and that apoE2‐FH complex (Fig 1) does not interact with the integrin. Aβ1‐42 also bound to the I‐domain of CD11b (Fig 3I, Appendix Fig S3A), but the affinity of Aβ1‐42 to CR3 was lower than the affinity of apoE2, apoE4 or FH, as all of these molecules inhibited Aβ1‐42 binding to CR3. Inhibition was most significant in the presence of both apoE2 and FH, indicating combined inhibitory effect of these molecules in Aβ‐CR3 interaction. All these data together indicate that apoE and Aβ share at least partially the same binding site on activated CR3 (Fig 3J) and that the affinity of apoE to CR3 is isoform‐specific (apoE2 > apoE4). The increased inhibitory function of the apoE2‐FH combination could be due to both FH/apoE2/Aβ complex formation in fluid phase (Fig 2A and B) and competitive binding between Aβ and apoE or Aβ and FH to CR3. Because binding of Aβ to CR3 may promote neurotoxic effects (Zhang *et al*, 2011), the observed antagonizing function of apoE2 and FH may have important anti‐inflammatory effects.

### FH associated with apoE/Aβ1‐42 complex increases complement and phagocytosis resistance

**Figure 4:**
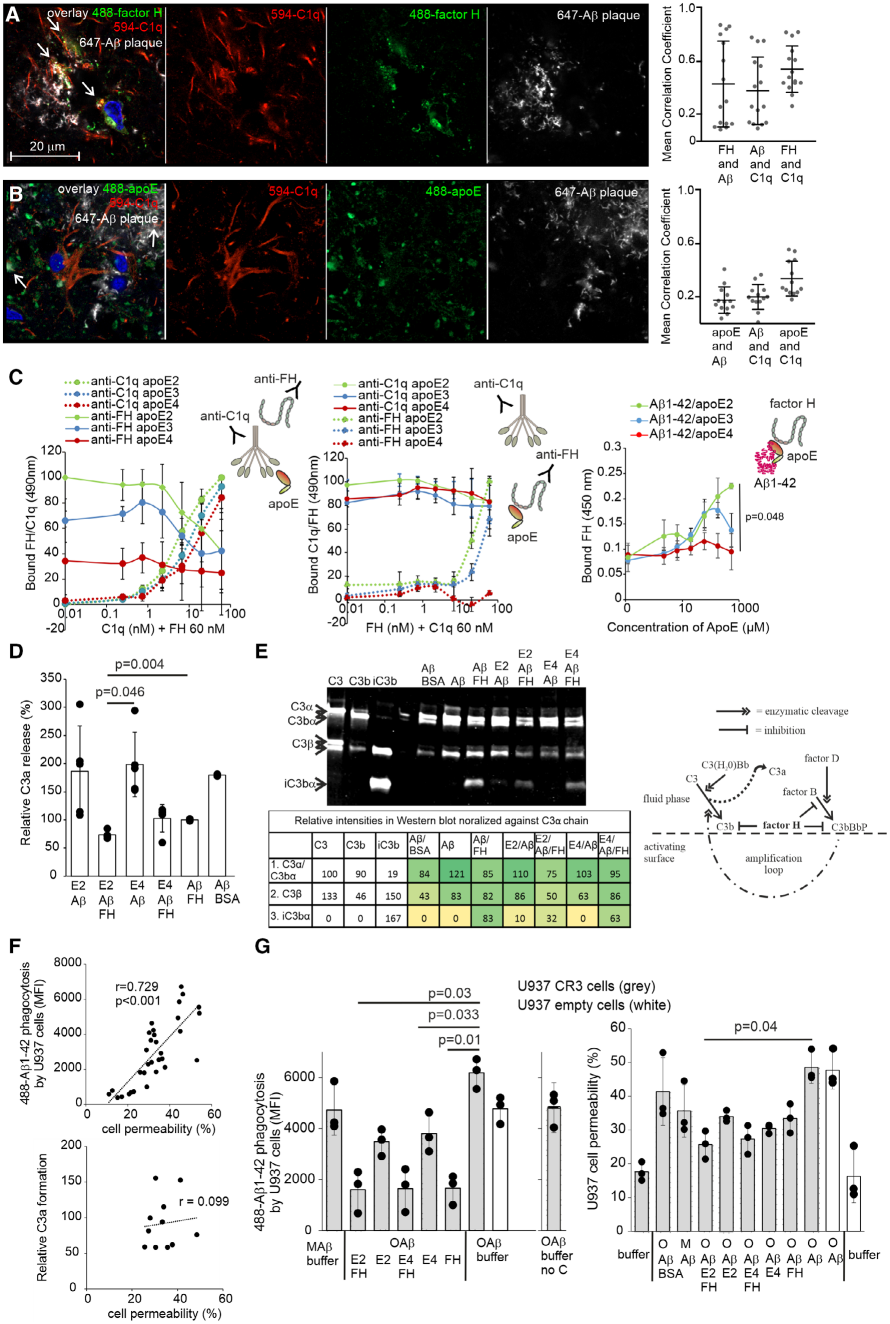
FH colocalizes with Aβ‐associated C1q *in vivo* and reduces complement activation, phagocytosis, and toxicity of apoE/Aβ1‐42 complexes Immunofluorescence staining of iNPH patient biopsy samples (apoE33; See Appendix Table S1, patient data) showing (arrow) colocalization of complement activator, (red) C1q, and complement regulator (green) FH.(arrow) Colocalization of (green) apoE and (red) C1q on (white) Aβ plaques. Colocalization analysis is calculated from colocalized foci in two separate microscope images.Simultaneous binding of C1q and FH to different apoE isoforms *in vitro*. (left) Increasing concentrations of C1q and constant concentration of FH detected by anti‐C1q and anti‐FH antibodies. (middle) Increasing concentrations of FH and constant concentration of C1q detected by anti‐FH and anti‐C1q antibodies. (right) Increasing concentrations of apoE with constant concentration of Aβ1‐42 incubated with constant concentration of FH detected with anti‐FH antibody.C3a formation in supernatant of C3, factor B (fB), factor D (fD) and factor I (fI) incubated beads coupled with apoE2/488‐Aβ1‐42, apoE4/488‐Aβ1‐42, apoE2/488‐Aβ1‐42/FH, apoE4/488‐Aβ1‐42/FH, 488‐Aβ1‐42/FH complexes. Beads with 488‐Aβ1‐42/BSA complexes show the level of C3a formation without inhibitor. Release of C3a is calculated as relative C3a increase % compared to the absorbance measured from 488‐Aβ1‐42/FH sample.(left) Cofactor activity of FH in the complexes coupled to the beads detected by the presence of the cleavage fragment of C3b α‐chain iC3bα. Table below shows quantification of the bands (See also Appendix Table S4 for band intensities). (right) Schematic presentation the AP and the amplification loop inhibited by FH at the level of C3b.Correlation between cell permeability and (above) phagocytosis or (below) C3a formation of different 488‐Aβ1‐42 containing protein complexes that were coupled in the beads and incubated with C3, fB, fD, and fI.(left) Phagocytosis of complement C3, fB, fD and fI (C) or only buffer (no C) incubated beads coupled with apoE2/488‐Aβ1‐42, apoE4/488‐Aβ1‐42, apoE2/488‐Aβ1‐42/FH, apoE4/488‐Aβ1‐42/FH, 488‐Aβ1‐42/FH and 488‐Aβ1‐42/BSA (oligomerized Aβ = OAβ) or with non‐oligomerized 488‐Aβ1‐42 (monomeric Aβ = MAβ). Also, samples without apoE or FH were included in the assay (buffer). (right) Cell permeability of C3, fB, fD and fI incubated beads coupled with apoE2/488‐Aβ1‐42, apoE4/488‐Aβ1‐42, apoE2/488‐Aβ1‐42/FH, apoE4/488‐Aβ1‐42/FH, 488‐Aβ1‐42/FH and 488‐Aβ1‐42/BSA complexes (OAβ) or with MAβ. Also, cells incubated with only buffer were included in the assay (buffer). Cell permeability is calculated by flow cytometry as % of DAPI stained cells in the cell population. The effect of CR3 expression in the phagocytosis was measured using PMA‐activated U937 cells with (U937 CR3 cells) or without (U937 empty cells) CR3 expression. Data information: Error bars indicate SD values. (C, D and G) Statistical significances were calculated using two‐tailed one‐way ANOVA supplemented with Dunnett's multiple comparison tests or (F) Pearson's correlation coefficient analysis using SPSS software from (C, G) four times repeated experiments or from (D) pooled data (two samples/assay) obtained from three times repeated experiments. Source data are available online for this figure.

Activation of the AP is initiated through spontaneous hydrolysis of C3 and therefore, without sufficient down‐regulation it can attack our own cells. The hydrolyzed C3, binds factor B, exposing factor B to cleavage by factor D, thus forming C3(H_2_O)Bb that cleaves fluid phase C3 to C3b and C3a. C3b forms deposits on the target surface and when inactivated to iC3b it acts as an opsonin to CR3. C3a is released in fluid phase, which induces inflammation through interaction with C3aR (Walport, 2001). This signaling has been suggested to promote vascular inflammation and blood–brain‐barrier (BBB) dysfunction (Bhatia *et al*, 2021). Aβ aggregates activate the complement system in the brain and thus, trigger the development of inflammation (Shen & Meri, 2003). Because we observed that FH forms more stable complexes with apoE2/Aβ1‐42 than with apoE4/Aβ1‐42 and reduces formation of large Aβ aggregates, we hypothesized that the interaction between apoE2 and FH could be a crucial mechanism to downregulate complement activation on Aβ. Consistent with the known immunoreactivity of Aβ, the Aβ plaques contained complement C1q *in vivo* (Fig 4A and B) as marker of CP activation and C3b deposition (Baik *et al*, 2016). Importantly, FH and C1q were colocalized on Aβ plaques (Fig 4A) indicating simultaneous rather than competitive binding of C1q and FH on Aβ plaques. This was verified by *in vitro* ELISAs where C1q showed some, but not significant, reduction in binding of FH to apoE2 and apoE3 (Fig 4C), while no inhibition of C1q binding by FH to any of the apoE isoforms was detected. As expected, binding of FH, but not C1q, to apoE was isoform‐specific (apoE2 > apoE3 > apoE4) providing increased competitive advantage of C1q over FH for binding to apoE4, which triggers complement activation. Importantly, binding of FH to Aβ coated wells was only observed in the presence of apoE2 and apoE3 but not in the presence of apoE4 or Aβ1‐42 alone. This was consistent with the size exclusion chromatography and PAGE experiments (Figs 2A and B, and EV3A and B) showing that FH does not bind Aβ1‐42 alone but binding of FH to Aβ/apoE is mediated via apoE2 or apoE3.

In the absence of regulation also the C1q initiated CP activation eventually leads to C3b formation and AP activation (Vogt *et al*, 2020). To further understand the complement inhibitory role of FH on apoE coated Aβ plaques, we next analyzed FH activity upon complement activation when bound to apoE2/Aβ1‐42 and apoE4/Aβ1‐42. The protein complexes were coupled into latex beads and their ability to activate and inactivate the first steps of AP was analyzed. As expected, all the apoE‐containing Aβ1‐42 complexes that were not associated with FH‐activated complement as detected by the release of C3a in the fluid phase (Fig 4D). However, the presence of FH reduced C3a levels in all samples but significantly in the supernatant of the FH/apoE2/Aβ1‐42 complex when compared to Aβ1‐42/FH. FH was functional on all the samples as shown by the presence of the cleavage fragment of the α‐chain of C3b, iC3bα, in the immunoblot (Fig 4E; Appendix Table S4). Importantly, a reduction of all C3b fragments was observed especially on FH/apoE2/Aβ1‐42 indicating inhibition of complement amplification and C3b formation. Together these results evidenced that FH is functional when associated with apoE/Aβ1‐42 complexes. The increased binding of FH to apoE2/Aβ1‐42 reduced formation of C3a and deposition of C3b and iC3b while reduced binding of FH to apoE4/Aβ1‐42 and formation of larger numbers of C3a, C3b, and iC3b indicated induced inflammation and increased CR3 mediated phagocytosis of these Aβ complexes (Haapasalo & Meri, 2019).

### FH inhibits phagocytosis of apoE/Aβ1‐42 complexes and thereby reduces Aβ1‐42‐mediated cell toxicity

According to current understanding, Aβ phagocytosis may play a less important role in Aβ clearance as the majority of extracellular Aβ is removed across the BBB (Zhao *et al*, 2015). This is also consistent with the finding that CSF Aβ levels are significantly reduced in AD patients when compared to healthy individuals (Palmqvist *et al*, 2014). To further understand the role of FH in clearance of apoE/Aβ complexes, we investigated whether iC3b and CR3 are involved in the uptake of complement challenged protein complexes using flow cytometry. Somewhat surprisingly, uptake of apoE/Aβ1‐42 correlated positively with cell permeability (Fig 4F). This was not caused by overactivation of complement as no correlation was observed between C3a formation and cell permeability. However, when the activated empty and CR3‐expressing U937 cells were challenged to apoE/Aβ1‐42, the presence of FH, even when challenged to complement, showed significant reduction in phagocytosis (Fig 4G). This observation suggests that formation of iC3b on these complexes has some but a less important effect in phagocytosis than expected and that exaggerated uptake of cytotoxic Aβ1‐42 is mediated through various receptors (Kam *et al*, 2013; Czirr *et al*, 2017). Interestingly, regulation of phagocytosis of Aβ1‐42 complexes by the concerted action of FH and apoE2 significantly reduced Aβ1‐42 cytotoxicity (Fig 4G).

### Inhibition of apoE/Aβ1‐42 phagocytosis by FH alters AD‐associated microglia cell responses *in vitro*

**Figure 5:**
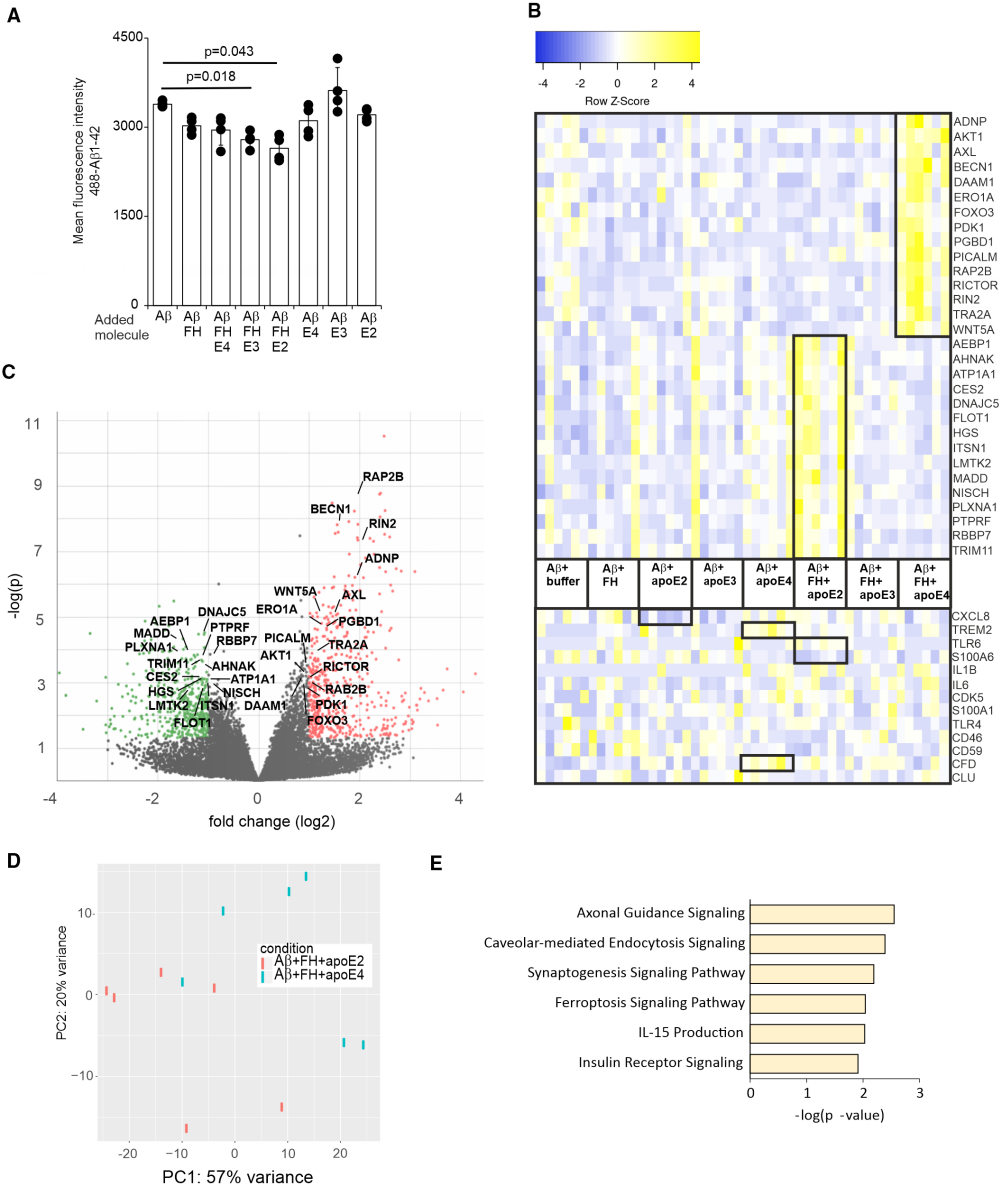
488‐Aβ1‐42 phagocytosis and microglial transcriptomics in the presence of FH and apoE isoforms 488‐Aβ1‐42 phagocytosis in the presence of FH and apoE isoform. Flow cytometry data are presented as mean fluorescence intensity of the cell population of the sample (See Appendix Fig S3B for gating and histograms).Heat map of the genes that were up‐ or downregulated between Aβ + apoE2 + FH and Aβ + apoE4 + FH or compared to Aβ + buffer (See statistics in Dataset EV2). The fold change is calculated between apoE2 + FH and apoE4 + FH. The trimmed mean of M values (TMM‐normalized) of mRNA transcription levels from the RNA‐seq data from two individual samples obtained from repeated experiments (*n* = 3) is presented with different combinations of FH, apoE and Aβ1‐42 proteins. Heat map was created by heatmapper (Babicki *et al*, 2016).Volcano plot of up‐ or downregulated genes between Aβ + apoE2 + FH and Aβ + apoE4 + FH.Principle Component Analysis (PCA) score plot showing the relationship between transcript expression profiles from FH + apoE2 and FH + apoE4 samples (See also Table 1 and Appendix Table S5).Top pathways identified by IPA analysis (Qiagen) within the differentially expressed genes between Aβ + apoE2 + FH and Aβ + apoE4 + FH. Data information: Statistical significances were calculated using two‐tailed one‐way ANOVA supplemented with Dunnett's multiple comparison tests using SPSS software. Error bars indicate SD values calculated from pooled data (*n* = 1–3 samples/assay) obtained from four times repeated experiments. Source data are available online for this figure.

The presence of Aβ is known to activate microglial cells. Therefore, we next wanted to understand how microglial cells respond to the presence of apoE and FH by challenging microglial SV40 cells with different apoE isoforms and Aβ1‐42 in the presence or absence of FH. As expected, Aβ1‐42 phagocytosis was significantly reduced in the presence of Aβ + FH + apoE2, and Aβ + FH + apoE3, but not in the presence of FH + apoE4 when compared to the cells incubated only with Aβ1‐42 (Fig 5A; Appendix Fig S3B). The live cell imaging analysis, however, showed that the uptake of Aβ1‐42 was not completely inhibited in the presence of Aβ + FH + apoE2 indicating that apoE and FH direct a controlled uptake and clearance of Aβ by microgial cells (Movies EV1 and EV2). The transcriptome analysis of the microglial cells revealed a number of genes that showed differential expression (DE) between cells exposed to different combinations of apoE isoforms and FH upon Aβ phagocytosis. Comparison between Aβ + apoE3 + FH vs. Aβ + apoE2 + FH revealed 18 DE genes, Aβ + apoE3 + FH vs. Aβ + apoE2 + FH 239 DE genes while the amount of DE genes was highest between Aβ + apoE4 + FH vs. Aβ + apoE2 + FH samples, 445 DE genes (Dataset EV2). When we compared DE genes of Aβ + apoE2 + FH and Aβ + apoE4 + FH samples to all samples we observed upregulation of genes (LMTK2, ATP1A1, FLOT1 and DNAJC5; Tiwari *et al*, 2015; Petrushanko *et al*, 2016; Abdullah *et al*, 2019; Bencze *et al*, 2019) that when downregulated could potentially promote AD pathogenesis (Fig 5B–D, Table 1, Appendix Table S5 and Dataset EV2). On the other hand, those genes upregulated in AD, such as AHNAK (Manavalan *et al*, 2013) and AEBP1 (Shijo *et al*, 2018), have different physiological roles and may participate in neurodegeneration. Five of the detected genes (DAAM1, PGBD1, PLXNA1, RBBP7, AKT1) also show similar changes in transcription levels and correlation with Aβ pathology in a recent mRNA transcriptomic data from iNPH biopsy samples (Huang *et al*, 2021a). Microglial cells incubated with FH and apoE4 showed upregulation of genes involved in Aβ processing and inflammation (Table 1). For example, PICALM (Moreau *et al*, 2014) is involved in autophagy and clearance of Aβ, BECN1 (Rocchi *et al*, 2017) aids autophagy and APP processing, AKT1 is important for ROS production (Ahmad *et al*, 2017), AXL has recently been linked to AD due to its direct role in promoting Aβ plaque development through microglial phagocytosis (Huang *et al*, 2021b), and RICTOR (Lee *et al*, 2017) is important for protecting the cells from Aβ‐induced toxicity. Differential expression of some key cellular and molecular mediators of neuroinflammation (Yang, 2019) could also be observed between cells incubated with only Aβ and Aβ + apoE2 (reduced CXCL8), Aβ and Aβ + apoE4 (increased TREM2 and CFD), Aβ and Aβ + apoE2 + FH (increased TLR6 and reduced S100A6). (Yang, 2019) From these neuroinflammatory markers, increased TREM2 expression has previously been shown to correlate with Aβ pathology in human biopsies (Huang *et al*, 2021a). Thus, the differential expression analysis guided identification of new and previously published specific markers linked to pathways (Fig 5E) that when disrupted or abnormally induced could be linked to the process of neuroinflammation and/or neurodegeneration.

**Table 1:** Differential expression of transcripts in microglial cells upon stimulation with different combinations of apoE, Aβ1‐42, and FH (Related to Fig 5B, See also Appendix Table S5).

Gene name (alternative name)	Gene name extended/encoded protein	Description	Cited in
Upregulation with FH and apoE2			
LMTK2	Lemur tyrosine kinase‐2	LMTK2 if reduced, is able to impair apoptosis, axonal transport and tau hyperphosphorylation, those are all involved in neurodegeneration.	Bencze *et al* (2019)
AHNAK (desmoyokin)	Neuroblast differentiation‐associated protein	AHNAK is fundamental in the process of myelination during development phase and in neuronal plasticity, degeneration and regeneration, while tau lesions are present differently in early and late matured oligodendrocytes in AD.	Manavalan *et al* (2013)
ATP1A1	ATPase Na^+^/K^+^ Transporting Subunit Alpha 1	ATP1A1 is less expressed in AD because Aβ1‐42 forms a complex with it, thus impairing both its hydrolytic and enzymatic function.	Petrushanko *et al* (2016)
FLOT1	Flotillin‐1	Flotillin 1 protein functions in vesicular trafficking and since it is less expressed in case of Aβ formation, it could possibly be used as an Aβ formation marker.	Abdullah *et al* (2019)
AEBP1	Adipocyte enhancer binding protein 1	AEBP1 is regularly expressed in neurons, but in case of pretangle formation in neurons and Aβ plaques, this protein is accumulated, thus suggesting an implication in AD.	Shijo *et al* (2018)
DNAJC5	Cysteine string protein alpha (CSPalpha)	CSPalpha has a fundamental role in AD, where it is initially inhibited, thus leading to synaptic degeneration.	Tiwari *et al* (2015)
Upregulation with FH and apoE4			
PICALM	Phosphatidylinositol binding clathrin assembly protein	PICALM modulates production, transport, and clearance of Aβ peptide, as well as regulating autophagy and clearance of tau.	Moreau *et al* (2014)
BECN1	Beclin 1	BECN1 can increase autophagy rate, thus decreasing Aβ deposition, preventing cognitive impairment and finally restoring survival rate in mice models for AD.	Rocchi *et al* (2017)
AKT1	Protein kinase B	Component of PI3K/AKT/mTOR pathway. AKT1 promote synaptic degeneration in AD if it is modified by ROS activity.	Ahmad *et al* (2017)
AXL	AXL receptor tyrosine kinase	Microglial phagocytosis driven by this receptor promotes plaque development.	Huang *et al* (2021b)
RICTOR (mTOR2)	RPTOR Independent Companion Of MTOR Complex 2	RICTOR avoids toxicity in neurons caused by Aβ formation and is also involved in formation of Aβ induced by insulin resistance.	Lee *et al* (2017)

### The presence of apoE4 isoform reveals increased complement activity, inflammation and upregulation of AD‐associated risk proteins *in vivo*

**Figure 6:**
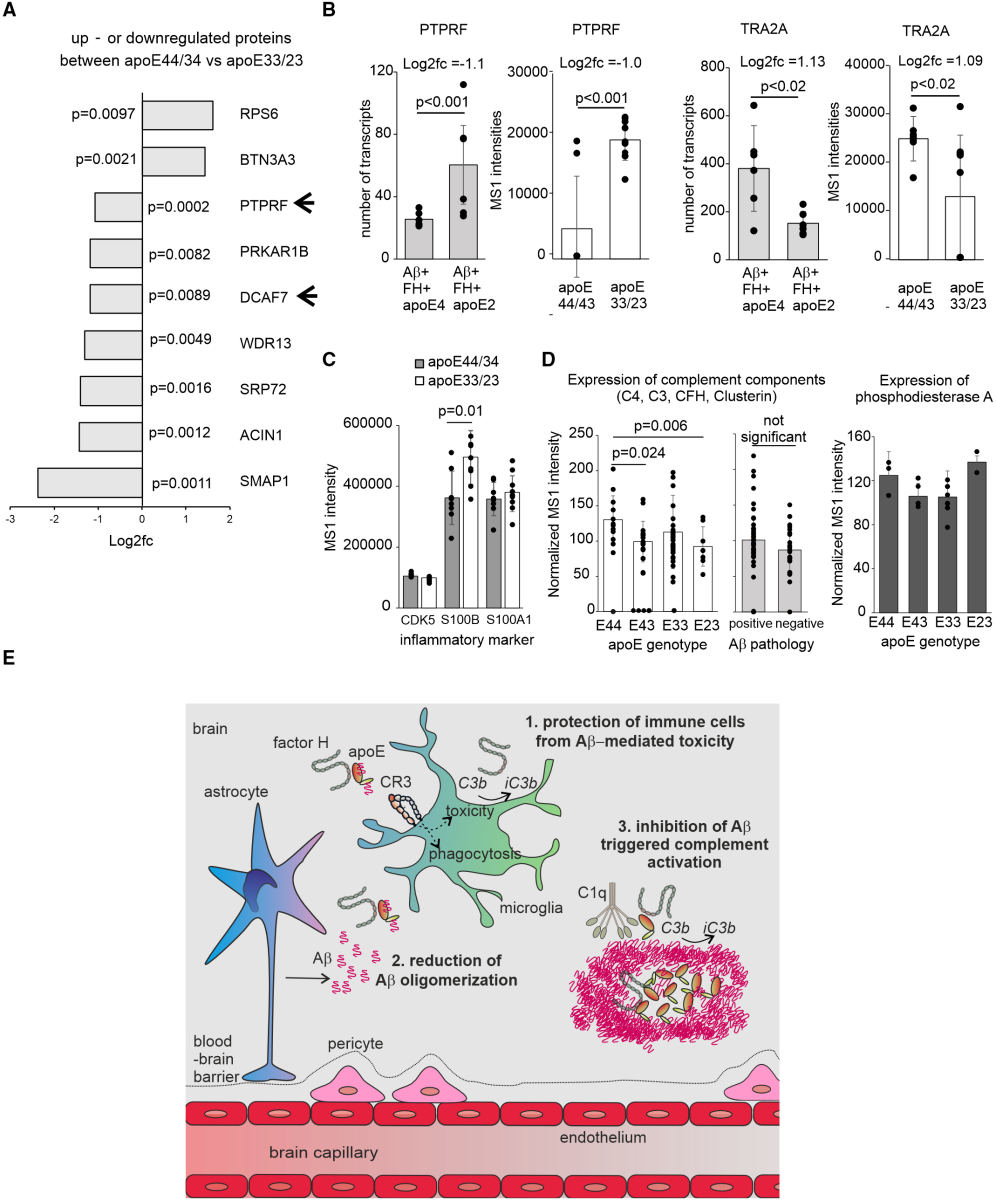
Complement activation markers and proteins that correlate with the presence apoE4 risk allele Top 9 proteins that correlate with the presence of apoE4 risk allele in iNPH biopsies between apoE44 or apoE43 (*n* = 8) and apoE33 or apoE23 (*n* = 9) carriers (See Appendix Table S1 for patient data and all DE proteins in Dataset EV3). (arrow) Two of the proteins also showed differential expression *in vitro* (Fig 5B)Comparison of mRNA transcripts (two individual samples obtained from three times repeated experiments) and MS1 intensities (from 8 apoE44/apoE43 and 9 apoE33/23 carriers) between genes or proteins that showed differential expression *in vitro* (Fig 5B) and *in vivo*.Analysis of the key AD neuroinflammatory markers obtained from the detected MS1 spectra between apoE44 or apoE43 (*n* = 8) and apoE33 or apoE23 (*n* = 9) carriers.(left) Combined analysis of complement activation markers C3, C4, CFH, and Clusterin obtained from the detected MS1 spectra between samples from apoE44 (*n* = 3), apoE43 (*n* = 5), apoE33 (*n* = 7) and apoE23 (*n* = 2) carriers (four markers/patient). (center) Combined analysis of complement activation markers C3, C4, CFH and Clusterin obtained from the detected MS1 spectra between samples that were positive (*n* = 10) or negative (*n* = 7) for Aβ pathology (four markers/patient). (right) Expression levels of Phosphodiesterase A control, showing similar expression levels in frontal cortex, between samples from apoE44 (*n* = 3), apoE43 (*n* = 5), apoE33 (*n* = 7) and apoE23 (*n* = 2) carriers The data were normalized against neutral apoE33 samples with no apoE association.Schematic presentation of the role of isoform‐specific binding of apoE to FH in resolution of Aβ‐mediated inflammation that is impaired in the presence of the AD‐associated apoE4 isoform as suggested by the *in vitro* and *in vivo* findings. Data information: (A and B) Fold changes (Log2fc) and *P*‐values (shown) calculated using the DEP R package (Zhang *et al*, 2018). (C and D) Significances were calculated using two‐tailed one‐way ANOVA supplemented with Dunnett's multiple comparison tests using SPSS software. Source data are available online for this figure.

**Figure EV5:**
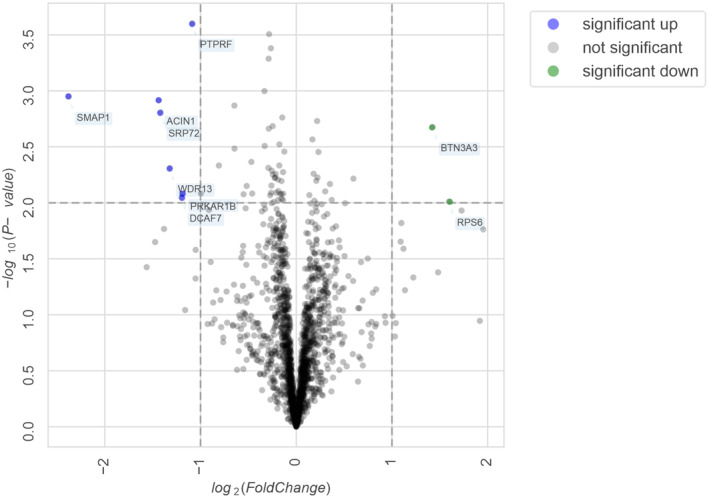
Volcano plot of differentially expressed proteins. Related to Fig 6A The (x‐axis) log2 fold change represents the difference between the levels of expression for each protein while (y‐axis) −log10 (*P*‐values) represents significance of each protein. Proteins with statistical significance and ± 1 log2 fold change are shown.

To have a full scan of local proteomic changes affected by the apoE risk isoform *in vivo*, the frontal cortex biopsy samples from iNPH patients that were apoE4 carriers (apoE44 or apoE43) and non‐carriers (apoE33 or apoE23) were subjected to mass spectrometry (Fig 6, Dataset EV3 and Fig EV5). Analysis of the total proteome and comparison between the apoE4 carriers and non‐carriers revealed up‐ or downregulation of several proteins, from which three (PTPRF, TRA2A, and NISCH) correlated very well with the *in vitro* mRNA data (Figs 5B and 6A and B; Dataset EV2). From these, upregulation of PTPRF is involved in cell adhesion pathway upon response to complement C3a (Jiang *et al*, 2021). PTPRF and DCAF7 also correlate with Aβ pathology in a recent iNPH transcriptomic data indicating a major role of these proteins in apoE isoform and complement‐dependent inflammatory response (Huang *et al*, 2021a). Screening of the MS1 and MS2 scans revealed three key inflammatory markers (CDK5, S100B, and S100A) from which S100B was significantly increased in apoE33/23 samples (Fig 6C; Yang, 2019). Interestingly, S100B is a proinflammatory protein that has been shown to suppress Aβ aggregation and toxicity (Cristovao *et al*, 2018). Four abundant complement activation markers that were detected showed increased levels in apoE44 samples (Fig 6D). Importantly, these markers did not show any correlation with Aβ pathology suggesting that the Aβ triggered complement‐mediated inflammatory response is apoE isoform‐dependent and thereby supports very well our hypothesis on the role of FH in reducing Aβ‐mediated inflammation.

## Discussion

The crucial role of FH in preventing complement attack toward host cells is exemplified by the mutations in FH found in atypical hemolytic uremic syndrome (aHUS), where lack of AP regulation leads to endothelial damage and microvascular thrombosis (Jokiranta, 2017). Because FH is located in fluid phase, it can easily reach areas where complement activation is exacerbated, such as Aβ plaques. The results of this work provide evidence on the concerted role of FH and apoE in reducing Aβ oligomerization and signs of neuroinflammation as these are promoted in the presence of the AD‐associated apoE4 isoform. Our data from both *in vitro* and *in vivo* analysis suggest that this mechanism may affect the early stage of amyloidogenesis through inhibition of Aβ toxicity, by reducing Aβ interaction with cells and phagocytosis of toxic Aβ (Step 1 in Fig 6E), by reducing Aβ oligomerization, and by forming complement resistant FH/apoE/Aβ complexes (Step 2 in Fig 6E). FH colocalizes with C1q on apoE‐coated surfaces (Step 3 in Fig 6E), suggesting yet another mechanism for target discrimination by FH. Here, high competitiveness of C1q and low competitiveness of FH to apoE4 predisposes the surface to C1q‐mediated complement activation. Finally, colocalization of apoE and FH on surfaces and small capillaries in the vicinity of Aβ plaques suggests that FH‐mediated complement regulation is involved in apoE isoform‐specific protection of BBB breakdown (Bell *et al*, 2012). As FH was abundantly detected around capillaries some of the detected FH in brain parenchyma may have been originated from circulation as leakage of blood‐derived proteins have been detected in apoE4 AD post‐mortem brain tissues (Zipser *et al*, 2007). Importantly, all these events are affected by the apoE isoforms, which therefore links FH to the most significant genetic risk factor for AD. FH is the main molecule keeping a delicate balance between complement activation and inhibition. Any disturbance in this balance, such as failure in binding of FH to apoE4 suggested in this study, will eventually lead to inflammation. Our immunohistochemistry and MS analysis using iNPH biopsy samples from living patients correlated well with our *in vitro* data and revealed a restricted set of novel and recently published molecules that may play an important role in AD pathogenesis. As earlier described, these samples represent early AD pathology and correlate well (but not completely) with mouse *in vivo* transcriptomic data but not with data obtained from human post‐mortem biopsy samples from AD patients (Huang *et al*, 2021a). In this regard, our data do not include any artifact related to post‐mortem changes or differences between species.

Under favorable conditions, Aβ monomers can form non‐fibrillar and fibrillar aggregates both in the extracellular space and intracellularly. Because of the different structure between Aβ monomers and fibrillar Aβ, the soluble monomers are more easily cleared by phagocytosis, action of secretases or delivery through the BBB (Zhao *et al*, 2015). In contrast, fibrillar aggregates accumulate, activate the complement system and induce inflammation. Our finding that FH forms complement‐resistant complexes especially with apoE2/Aβ1‐42, which have significantly smaller size than apoE/Aβ1‐42 complexes alone, suggests a role of this interaction in inhibiting Aβ‐induced complement‐mediated inflammation and Aβ aggregation. Based on the structural MS crosslinking data FH binds to apoE receptor binding site while the residue 402 in FH is not located near this apoE‐FH interaction site. Therefore, it is unlikely that the AMD‐associated 402H isoform would affect the ability of FH to reduce Aβ/apoE oligomerization. While binding of apoE to TREM2 has been shown to facilitate uptake of Aβ (Yeh *et al*, 2016), we show here that apoE and FH both inhibit binding of soluble Aβ1‐42 to activated CR3 and reduce, but does not completely inhibit, uptake of Aβ1‐42 by phagocytic cells. Because we observed reduced uptake of Aβ1‐42 also independently of CR3, it is possible that apoE and FH direct a controlled uptake of Aβ through receptors mediating non‐inflammatory signals such as TREM2. Interestingly, we observed differential gene expression between microglial cells incubated with FH + apoE2 and FH + apoE4, indicating differential signaling by apoE variants, as suggested by others (Huang *et al*, 2019). Importantly, when binding to its ligands, CR3 is capable of triggering Aβ‐induced microglial activation and can potentially constitute another mechanism of Aβ neurotoxicity (Zhang *et al*, 2011). Furthermore, it has been suggested that CR3 may be a potential therapeutic target for the treatment of AD, as knocking out of CR3 has been shown to decrease Aβ deposits in the brain of APP‐transgenic mice and increase extracellular Aβ degradation by microglia (Czirr *et al*, 2017).

There is a lack of understanding of how exactly Aβ is accumulated and cleared in the brain, but the role of microglia in Aβ clearance is less important than previously suggested, as 70% of extracellular Aβ is cleared by the BBB (Iliff *et al*, 2012). It is possible that the acidic environment in phagolysosomes may induce formation of Aβ fibrils that are compacted into dense‐core ‘indigestible’ material and therefore difficult to phagocytose (Huang *et al*, 2021b). Our study shows that FH and apoE2 reduce Aβ1‐42 phagocytosis along with a significant reduction in cell permeability. In the brain, apoE has an important role in maintaining lipid transport and membrane repairing. It has been suggested that Aβ toxicity toward cells may be due to its ability to modulate lipid membrane function (Legleiter *et al*, 2011). Therefore, controlled uptake of Aβ to cells by apoE and FH could protect the cells from the direct toxic effect of Aβ.

Binding of C1q to apoE on surfaces via the globular domains triggers CP activation (Vogt *et al*, 2020). We show here that binding of apoE to FH is isoform specific, but not to C1q. This indicates that apoE has a similar physiological function as CRP in acting locally at sites of inflammation to recruit FH and limit complement activation. In plasma, binding of C1q to CRP induces complement activation and C3b formation on damaged cells. Here FH is known to bind CRP, which blocks the AP activation and inflammation caused by the CP complement attack (Mihlan *et al*, 2009). Importantly, formation of C3b through C1q triggered complement activation is not sufficient for CR3‐mediated clearance by microglial cells as factor I‐mediated cleavage of C3b in the presence of a cofactor is necessity to form the CR3 ligand iC3b. It has been shown that C1q and Aβ play a role in synaptic pruning in early AD that is dependent on CR3‐mediated microglial engulfment of synapses (Hong *et al*, 2016). The reduced binding of FH to apoE4, shown in this study, could play a major role in the formation of iC3b that mediates CR3‐dependent phagocytosis of synapses. In conclusion, our studies have provided novel insights into the mechanism of AD pathogenesis, which may have implications in facilitating studies aiming to find molecular targets for drug discovery to prevent neurodegeneration.

## Materials and Methods

### Patient material

Right frontal Cortical Biopsy samples and plasma were obtained from patients with idiopathic normal pressure hydrocephalus (iNPH). Informed consent was obtained from all subjects in accordance with the Declaration of Helsinki and the Kuopio University Hospital Research Ethics Board approval (276/2016, 8‐Sep‐2020). Biopsy samples from patients with iNPH were taken prior to the insertion of the ventricular catheter of the cerebrospinal fluid (CSF) shunt as previously described (Koivisto *et al*, 2016).

### Isolation of HDL from plasma and detection of FH

HDL was isolated from plasma of iNPH patients with apoE23 (*n* = 2) or apoE44 (*n* = 2) genotype by sequential flotation in an ultracentrifugation using potassium‐bromide for density adjustment as previously described (Syed *et al*, 2021). The methodology of the preparations was as follows: Plasma VLDL and LDL (*d* < 1.063 g/ml) and total HDL (1.063 < *d* < 1.210 g/ml). Isolated HDL was dialyzed against phosphate‐buffered saline (PBS pH 7.4), visualized by SDS–PAGE, and checked for purity (Fig EV1G). Protein concentrations of the samples were measured with the Bicinchoninic acid assay (BCA; Pierce Biotechnology, Rockford, IL, USA). For Western Blotting (WB), 50 μg of each sample was run on gel as previously described under reducing conditions and transferred (iBlot, Thermo Fischer Scientific) to nitrocellulose membrane (iBlot™ 2 Transfer Stack, Invitrogen) and the membrane was blocked with 3% fat‐free milk in PBS for 2 h at room temperature. To detect FH from HDL fractions, the membrane was incubated with 1:2,000 diluted goat anti‐factor H (Cat# 341276, Millipore) in 0.3% fat‐free milk in PBS for 1 h at room temperature, washed three times with PBS, and incubated with 1:5,000 diluted IRDye® 800CW labeled anti‐Goat IgG, (Cat# 926‐32213, LI‐COR) for 1 h at room temperature. The membrane was washed with PBS and imaged using Odyssey® CLx Imaging System (LI‐COR).

### Proteins

Recombinant fragments of FH 1‐4, 5‐7 402Y, 5‐7 402H and 19‐20 were expressed in *Pichia pastoris* and purified as described earlier (Nissilä *et al*, 2018). The recombinant fragments of apoE2(1–165) and apoE4(1–165) were expressed in *E. coli* BL21‐Gold (DE3) and purified as described earlier (Dafnis *et al*, 2016). Mammalian apoE2 and apoE4 were expressed and purified from the culture medium of adenovirus‐infected HTB‐13 cells as described earlier (Chroni *et al*, 2008). FH402H and FH402Y were isolated from EDTA plasma collected from FH402Y or FH402H homozygous healthy donors with the permission of the ethical review board of the Hospital District of Helsinki and Uusimaa, Finland (448/13/03/00/09) as described earlier (Haapasalo *et al*, 2008).

### Co‐immunoprecipitation

ApoE‐genotyped HDL particles isolated from one iNPH patient (apoE23) were mixed with FH (Cat#A137, Complement technology) and diluted in PBS so that the final concentration of HDL particles is 0.5 mg/ml (total protein concentration) and that of FH is 0.04 mg/ml. This mixture was incubated at 37°C for 30 min. Meanwhile, mouse monoclonal anti‐FH antibody, 3D11 (Fontaine *et al*, 1989; or PBS as a negative control) was bound to protein G‐coupled Dynabeads (Cat# 10003D, ThermoFisher) by incubating them at 37°C for 10 min with rotation. After one wash with PBS, the beads were incubated with the preincubated HDL‐FH sample at 37°C for 30 min with rotation. The beads were then washed by incubating them with PBS plus 0.05% Tween 20 at 37°C for 45 min with rotation to dissociate lipids and proteins comprising HDL particles from apoE that were bound to FH. After one more quick wash with PBS plus 0.05% Tween 20, proteins were eluted from the beads by 50 mM glycine pH 2.7 and immediately neutralized by adding 2 M Tris–HCl pH 7.5. The eluted sample was mixed with Bolt LDS Sample Buffer and Bolt Sample Reducing Agent (ThermoFisher), incubated for 5 min at 95°C and run on PAGE gels (Mini‐PROTEAN TGX Stain‐Free Protein Gels, 4–20%, Bio‐Rad, 60 min, 140 V) in TGS buffer (Bio‐Rad). Proteins were transferred into nitrocellulose membranes, and the membrane was blocked with 3% fat‐free milk in PBS for 2 h at room temperature. After one wash with PBS, the membrane was incubated with 0.3% fat‐free milk in PBS containing 1:10,000 diluted goat polyclonal anti‐FH antibody (Millipore) and 1:5,000 diluted rabbit polyclonal anti‐apoE antibody (kind gift from Dr. Matti Jauhiainen. The antibody was raised in New Zealand White rabbit) for 1 h at room temperature. The membrane was then washed three times with PBS and incubated with 0.3% fat‐free milk in PBS containing 1:5,000 diluted IRDye 800CW donkey anti‐goat IgG secondary antibody (Cat# 926‐32214, LI‐COR) and 1:5,000 diluted IRDye 680RD donkey anti‐rabbit IgG secondary antibody (Cat# 926‐68073, LI‐COR) for 1 h at room temperature. After three washes with PBS, the presence of FH and apoE was detected by using Odyssey CLx imaging system (LI‐COR).

### Binding of FH to HDL and competition with apoE2(1–165) and apoE4(1–165)

To detect the binding of FH to HDL and assess the competition with apoE isoforms, apoE2 (1–165) and apoE4 (1–165), HDL was coated on 96‐well plates (SpectraPlate‐96 HB, PerkinElmer) at concentration of 20 μg/ml in 50 mM bicarbonate buffer pH 9.6 overnight at +4°C. The wells were blocked with 3% fatty acid‐free BSA (Cat#6156, Biowest) in PBS for 2 h at room temperature. After one wash with PBS 20 nM FH (Complement technology) was incubated on plate with 1:3 dilution series of apoE2(1–165) and apoE4(1–165) starting at 200 nM concentration in 0.3% BSA/PBS for 1 h 30 min at 37°C. After wells were washed for three times with PBS, FH was detected with 1:2,000 diluted goat anti‐Factor H (Millipore) in 0.3% BSA/PBS by incubating for 1 h at 37°C. Next, wells were washed three times with PBS and incubated with 1:2,000 dilution of HRP conjugated anti‐goat IgG antibody (Cat# 705–035‐147, Jackson ImmunoResearch Laboratories) in 0.3% BSA/PBS at 37°C for 1 h. After three washes with PBS, *o*‐phenylenediamine dihydrochloride (OPD) substrate (Cat# 34006, Thermo Scientific) was added according to manufacturer instructions. The reaction was stopped with 0.5 M H_2_SO_4_ and absorbance was measured at 492 nm.

### Microscale thermophoresis (MST)

Binding between FH (Complement technology), FH402Y, FH402H, FH fragments 1–4, 5–7 (402H and 402Y), 19–20, apoE2(1–165) and different apoE isoforms (Cat# OPBG00054, OPBG00055 and OPBG00056; Aviva systems biology) was studied by MST. FH and apoE proteins were labeled with NT647 dye using Monolith NT Protein Labeling kit RED‐NHS according to the manufacturer's instructions (NanoTemper; degree of labeling 1.0–0.8 moles dye per mole protein). Increasing concentrations of unlabeled proteins in PBS were mixed with 5–30 nM of the labeled proteins in 150 mM KCl, 25 mM HEPES pH 7.5 (Gibco), 0.01% Tween 20, 0.01% NaN_3_. The samples were scanned in the premium‐coated monolith NT capillaries using a NanoTemper Monolith NT.115Pico instrument (20–50% LED, 40% IR‐laser power), and binding affinities were analyzed and evaluated using MO. Affinity Analysis software (NanoTemper).

### Cell culturing

Human SV40 immortalized microglia cells (ABM, Cat#T0251) were cultured in DMEM GlutaMAX containing 4.5 g/l glucose (Gibco) supplemented with 10% fetal bovine serum (FBS; Gibco) and 1% penicillin–streptomycin (Gibco). The flasks were precoated with rat type I collagen (Gibco) in 20 mM acetic acid and washed with DPBS (Gibco). Our CR3 (CD11b/CD18)‐overexpressing U937 cell line (U937‐CR3) is based on U937, a pro‐monocytic, human myeloid leukemia cell line from American Type Cell Culture (ATCC), in which CR3 receptor is stably expressed using a lentiviral expression system and the vector constructs as described earlier (Nissilä *et al*, 2018). U937‐CR3 cells were cultured in RPMI‐1640 (Lonza) medium supplemented with 2 mM Glutamine, 10% FBS and 1% penicillin–streptomycin. To maintain the stable expression of CR3 (CD11b‐ and CD18‐expressing vectors carrying zeocin and puromycin resistance genes, respectively), puromycin 2 μg/ml (Calbiochem) and zeocin 200 μg/ml (Invitrogen) were added to the cell culturing media (Nissilä *et al*, 2018). To obtain macrophages, U937 or U937‐CR3 cells were incubated in the presence of 150 nM PMA (phorbol 12‐myristate 13‐acetate, from Sigma) for 72 h. All cell lines have been tested for mycoplasma contamination regularly using MycoStrip™ Mycoplasma Detection Kit (InvivoGen).

### CR3‐receptor binding assay

In order to understand how the molecules of interest bind CR3, we induced bent inactive and extended open active conformations of CR3 by treating the U937 cells with Hank's balanced salt solution (HBSS) without Ca^2+^ and Mg^2+^ (Gibco) and 5 mM EDTA, or with HBSS, 10 mM HEPES, 2 mM EGTA and 0.5 mM MnCl_2_, respectively (Chen *et al*, 2010). To study binding of apoE2 and apoE4 to CR3, apoE was labeled by DyLight™ 405 (Thermo Fisher Scientific) according to manufacturer's instructions. Preincubation of DyLight™ 405‐labeled apoE (degree of labeling 0.25 moles dye per mole protein) with FH, Aβ1‐42 (Cat#AS‐20276, Anaspec Inc.), FH5‐7, FH19‐20 or molecular biology grade bovine serum albumin (BSA; New England BioLabs) or without any of them was carried out in 96‐well round‐bottom plates in 5 mM EDTA‐ or 1 mM Mn^2+^‐containing buffer (described above) for 15 min at room temperature. Then 1.4 × 10^5^ U937 or U937‐CR3 cells that were pre‐washed with the corresponding buffer were added to wells. The final concentration for each preincubated molecule was adjusted to 300 nM. The mixture was incubated for 45 min at 4°C in the dark with gentle shaking. To study binding of HiLyte™ Fluor 488‐labeled Aβ1‐42 (Cat#AS‐60479‐01, AnaSpec Inc.) to CR3, first 645 nM apoE2 and apoE4 with or without 645 nM FH, only 645 nM FH, only 645 nM FH19‐20 or only 645 nM BSA were preincubated in EDTA‐ or Mn^2+^‐containing buffer (described above) for 15 min at 21°C. Next, 1.4 × 10^5^ of U937 or U937‐CR3 cells were pre‐washed with the corresponding buffer, added to wells, and incubated for another 15 min on ice. After adding 4.4 μM of HiLyte™ Fluor 488‐labeled Aβ1‐42, the mixture was incubated for 45 min at 4°C in the dark with gentle shaking. After incubation, the wells were washed with corresponding buffer once and fixed with 1% (v/v) paraformaldehyde. Next, cells were run to BD LSRFortessa flow cytometer and analyzed using FlowJo V10 software (FlowJo, LLC).

### Microglial phagocytosis and mRNA sequencing

Preincubation of 1.4 μM of apoE2, apoE3, or apoE4 with or without 650 nM FH (Complement Technology, Inc.) was performed in 96‐well round bottom plates (Thermo Fisher Scientific) for 30 min on ice. HiLyte488™ Fluor‐labeled Aβ1‐42 was incubated in the dark at room temperature in PBS for 72 h and then mixed with microglial cells (2.5 × 10^6^ cells/ml). The suspension was immediately pipetted into the wells containing the preincubated mixture of apoE and FH (final concentration of Aβ: 2 μM, final amount: 8.7 × 10^4^ microglial cells per well). Samples were further incubated for 1 h at 37°C with gentle shaking in RPMI‐1640 media (Gibco) supplemented with 0.05% human serum albumin (HSA, Sigma). Samples were divided for the mRNA sequence analysis and for the flow cytometric analysis. For the mRNA sequence analysis, 3 × 10^4^ cells were stabilized by RNAlater (Thermo Fisher). RNA sequencing method was designed based on the Drop‐seq protocol and carried out as described earlier (Nissilä *et al*, 2018). For flow cytometry, the phagocytosis was stopped by adding and washing the samples with ice‐cold RPMI‐1640 with 0.05% HSA. Samples were fixed with 1% (v/v) paraformaldehyde (Thermo Fisher) in RPMI‐1640 with 0.05% HSA. Next, cells were run to BD LSR Fortessa flow cytometer and analyzed using FlowJo V10 software (FlowJo, LLC).

### Single‐molecules TIRF microscopy

For stoichiometric studies, Cys thiols in apoE2 were labeled with Maleimide C5 Alexa568 reagent according to manufacturer's instructions (Thermo Fisher Scientific). The degree of labeling was 1 as determined by protein concentrations using absorbance at 280 nm and dye concentrations using absorbance at 579 nm by a Nanodrop ND‐1000 spectrophotometer. Protein mixture of 1 μM Maleimide C5 Alexa568‐apoE2, 18.5 μM HiLyte 488‐Aβ1‐42 with or without 1.7 μM FH and was incubated for 72 h at room temperature in the presence of 0.14 mM DTT in Protein LoBind® Tubes (Eppendorf). Imaging was performed on a bespoke single‐molecule TIRF microscope constructed around a Nikon Ti‐E microscope body, using Obis 488 and 561 nm lasers set to 20 mW, beam expanded to fill the field of view of Photometrics Evolve 512 at 100 nm/pixel with a 100× Nikon 1.49 NA TIRF objective lens. Pre‐incubated solutions of labeled Aβ1‐42, apoE2 and FH were imaged in simple flow cells constructed from glass slides, plasma‐cleaned coverslips, and double‐sided tape, coated with 5 μg/ml of anti‐Aβ mAb (clone H31L21, Invitrogen). Multiple fields of view and samples were imaged to generate > 10,000 foci tracks over 1,000 frames at 50 ms/frame exposure time. Data were analyzed using bespoke MATLAB software (Haapasalo *et al*, 2019) to track and quantify the intensity of foci as a function of time. Briefly, centroids are determined using iterative Gaussian masking and intensity calculated using the summed intensity inside a foci corrected for the local background in a small square region of interest around the foci. Foci were accepted if their signal‐to‐noise ratio was above 0.4 and lasted longer than three frames. Foci were deemed colocalized if their overlap integral was over 0.75. Stoichiometries were determined by first calculating the intensity of single dyes using photobleaching analysis to use only single fluorophores (Fig EV4A and B). Step‐wise intensity traces found within the first 10 frames (Fig EV4C) were photobleach‐corrected using a linear regression of the first 4 points, which we have shown is equivalent to full exponential fitting (Shashkova *et al*, 2021). Initial intensities were divided by characteristic intensity to obtain stoichiometries.

### Oligomerization studies

Different combinations of 3 μM apoE2/3/4, 3 μM FH or 3 μM FH fragments (5–7 or 19–20) were incubated with and without 28 μM Aβ1‐42 (AnaSpec Inc.) for 72 h at room temperature in PBS in the presence of 0.14 mM DTT in Protein LoBind® Tubes (Eppendorf). To separate protein complexes, complexes and monomers, the samples were run through gel filtration column (Superdex 200 10/300 GL column, Global Life Sciences Solutions LLC) at 0.5 ml/min flow rate in PBS. Fractions of 300 μl were collected, and the fractions were stored immediately at −80°C until further analysis. The peaks from size exclusion chromatography were analyzed by ELISA to verify the presence or absence of each protein in the complexes (Fig EV3A). The size of the complexes and single molecules was estimated by running the Bio‐Rad Gel Filtrations Standard (Cat#1511901) using the same protocol. For PAGE analysis, the protein mixtures were mixed with Bolt LDS Sample Buffer (Thermo Fisher Scientific) or 2 × native sample buffer (1×: 62.5 mM Tris–HCl pH 7.4, 25% glycerol, 1% bromophenol blue), run on PAGE gels (Mini‐PROTEAN TGX, 4–20%, Bio‐Rad, 60–90 min, 100 V) in Tris‐glycine buffer or TGS buffer (Bio‐Rad) and detected by silver staining or WB. For WB the proteins were transferred into nitrocellulose membranes, and the membrane was blocked with 3% fat‐free milk in PBS for 2 h at room temperature. Next, the membrane was incubated with 1:1,000 diluted rabbit anti‐Aβ mAb (clone H31L21, Invitrogen) in 0.3% fat‐free milk in PBS for 1 h at room temperature, washed three times with PBS and incubated with 1:5,000 diluted IRDye 800CW‐conjugated goat anti‐rabbit IgG (LI‐COR) for 1 h at room temperature. After washing the presence of Aβ complexes was detected using Odyssey® CLx imaging system (LI‐COR).

### Complement activation and inhibition

To couple protein complexes into latex beads the protein mixtures of 3 μM apoE2, apoE3, or apoE4, 3 μM FH or 3 μM BSA were incubated with and without 30 μM HiLyte™ Fluor 488‐labeled Aβ1‐42 for 72 h at room temperature in PBS in the presence of 0.14 mM DTT. Twenty microliters of these samples were coupled to 56 mg of carboxyl latex beads (Thermo Fisher, 4% w/v, 2 μm diameter, C37278). Before coupling the beads were washed twice with sterile MES‐buffer (0.025 M (2‐(*N*‐morpholino)ethanesulfonic acid), pH 6.0) and centrifuged between washes at 3,000 *g* for 20 min. The latex bead‐protein mixtures were incubated overnight with gentle mixing at room temperature covered from light. Next, beads were centrifuged at 3,000 *g* 20 min, and the coupling efficiency was calculated by measuring the ratios between A280 in the supernatant and the original sample using NanoDrop 1000 (Thermo Scientific). Coupling efficiency was above 80% in each sample. Next, coupled beads were washed three times with PBS and resuspended in 50 μl of PBS. To study complement activation capacity of the coupled beads, 4 μl of the bead mixtures were incubated in the presence of 0.75 μM C3 (Cat#A113, Complement technology), 0.75 μM factor B (Cat#A136, Complement technology), 0.075 μM factor D (Cat#A135, Complement technology) and 0.375 μM factor I (Cat#A138, Complement technology) in Ca^2+^/Mg^2+^ free Dulbecco's phosphate‐buffered saline (D‐PBS) with 1 mM of MgCl_2_ (Mg‐DPBS) for 60 min at 37°C. After incubation, the beads were centrifuged at 3,000 *g* for 20 min. The beads were again washed twice with PBS, resuspended to 20 μl PBS and stored at 4°C for phagocytosis assay and iC3b WB. C3a was measured from 1:10 diluted supernatant by following the manufacturer's instructions (MicroVue C3a Plus, Quidel). Formation of iC3b on the washed beads was detected by SDS–PAGE and WB. First, 6.5 μl of beads were incubated with 2.5 μl Bolt™ LDS Sample Buffer and 1 μl Bolt™ Sample Reducing Agent (Thermo Fisher) for 10 min at 95°C. Next, the samples were centrifuged at 3,000 *g* for 20 min, and 5 μl of supernatant was run on PAGE gels (Mini‐PROTEAN TGX, 4–20%, Bio‐Rad, 60–90 min, 100 V) in TGS buffer (Bio‐Rad) including 100 ng of control C3, C3b (Cat#A114, Complement technology) and iC3b (Cat#A115, Complement technology) under reducing conditions. The proteins were transferred into nitrocellulose membranes, and the membrane was blocked with 3% fat‐free milk in PBS for 2 h at room temperature. To detect C3 cleavage fragments, the membrane was incubated with 1:130 diluted rabbit anti‐C3c antibody (Cat# OSAP 14/15, Behring) in 0.3% fat‐free milk in PBS for 1 h at room temperature, washed three times with PBS, and incubated with 1:5,000 diluted IRDye 800CW‐conjugated goat anti‐rabbit IgG (LI‐COR) for 1 h at room temperature. After three washes the presence of C3 cleavage fragments was detected by using Odyssey^®^ CLx imaging system (LI‐COR).

### Phagocytosis of beads coated with protein complexes

U937 cells were differentiated to macrophages for 72 h using 150 nM of PMA, washed with DPBS (Gibco) and incubated for 10 min at 37°C with cell stripper (Corning). Detached cells were removed by resuspending the cells carefully in RPMI‐1640 with 0.05% HSA. After centrifugation for 10 min at 300 *g*, the cells were resuspended at a density of 5 × 10^6^ cells/ml, and the presence of 95% or more live cells were calculated using trypan blue (Bio‐Rad). Next, 1 μg/ml of DAPI was added to the cells, and 10 μl of beads incubated with complement components were mixed with 40 μl of these cells. For compensation cells without DAPI were incubated with 1.2 μM HiLyte™ Fluor 488‐labeled Aβ1‐42, and cells with DAPI were incubated with 1.2 μM of non‐labeled Aβ1‐42. Also, beads coated with only HiLyte™ Fluor 488‐labeled Aβ1‐42 without complement deposition were subjected to the assay to measure the effect of C3b and iC3b deposition on phagocytosis. Samples were incubated for 1 h at 37°C with 5% CO_2_ atmosphere with mild shaking. Phagocytosis was stopped with 300 μl of cold RPMI‐1640 with 0.05% HSA, and cells were centrifuged as above and fixed with 200 μl of 1% formaldehyde in RPMI‐1640 with 0.05% HSA. Eighty microliters of cells were diluted into 200 μl of ice‐cold PBS, run on FACS flow cytometer (2,000–10,000 cells/sample), and analyzed to obtain fluorescent % and mean on all samples.

### Binding of FH and C1q to apoE or apoE/Aβ by ELISA

To study the binding of C1q and FH to apoE‐coated wells, 10 μg/ml apoE2/apoE3/apo4 (*E. coli* expressed) or apoE2/apo4 (mammalian cell expressed) were coated on 96‐well plates (SpectraPlate‐96 HB, PerkinElmer) in 0.05 M bicarbonate buffer (pH 9.6) overnight at 4°C. To study binding of FH to apoE/Aβ increasing concentration of (0–540 nM) apoE2/apoE3/apoE4 was incubated with 6 μg/ml of Aβ1‐42 for 2 h in Protein LoBind® Tubes (Eppendorf) before coating on 96‐well plates. Wells were washed once with GVB^++^ (0.1% gelatin, 5 mM Veronal, 145 mM NaCl, 0.15 mM calcium chloride and 0.5 mM magnesium chloride, pH 7.3) before wells were blocked with 3% BSA (fatty acid free, Cat# 6156, Biowest) in GVB^++^ at room temperature for 2 h. After one wash with GVB^++^ 60 nM concentration of FH or C1q with or without different concentrations (0–60 nM) of C1q or FH were incubated on wells for 1 h at 37°C or overnight at 4°C. After three washes with GVB^++^ wells were incubated with 1:2,000 dilution of rabbit anti‐human C1q complement (Cat# A0136, Dako corporation) or goat anti‐factor H (Cat# 341276, Calbiochem) antibodies in GVB^++^ at 37°C for 1 h. Wells were washed three times with GVB^++^ and incubated with 1:2,000 dilutions of secondary antibodies anti‐Rabbit IgG HRP‐conjugated (Cat# PC2852‐1197, PerkinElmer Life Sciences) or HRP‐conjugated donkey anti‐Goat IgG (Cat# 705–035‐147, Jackson ImmunoResearch laboratories). After three washes with GVB^++^ OPD substrate (Cat# 34006, Thermo Scientific) or Ultra TMB‐ELISA substrate (Cat# 10647894, Fisher Scientific) was added according to manufacturer instructions. 0.5 M H_2_SO_4_ was used to stop the reaction and absorbance was measured at 492 nm or 450 nm.

### Immunofluorescence staining of frontal cortical biopsy samples

Biopsy samples were frozen in blocks of optimal cutting temperature (OCT, 25608‐930, Tissue‐Tek), sectioned at a thickness of 20 μm with a cryostat (Leica Microsystems, Wetzlar, Germany), collected on superfrost microscope slides (ThermoFisher Scientific, Waltham, USA) and stored at −70°C until analysis. First the iNPH biopsy samples were analyzed for the presence of Aβ plaques. After air dry, the sections were rehydrated and washed twice with phosphate‐buffer (PB) pH 7.4 and once with PBS for 5 min. For antigen retrieval, the sections were incubated for 45 min in preheated (92°C) 10 mM sodium citrate buffer, pH 6.0 and the buffer was let cool down at room temperature for 45 min. The slides were washed three times for 5 min with PBS containing 0.05% Tween‐20 (PBST; Sigma‐Aldrich, St. Louis, USA). Endogenous peroxidase was blocked using 0.3% H_2_O_2_ in MeOH for 30 min at room temperature before three washes as previously. The sections were blocked by 1 h incubation in 10% normal horse serum (Vector Laboratories Ltd., Burlingame, USA). Next, samples were incubated at room temperature with rabbit anti‐Aβ, (dilution 1:800, 8243s, Cell Signaling Technology) in 5% normal horse serum in PBST. After three 5 min washes with PBST, the samples were incubated for 2 h with biotinylated goat anti‐rabbit IgG antibody (dilution 1:200, BA‐1000, Vector Laboratories) in 5% normal horse serum in PBST. After three 5 min washes with PBST, the slides were incubated in ABC reagent (PK‐6101, Vector Elite Kit, Vector Laboratories) for 2 h at room temperature. After washes with PBST as above, the sections were incubated with Ni‐DAB in 0.075% H_2_O_2_ until the color developed. The reaction was stopped by washing in ddH_2_O twice for 7 min. The sections were dehydrated in ethanol gradient (50%, 70%, 95%, 100% EtOH) and 2 times with xylene for 1 min each and embedded using DePex (Serva) on coverslips.

For double and triple immunohistochemical staining the sections were air dried, washed, and boiled for antigen retrieval similarly to Aβ staining. Next, the slides were washed three times with PBST, blocked for lipofuscin with 1:20 dilution of TrueBlack® Lipofuscin Autofluorescence Quencher (Cat# 23007, Biotium, San Francisco, USA) in 70% ethanol for 1 min and then washed three times with PBST. The sections were blocked for 1 h in 5% BSA or 10% normal horse serum (Vector Laboratories Ltd., Burlingame, USA). The primary antibodies were incubated overnight at room temperature: goat anti‐factor H (dilution 1:400; Cat# 341276, Calbiochem/Sigma‐Aldrich, St. Louis, USA), rabbit anti‐apolipoprotein E (dilution 1:400; Cat# ab183597; Abcam, Cambridge, UK), rabbit anti‐C1q (dilution 1:300, Cat#A0136, Dako), mouse anti‐Aβ (dilution 1:1,000; clone WO‐2; Cat# MABN10, Millipore), rabbit anti‐Aβ (dilution 1:800; clone D54D2; Cat# 8243s, Cell signaling technology) and rabbit anti‐Iba1 (Cat#. 019‐19741, Fujifilm Wako) in 3% BSA or 5% normal horse serum in PBST. For double and triple IHC stainings, antibodies for FH and/or apoE together with antibody against Aβ were used. After washing in PBST, the sections were incubated with 1:500 dilution of Alexa Fluor 488 (chicken anti‐Rabbit IgG, Invitrogen, Cat# A21441), 488 (donkey anti‐goat 488 Invitrogen Cat#A11055), 555 (donkey anti‐mouse IgG, Invitrogen, Cat#31570), 594 (donkey anti‐goat IgG, Invitrogen, Cat# A11058) 594 (donkey anti‐rabbit 594 Invitrogen Cat# A21207) or 680 (donkey anti‐goat IgG, Invitrogen, A21084) secondary antibody (ThermoFisher Scientific, Waltham, USA) for 2 h at room temperature in 5% normal horse serum in PBST, washed again, and mounted in Vectashield with DAPI (Cat# H1200, Vector Laboratories Ltd., Burlingame, USA). Negative controls were included in parallel sessions following the same procedures without the incubation with primary antibodies. The confocal images were imaged under × 20, × 40 or × 64 magnification with Zeiss Axio Observer with Zeiss LSM 800 Airyscan confocal module (Carl Zeiss AG, Jena, Germany) and analyzed using the Zen software (all Carl Zeiss AG, Jena, Germany). For colocalization analysis and for measuring microglia Iba‐1 and Aβ intensities the DAPI channel was segmented using Otsu's method with any areas smaller than 50 pixels or holes removed using morphological transformations. Unique cell regions were defined based on each separate mask from the DAPI channel. A bounding box of 100 pixels was drawn around each mask and the whole region of interest correlation coefficient calculated between the channels or intensities for the microglia (Iba‐1), Aβ and FH, the summed background corrected pixel intensity was calculated.

### Mass spectrometry

The frontal cortex biopsy samples were from apoE genotyped iNPH patients that were analyzed for the presence or absence of Aβ plaques. These included three apoE44 Aβ positive, three apoE43 Aβ positive, two apoE43 Aβ negative, four apoE33 Aβ positive, three apoE33 Aβ negative and two apoE23 Aβ negative samples. All of the collected brain biopsy was taken for homogenization. Homogenization was done in 6.0 M urea/50 mM NH_4_HCO_3_ (Sigma‐Aldrich) by sonicating the samples in an ice bath with 15 min of continuous sonication followed by a 10 min break for a total of two cycles. Then samples were centrifuged at 14,000 *g* for 15 min at 4°C and supernatant was collected. For total protein concentration measurement urea concentration was diluted to 3 M with 50 mM NH_4_HCO_3_. Total protein concentration of the samples was measured with BCA protein assay kit (Pierce, Thermo Scientific) and 50 μg of total protein from each sample was taken for in‐solution digestion. The proteins were reduced with 5 mM DL‐dithiothreitol (Sigma Aldrich) alkylated with 15 mM iodoacetamide (Fluka), and trypsin‐digested with 2 μl Sequencing Grade Modified Trypsin (Promega) overnight at 37°C. Desalting was done with C18 MicroSpin Columns (The Nest Group, cat. no. SEMSS18V). After the C18 purified peptides were dried, the samples were resuspended into 30 μl of buffer A (1% ACN, 0.1% FA in HPLC grade H_2_O; all from Merck). Samples were diluted 1:50 prior to loading to EvoTips (EvoSep, Denmark). The total volume of 20 μl was pipetted into EvoTips before analysis by EvoSepOne with EvoSep EV1109 analytical column using a 60 samples/day method coupled to timsTOF Pro (Bruker, Germany) operated with DDA PASEF‐short gradient 0.5 s cycletime –method.

The resulting MS files were submitted to the FragPipe v16.0 software for protein identification and label‐free quantification. The FragPipe v16.0 included MSFragger v3.3 and Philosopher v4.0.0. The searches were performed with reviewed Human proteome UP000005640. Decoy sequences and common contaminants were generated and added to the original database as part of the FragPipe workflow. Trypsin was selected as the cleavage specificity and methionine oxidation and N‐terminal acetylation were set as variable modifications. Static residue modification was set for carbamidomethylation of cysteines. The allowed peptide length and mass ranges were 5–50 residues and 200–5,000 Da, respectively. Within FragPipe all peptide‐spectrum matches (PSMs), peptides, and proteins were filtered to 1% PSM and 1% protein FDR. For MSFragger precursor tolerance was set to 50 ppm and fragment tolerance was set to 20 ppm, with mass calibration and parameter optimization enabled. Two missed cleavages were allowed, and two enzymatic termini were specified. Isotope error was set to 0/1/2. The minimum number of fragment peaks required to include a PSM in modeling was set to two, and the minimum number required to report the match was four. The top 150 most intense peaks and a minimum of 15 fragment peaks required to search a spectrum were used according to recommended settings. Label‐free quantification was employed with default settings.

### Crosslinking of proteins

For crosslinking, 2 μg of FH 5–7 protein was mixed with 3 μg of apoE2 N‐terminal fragment (1–165) at 1:1 molar ratio in a final reaction volume of 20 μl in PBS and incubated for 15 min at 37°C, at 1,000 rpm shaking speed for the proteins to bind to each other. Heavy (deuterated) and light disuccinimidyl suberate (DSS‐H12/DSS‐D12, Creative Molecules Inc., 001S) was added to final concentrations of 0.25 mM and the samples further incubated for 60 min at 37°C, at 1,000 rpm shaking speed. The crosslinking reaction was quenched with a final concentration of 50 mM of ammonium bicarbonate for 15 min at 37°C, 1,000 rpm.

### Crosslinked sample preparation for mass spectrometry analysis

All samples for MS analysis were prepared by denaturing the proteins using 8 M urea – 100 mM ammonium bicarbonate solution. The cysteine bonds were reduced with a final concentration of 5 mM Tris(2‐carboxyethyl) phosphine hydrochloride (TCEP, Sigma, 646547) for 60 min at 37°C, 800 rpm and subsequently alkylated using a final concentration 10 mM 2‐iodoacetamide for 30 min at 22°C in the dark. For digestion, 1 μg of lysyl endopeptidase (LysC, Wako Chemicals, 125‐05061) was added, and the samples incubated for 2 h at 37°C, 800 rpm. The samples were diluted with 100 mM ammonium bicarbonate to a final urea concentration of 1.5 M, and 1 μg of sequencing grade trypsin (Promega, V5111) was added for 20 h at 37°C, 800 rpm. The digested samples were acidified with 10% formic acid to a final pH of 3.0. Peptides were purified and desalted using C18 reverse phase column following the manufacturer's recommendations (The Nest Group, Inc.). Dried peptides were reconstituted in a solution containing 2% acetonitrile and 0.1% formic acid prior to MS analysis.

### Liquid chromatography mass spectrometry for crosslink identification

A total of 500 ng of peptides were analyzed on an Orbitrap Eclipse mass spectrometer connected to an ultra‐high‐performance liquid chromatography Dionex Ultra300 system (Thermo Scientific). The peptides were loaded and concentrated on an Acclaim PepMap 100 C18 precolumn (75 μm × 2 cm) and then separated on an Acclaim PepMap RSLC column (75 μm × 25 cm, nanoViper, C18, 2 μm, 100 Å; both columns Thermo Scientific), at a column temperature of 45°C and a maximum pressure of 900 bar. A linear gradient of 2–25% of 80% acetonitrile in aqueous 0.1% formic acid was run for 100 min followed by a linear gradient of 25–40% of 80% acetonitrile in aqueous 0.1% formic acid for 20 min. One full MS scan (resolution 120,000; mass range of 400–1,600 *m/z*) was followed by MS/MS scans (resolution 15,000) of the 20 most abundant ion signals. Precursors with an unknown charge state, a charge state of 1, 2, or above 8 were excluded. The precursor ions were isolated with 1.6 *m/z* isolation window and fragmented using higher‐energy collisional‐induced dissociation (HCD) at a normalized collision energy of 30. The dynamic exclusion was set to 45 s.

### Crosslinking data analysis

All spectra from crosslinked samples were analyzed using pLink 2 (version 2.3.10; DOI 10.1038/s41467‐019‐11337‐z). The target protein database contained the sequence for the human FH 5–7 and ApoE2 proteins. pLink2 was run using default settings for conventional HCD DSS‐H12/D12 crosslinking, with trypsin as the protease and up to three missed cleavages allowed. Peptides with a mass range of 600–6,000 *m/z* were selected (peptide length of ca. 6–60 residues) and the precursor and fragment tolerance were set to 20 and 20 ppm, respectively. Crosslink identifications were filtered by requiring 10 ppm mass accuracy, false discover rate (FDR) < 5%, *E*‐value < 0.01, and ≥ 5 observed spectra, including spectra with both DSS‐H12 and DSS‐D12. The unfiltered crosslinking data are presented in Dataset EV1. All crosslinking data have been deposited to the ProteomeXchange consortium via the MassIVE partner repository https://massive.ucsd.edu/ with the dataset identifier PXD039369.

### Molecular docking and molecular dynamics simulations

The FH5‐7/ApoE2 complex was modeled with the crosslink data as restraints with HADDOCK v2.4 web server (https://wenmr.science.uu.nl/haddock2.4/; van Zundert *et al*, 2016) with maximum DSS crosslink distance as 30 Å (Merkley *et al*, 2014) between the crosslinked lysine residues. This resulted in five clusters, of which four representative structures of each cluster were submitted to atomistic molecular dynamics simulations in explicit solvent. These 20 structures were solvated, thoroughly energy minimized, equilibrated, and finally simulated using two complementary atomistic force fields, CHARMM36m (Huang *et al*, 2017) and Amber FF14SB (Maier *et al*, 2015), for 250 ns each using the GROMACS 2021 simulation engine (Pall *et al*, 2020). The recommended simulation parameters for these force fields were used. The stability of the interfaces was assessed by the mean RMSD of FH5‐7 during the last 50 ns of simulation after first RMSD‐fitting apoE2 the structure to its initial conformation. For the most stable structure, the key hydrogen bonds predicted by both force fields were analyzed. For details on the simulation setup and simulation parameters, see Fig EV2.

### Statistical analysis

For MS1 intensity fold change calculations, samples were grouped based on *APOE* genotypes. After discarding proteins for which no valid values in at least 50% of one group were found, the MS1 intensities were log2 transformed, missing values were imputed from a normal distribution using the QRILC method, and fold changes and *P*‐values calculated using the DEP R package (Zhang *et al*, 2018). To analyze expression of complement activation markers, the MS1 intensities of the markers were normalized against neutral apoE33 samples, and the combined intensity values were compared. Statistical significances were calculated from experiments that were performed using at least three biological replicates by SPSS statistics (SPSS version 24 IBM Statistics). First, Kolmogorov–Smirnov normality test was used to analyze, whether variables were normally distributed. For multiple comparisons and samples with unequal variances, two‐tailed one‐way ANOVA supplemented with Dunnett's *post hoc* test was used (SPSS version 24 IBM Statistics). Standard *P*‐value threshold of < 0.05 was used to indicate statistical significance. For the differential expression comparison, DESeq2 was used with the threshold *P*‐value set to 0.05 (Bioconductor). Pathway analysis was using Ingenuity Pathway Analysis software (IPA, Qiagen). Band intensities in WB and PAGE gels were analyzed using ImageJ software version 1.53c (National Institutes of Health, USA). MST statistics was done using MO. Affinity Analysis Software (NanoTemper). Flow cytometric analysis was done using FlowJo v10.1r5 software (FlowJo). The single molecules TIRF microscopy data were analyzed using bespoke MATLAB software to track and quantify the intensity of foci as a function of time where statistics of multiple images was calculated using two‐tailed Student's *t*‐test.

## Author contributions

**Larisa Chernyaeva:** Formal analysis; investigation; visualization; methodology; writing – original draft; writing – review and editing. **Giorgio Ratti:** Formal analysis; investigation; methodology; writing – original draft. **Laura Teirilä:** Formal analysis; investigation; methodology; writing – original draft. **Satoshi Fudo:** Investigation; methodology; writing – review and editing. **Uni Rankka:** Investigation; methodology. **Anssi Pelkonen:** Investigation; methodology; writing – review and editing. **Paula Korhonen:** Investigation; methodology; writing – review and editing. **Katarzyna Leskinen:** Software; investigation; methodology. **Salla Keskitalo:** Investigation; methodology. **Kari Salokas:** Software; methodology. **Christina Gkolfinopoulou:** Investigation; methodology. **Katrina E Crompton:** Investigation; methodology. **Matti Javanainen:** Software; investigation; methodology. **Lotta Happonen:** Software; investigation; methodology; writing – review and editing. **Markku Varjosalo:** Investigation; methodology. **Tarja Malm:** Resources. **Ville Leinonen:** Resources; investigation; writing – review and editing. **Angelika Chroni:** Resources; investigation; writing – review and editing. **Päivi Saavalainen:** Resources. **Seppo Meri:** Resources; writing – review and editing. **Tommi Kajander:** Investigation; visualization; methodology; writing – review and editing. **Adam JM Wollman:** Resources; software; investigation; visualization; methodology; writing – review and editing. **Eija Nissilä:** Investigation; visualization; methodology; writing – review and editing. **Karita Haapasalo:** Conceptualization; resources; supervision; funding acquisition; investigation; visualization; methodology; writing – original draft; writing – review and editing.

## Disclosure and competing interests statement

The authors declare that they have no conflict of interest.

## Supporting information



Appendix S1

Expanded View Figures PDF

Movie EV1

Movie EV2

Dataset EV1

Dataset EV2

Dataset EV3

PDF+

Source Data for Figure 1

Source Data for Figure 2

Source Data for Figure 3

Source Data for Figure 4

Source Data for Figure 5

Source Data for Figure 6

## Data Availability

mRNA data: Gene Expression Omnibus GSE193513 (https://www.ncbi.nlm.nih.gov/geo/query/acc.cgi?acc=GSE193513). Proteomic data: MassIVE MSV000088618 (https://massive.ucsd.edu/ProteoSAFe/dataset.jsp?task=595bf56584824151bb0b73e81f3caa73). MS crosslinking data: (http://massive.ucsd.edu/ProteoSAFe/status.jsp?task=40074352d93d41e8859034cc2f677bb0). Molecular dynamics data: (DOI:10.5281/zenodo.7533587. Link: https://doi.org/10.5281/zenodo.7533587).
