# Supplementary material for: Reduced binding of apoE4 to complement factor H promotes amyloid‐β oligomerization and neuroinflammation
Source: EMBO Rep. 2023 May 8;24(7):e56467. doi: 10.15252/embr.202256467 (PMC10328077; doi:10.15252/embr.202256467)
Supplement: Supplementary file 1 — Appendix S1 [file EMBR-24-e56467-s002.pdf]

Appendix Supporting Information: Table of contents

- **Appendix Fig. S1. Localization of apoE2/3/4 and FH in brain Aβ plaques.** Page 2
- **Appendix Fig. S2. Localization of apoE4 and FH in brain Aβ plaques.** Page 4
- **Appendix Fig. S3. Gating and histograms of U937 CR3 cells and SV40 microglia.** Page 5
- **Appendix Table S1. Patient data.** Page 6
- **Appendix Table S2. Quantification of binding parameters using dissociation constants (K_d) fit model of normalized (ΔFnorm) values in MST.** Page 7
- **Appendix Table S3. Band intensities in WBs and PAGE.** Pages 8-9
- **Appendix Table S4. Band intensities in WB.** Page 10
- **Appendix Table S5. Differential expression of transcripts in microglial cells upon stimulation with different combinations of apoE, Aβ1-42 and FH.** Pages 11-12

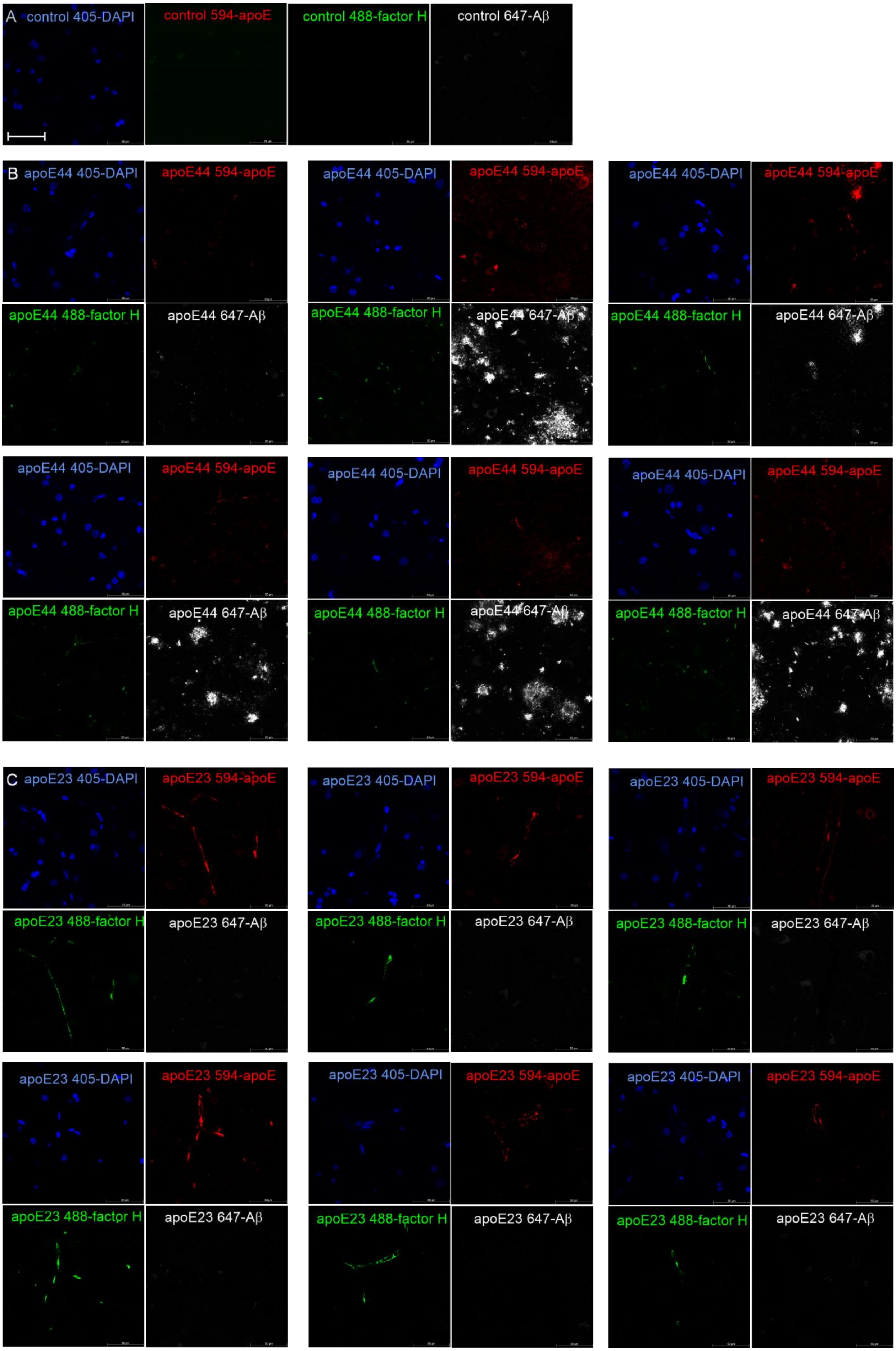
A control 405-DAPI
control 594-apoE
control 488-factor H
control 647-Aβ
B apoE44 405-DAPI
apoE44 594-apoE
apoE44 488-factor H
apoE44 647-Aβ
C apoE23 405-DAPI
apoE23 594-apoE
apoE23 488-factor H
apoE23 647-Aβ

Appendix Fig. S1. Localization of apoE2/3/4 and FH in brain Aβ plaques. Related to Fig. 1A-B. Immunofluorescence staining of

(A) control and six microscope images (size of the scale bar = 50 μM) from the

(B) apoE44 (n=2) and

(C) apoE23 (n=2) genotyped iNPH patient biopsy samples shown in separate channels. Staining of (green) FH, (red) apoE (white) Aβ plaques and (blue) nuclei are shown.

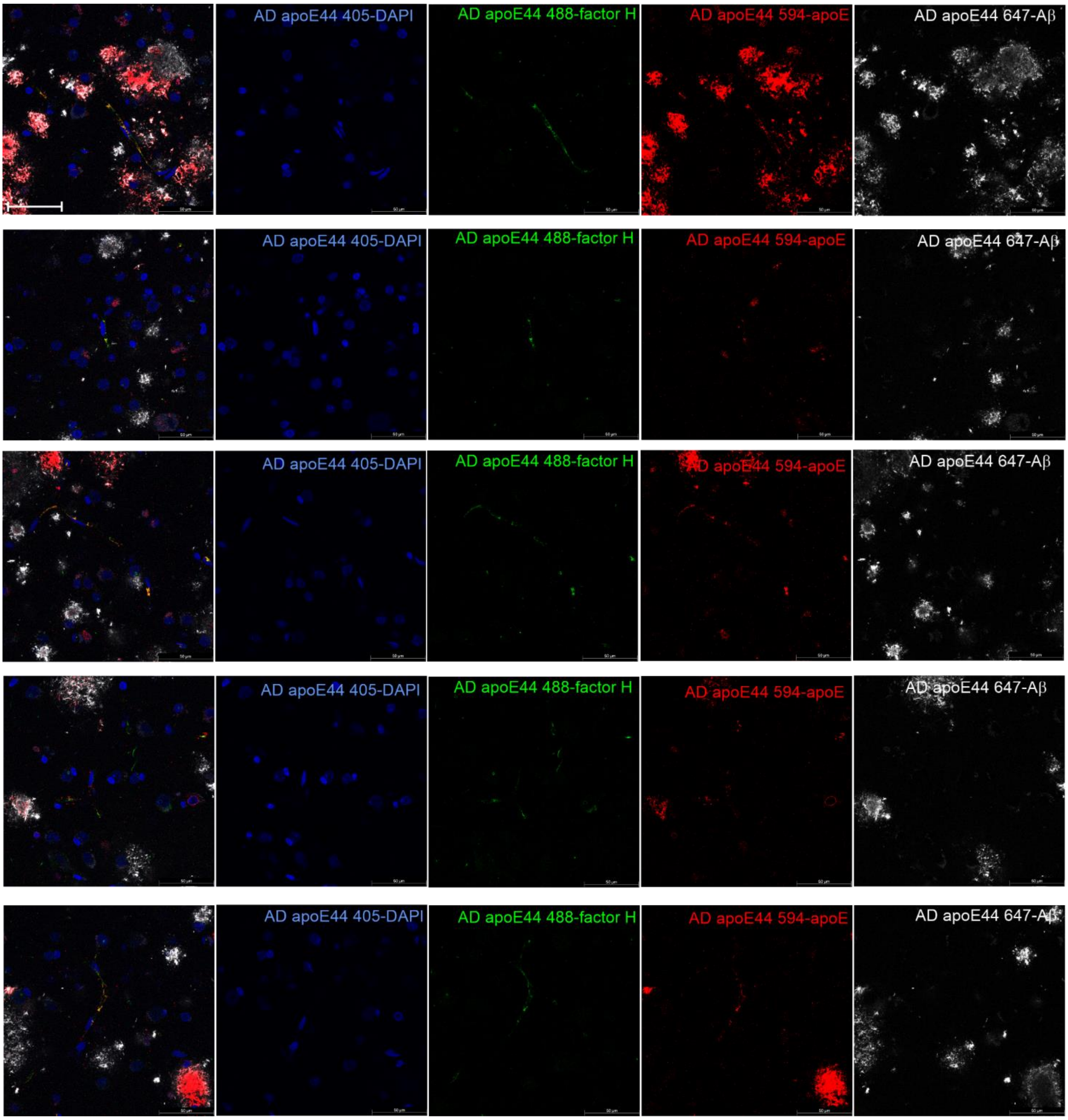

Appendix Fig. S2. Localization of apoE4 and FH in brain Aβ plaques. Related to Fig. 1C. Immunofluorescence staining of six microscope images shown in separate channels from a biopsy sample obtained from an iNPH patient diagnosed with Alzheimer's clinical syndrome (ACS) (size of the scale bar = 50 μM). Staining of (green) FH, (red) apoE (white) Aβ plaques and (blue) nuclei are shown.

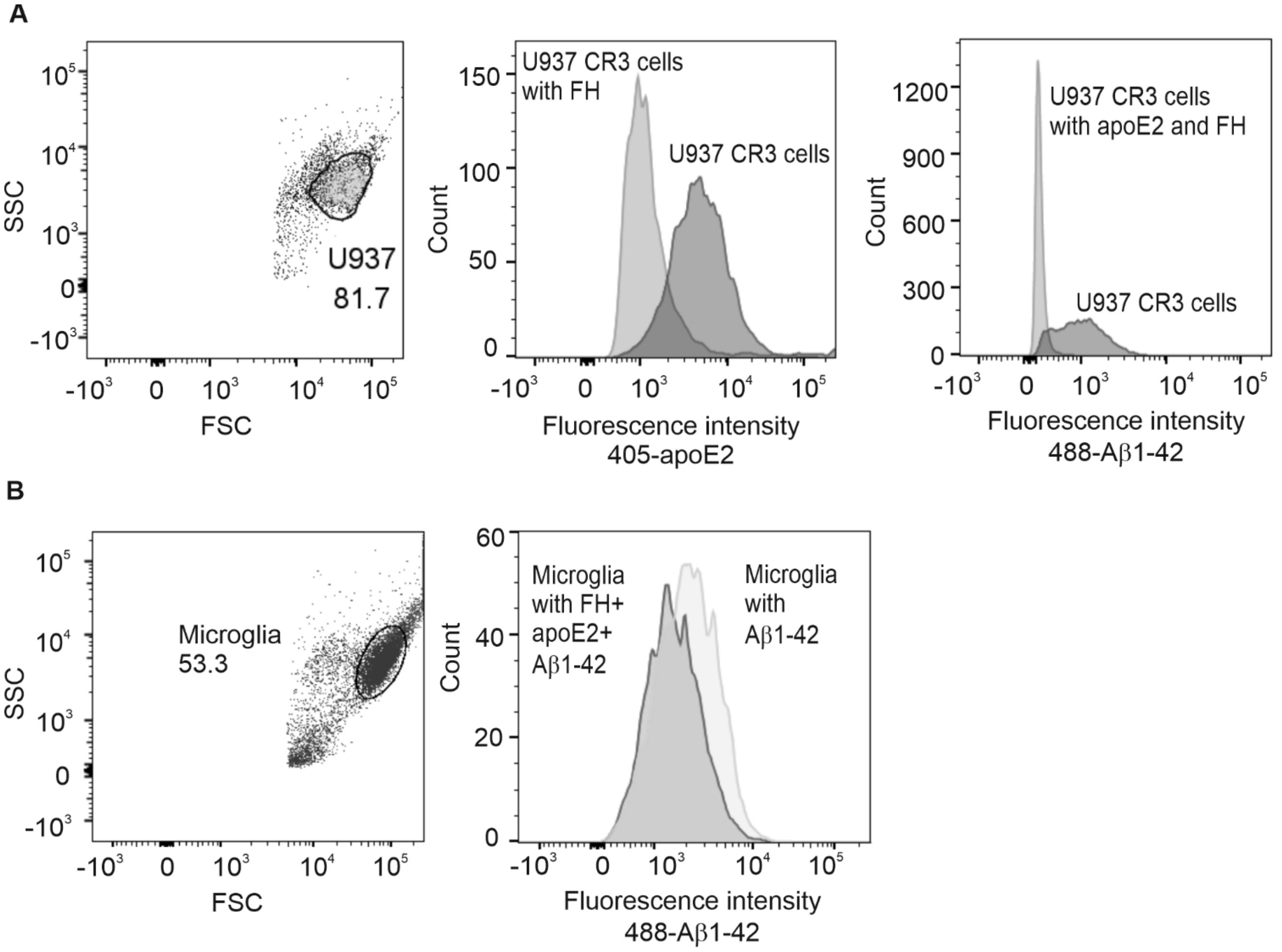

Appendix Fig. S3. Gating and histograms of U937 CR3 cells and SV40 microglia.

(A) Related to Fig. 3F-I. (left) Gating of Mn^{2+} activated U937 CR3 cells (81.7% of all cells in the gate). (Center) Inhibition of apoE binding by FH. (Right) Inhibition of Aβ binding by apoE2 and FH.

(B) Related to Fig. 5A (left) Gating of microglia SV40 cells (53.3% of all cells in the gate). (right) Inhibition of Aβ phagocytosis by apoE2 and FH. SSC, side scatter and FSC, forward scatter.

Appendix Table S1. Patient data

**NPH case number**	**age**	**sex**	***APOE* genotype**	**Aβ pathology**	**Tau**
1	82	M	34	yes*	no
2	79	M	23	no*	no
3	84	M	34	yes*	no
4	71	F	33	no*	no
5	59	M	33	yes*	no
6	72	M	33	yes*	no
7	80	M	44	yes*	no
8	66	M	33	yes*	no
9	58	F	33	no*	no
10	65	M	34	no*	no
11	87	M	33	yes*	no
12	79	M	33	no*	no
13	82	M	34	yes*	no
14	79	F	33	yes**	yes
15	77	F	33	yes*	no
16	72	F	44	yes**,***	yes
17	78	F	33	no*	no
18	71	M	23	no*	no
19	85	F	34	yes*	no
20	83	F	34	yes*	no
21	84	M	23	no*	no
22	70	F	34	no*	no
23	74	F	44	yes**	yes

Estimated Braak stage *1-4 or **5-6, *** Patient diagnosed for Alzheimer's clinical syndrome (ACS)

Appendix Table S2. Quantification of binding parameters using dissociation constants (K_d) fit model of normalized (ΔFnorm) values in MST (Related to Fig. 1I-K).

Ligand name	n	FH (nM)	K_d (µM)*	K_d Confidence	Reduced χ^2	Signal to Noise
native apoE2	3	2	0.002	0.0007		
native apoE4	3	2	0.3	0.2		
Ligand name	n	NT657-apoE2(1-165) (nM)	K_d (µM)*	K_d Confidence	Reduced χ^2	Signal to Noise
FH402H	3	2.8	0.496	0.139	1.8	16.3
FH402Y	3	2.8	0.253	0.96	1.7	10.2
FH1-4	3	2.8	0.999	0.598	2.1	9.6
FH19-20	3	2.8	no binding		3.1	0
Ligand name	n	NT647-FH5-7 402Y (nM)	K_d (µM)*	K_d Confidence	Reduced χ^2	Signal to Noise
apoE2	3	35	1.005	0.458	1.6	9.0
apoE3	3	35	1.384	0.553	3.1	11.0
apoE4	3	35	1.945	0.1162	0.7	8.1

*Reliable binding affinity was obtained in measurements with a signal to noise ratio > 5 and K_d confidence in the range of 68% using MO. Affinity Analysis Software. When a reliable binding curve could not be assigned, signal to noise ratio shows 0. Reduced χ^2 estimates the goodness of fit (1 = good fit).

Appendix Table S3. Band intensities in WBs and PAGE (Related to Fig. 2B)

SDS-PAGE and silver stain (Related to Fig. 2B, left)									
	description	apoE4/Aβ/FH (intensity)	apoE4/Aβ (intensity)	apoE2/Aβ/FH19-29 (intensity)	ApoE2/Aβ/FH (intensity)	apoE2/Aβ (intensity)	apoE2 (intensity)	Aβ/FH (intensity) *	Aβ (intensity) *
1	apoE/Aβ/FH or Aβ/FH complex	14868	0	0	18881	0	0	17819	0
2	apoE/Aβ complex 1	0	0	2165	0	408	0	0	0
3	apoE/Aβ complex 2	0	0	2795	0	908	0	0	0
4	apoE/Aβ complex 3	0	0	3318	0	889	0	0	0
5	apoE/Aβ complex 4	0	0	11519	0	4658	0	0	0
6	apoE/Aβ complex 5	0	0	15017	0	9765	0	0	0
7	apoE/Aβ complex 6	0	0	20758	2889	4746	0	0	0
8	apoE/Aβ complex 7/apoE	0	0	17614	5572	7229	2108	0	0
9	apoE/Aβ complex 8/apoE	5497	5076	112742	7516	9213	8474	0	0
10	Aβ	1611	3767	7351	3287	10644	0	6769	9527
WB (Related to Fig. 2B, middle)									
	description	apoE2/Aβ/FH19-20 (intensity)	apoE2/Aβ/FH (intensity)	apoE2/Aβ (intensity)	Aβ/FH (intensity)	Aβ (intensity)	Aβ monomer (intensity)	apoE4/Aβ/FH (intensity) *	apoE4/Aβ (intensity) *
1	Aβ aggregate	767	897	2872	16772	21369	0	0	282
2	apoE/Aβ/FH or Aβ/FH complex	0	16467	0	2089	0	0	0	0

3	apoE/Aβ complex 1	5347	0	425	0	0	0	0	0
4	apoE/Aβ complex 2	15842	0	9148	0	0	0	0	0
5	apoE/Aβ complex 3	18735	0	11294	0	0	0	0	0
6	apoE/Aβ complex 4	18932	0	12551	0	0	0	0	0
7	apoE/Aβ complex 5	17650	0	14099	0	0	0	0	0
8	apoE/Aβ complex 6	15514	3667	13637	0	0	0	0	0
9	apoE/Aβ complex 7	23919	5862	10544	0	0	0	0	0
10	apoE/Aβ complex 8	18682	3166	7645	0	0	0	18118	20733
11	Aβ	7381	8376	10658	3639	6536	12186	18775	15802

WB (Related to Fig. 2B right)							
	description	apoE2/Aβ (intensity)	apoE3/Aβ (intensity)	apoE4/Aβ (intensity)	apoE2/Aβ/FH (intensity)	apoE3/Aβ/FH (intensity)	apoE4/Aβ/FH (intensity)
1	apoE/Aβ/FH complex	0	0	0	33129	3825	0
2	apoE/Aβ complex 1	32125	0	0	0	0	0
3	apoE/Aβ complex 2	36240	1612	0	0	0	0
4	apoE/Aβ complex 3	48769	27212	0	41821	10717	0
5	apoE/Aβ complex 4	31930	40914	0	46849	36685	0
6	apoE/Aβ complex 4	38548	71915	43404	52189	58330	57632
7	Aβ monomer	37307	52884	52884	52694	48099	57091

*not shown in the gel image

Appendix Table S4. Band intensities in WB (Related to Fig. 4E)

		C3 (intensity)	C3b (intensity)	iC3b (intensity)	Aβ/BSA (intensity)	Aβ (intensity)	Aβ/FH (intensity)	E2/Aβ (intensity)	E2/Aβ /FH (intensity)	E4/Aβ (intensity)	E4/Aβ /FH (intensity)
1	C3α and C3bα	16126	14434	3049	13526	19537	13665	17663	12098	16637	15262
2	C3β	21402	7478	24132	6985	13315	13265	13856	8094	10099	13884
3	iC3bα	0	0	27006	0	0	13309	1572	5116	0	10140

Appendix Table S5. Differential expression of transcripts in microglial cells upon stimulation with different combinations of apoE, Aβ1-42 and FH (Related to Table 1 in the manuscript).

Gene name (alternative name)	Gene name extended/encoded protein	Description	Cited in
Upregulation with FH and apoE2			
CIAPIN (Anamorsin)	Cytokine induced apoptosis inhibitor	CIAPIN has been suggested to inhibit neuronal apoptotic cell death.	(Yun *et al*, 2014)
GPR176	G protein coupled receptor 176	No known association of this particular GPR. Several studies have suggested that GPRs play a role in AD pathogenesis.	(Thathiah & De Strooper, 2011)
TRIM11	Tripartite motif containing 11	TRIM11 can suppress induced neurotoxic effects due to its interaction with, humanin, a neuroprotective peptide.	(Niikura *et al*, 2003)
MADD	MAP kinase activating death domain	MADD expression has been found to be inhibited specifically in hippocampal neurons in AD; this is associated with an increased neuronal cell death. Some mutations of MADD are linked with AD onset.	(Del Villar & Miller, 2004; Saad *et al*, 2015)
HGS (Hrs)	Hepatocyte growth factor-regulated tyrosine kinase substrate	HGS has been found to be significantly decreased in AD brains.	(Gireud-Goss *et al*, 2020)
NISCH	Nischarin	Correlates negatively with the presence of apoE4 disease allele in this study	
CES2	Carboxylesterase 2	CES2 may play a role in protecting the central nervous system from toxic esters. Perhaps a component of the BBB system.	(Zhang *et al*, 2002)
ITSN2 and ITSN2	Intersectin 1 and Intersectin 2	ITSN1 and ITSN2 are found to be one of the most expressed genes in AD.	(Herrero-Garcia & O'Bryan, 2017)
FHL2	Four and a half LIM domains 2	Interacts with presenilin 2 that has a role in APP processing.	(Tanahashi & Tabira, 2000)
FBN1	Fibrillin-1	FBN1 has a role in supporting the BBB.	(Van der Donckt *et al*, 2015)
PLXNA1	Plexin A1	Correlates negatively with Aβ pathology.	(Huang *et al*, 2021)
PTPRF	Protein Tyrosine Phosphatase Receptor Type F	Is involved in cell adhesion pathway and correlates negatively with Aβ pathology and with the presence of apoE4 allele in this study.	(Huang *et al.*, 2021; Jiang *et al*, 2021)
RBBP7	Histone-binding protein RBBP7	Correlates negatively with Aβ pathology.	(Huang *et al.*, 2021)

Upregulation with FH and apoE4			
ADNP	Activity dependent neuroprotector homeobox	ADNP is found to be significantly decreased in the serum of AD patients.	(Gozes, 2015)
RAP2B	Member of RAS oncogene family	A significant AD associated SNP is located near RAP2B on chromosome 3.	(Yan *et al*, 2021)
PAFAH1B2	Platelet activating factor acetylhydrolase α	PAFAH1B2, if less expressed, decreases Aβ formation helping its degradation at its previous stages.	(Page *et al*, 2012)
ERO1A	Endoplasmic reticulum oxidoreductase 1 alpha	No directly known association. The role of ERO1A is implied in promoting calcium induced apoptosis.	(Seervi *et al*, 2013)
LARP1	La-related protein 1	LARP1 associates mTOR complex and can be functionally linked to AD.	(Wang *et al*, 2015)
WNT5A	Wnt family member 5A	Abnormal expression of WNT5A is linked with inflammation and cytotoxicity promoted by Aβ in neurons.	(Li *et al*, 2011)
FOXP1	Forkhead box protein P1	No directly known association.	
PDK1	Pyruvate dehydrogenase kinase 1	PDK1 is linked with toxicity induced by Aβ since it decreases α-secretase activity and promotes AD disease progression.	(Checler, 2013; Pietri *et al*, 2013)
FOXO3	Forkhead box O3	FOXO3 is involved in Aβ induced neuronal death, and in production of dysfunctional Aβ.	(Shi *et al*, 2016)
DAAM1	Dishevelled Associated Activator Of Morphogenesis 1	Correlates positively with Aβ pathology.	(Huang *et al.*, 2021)
PGBD1	PiggyBac Transposable Element Derived 1	Correlates positively with Aβ pathology.	(Huang *et al.*, 2021)
TRA2A	Transformer 2 Alpha Homolog	Correlates positively with the presence of apoE4 disease allele in this study	

References

Checler F (2013) Alzheimer's and prion diseases: PDK1 at the crossroads. *Nat Med* 19: 1088-1090

Del Villar K, Miller CA (2004) Down-regulation of DENN/MADD, a TNF receptor binding protein, correlates with neuronal cell death in Alzheimer's disease brain and hippocampal neurons. *Proc Natl Acad Sci U S A* 101: 4210-4215

Gireud-Goss M, Reyes S, Tewari R, Patrizz A, Howe MD, Kofler J, Waxham MN, McCullough LD, Bean AJ (2020) The ubiquitin ligase UBE4B regulates amyloid precursor protein ubiquitination, endosomal trafficking, and amyloid beta42 generation and secretion. *Mol Cell Neurosci* 108: 103542

Gozes I (2015) Activity-dependent neuroprotective protein (ADNP): from autism to Alzheimer's disease. *Springerplus* 4: L37

Herrero-Garcia E, O'Bryan JP (2017) Intersectin scaffold proteins and their role in cell signaling and endocytosis. *Bba-Mol Cell Res* 1864: 23-30

Huang W, Bartosch AM, Xiao H, Maji S, Youth EHH, Flowers X, Leskinen S, Tomljanovic Z, Iodice G, Boyett D *et al* (2021) An immune response characterizes early Alzheimer's disease pathology and subjective cognitive impairment in hydrocephalus biopsies. *Nat Commun* 12: 5659

Jiang F, Liu H, Peng F, Liu Z, Ding K, Song J, Li L, Chen J, Shao Q, Yan S *et al* (2021) Complement C3a activates osteoclasts by regulating the PI3K/PDK1/SGK3 pathway in patients with multiple myeloma. *Cancer Biol Med*

Li B, Zhong L, Yang X, Andersson T, Huang M, Tang SJ (2011) WNT5A signaling contributes to Abeta-induced neuroinflammation and neurotoxicity. *PLoS One* 6: e22920

Niikura T, Hashimoto Y, Tajima H, Ishizaka M, Yamagishi Y, Kawasumi M, Nawa M, Terashita K, Aiso S, Nishimoto I (2003) A tripartite motif protein TRIM11 binds and destabilizes Humanin, a neuroprotective peptide against Alzheimer's disease-relevant insults. *Eur J Neurosci* 17: 1150-1158

Page RM, Munch A, Horn T, Kuhn PH, Colombo A, Reiner O, Boutros M, Steiner H, Lichtenthaler SF, Haass C (2012) Loss of PAFAH1B2 reduces amyloid-beta generation by promoting the degradation of amyloid precursor protein C-terminal fragments. *J Neurosci* 32: 18204-18214

Pietri M, Dakowski C, Hannaoui S, Alleaume-Butaux A, Hernandez-Rapp J, Ragagnin A, Mouillet-Richard S, Haik S, Bailly Y, Peyrin JM *et al* (2013) PDK1 decreases TACE-mediated alpha-secretase activity and promotes disease progression in prion and Alzheimer's diseases. *Nat Med* 19: 1124-1131

Saad M, Brkanac Z, Wijsman EM (2015) Family-based genome scan for age at onset of late-onset Alzheimer's disease in whole exome sequencing data. *Genes Brain Behav* 14: 607-617

Seervi M, Sobhan PK, Joseph J, Ann Mathew K, Santhoshkumar TR (2013) ERO1alpha-dependent endoplasmic reticulum-mitochondrial calcium flux contributes to ER stress and mitochondrial permeabilization by procaspase-activating compound-1 (PAC-1). *Cell Death Dis* 4: e968

Shi C, Viccaro K, Lee HG, Shah K (2016) Cdk5-Foxo3 axis: initially neuroprotective, eventually neurodegenerative in Alzheimer's disease models. *J Cell Sci* 129: 1815-1830

Tanahashi H, Tabira T (2000) Alzheimer's disease-associated presenilin 2 interacts with DRAL, an LIM-domain protein. *Hum Mol Genet* 9: 2281-2289

Thathiah A, De Strooper B (2011) The role of G protein-coupled receptors in the pathology of Alzheimer's disease. *Nat Rev Neurosci* 12: 73-87

Van der Donckt C, Roth L, Vanhoutte G, Blockx I, Bink DI, Ritz K, Pintelon I, Timmermans JP, Bauters D, Martinet W *et al* (2015) Fibrillin-1 impairment enhances blood-brain barrier permeability and xanthoma formation in brains of apolipoprotein E-deficient mice. *Neuroscience* 295: 11-22

Wang W, Mandel J, Bouaziz J, Commenges D, Nabirotchkine S, Chumakov I, Cohen D, Guedj M, Alzheimer's Disease Neuroimaging I (2015) A Multi-Marker Genetic Association Test Based on the Rasch Model Applied to Alzheimer's Disease. *PLoS One* 10: e0138223

Yan Q, Nho K, Del-Aguila JL, Wang X, Risacher SL, Fan KH, Snitz BE, Aizenstein HJ, Mathis CA, Lopez OL *et al* (2021) Genome-wide association study of brain amyloid deposition as measured by Pittsburgh Compound-B (PiB)-PET imaging. *Mol Psychiatry* 26: 309-321

Yun N, Lee YM, Kim C, Shibayama H, Tanimura A, Hamanaka Y, Kanakura Y, Park IS, Jo A, Shin JH *et al* (2014) Anamorsin, a novel caspase-3 substrate in neurodegeneration. *J Biol Chem* 289: 22183-22195

Zhang W, Xu G, McLeod HL (2002) Comprehensive evaluation of carboxylesterase-2 expression in normal human tissues using tissue array analysis. *Appl Immunohistochem Mol Morphol* 10: 374-380
