# Supplementary material for: Reduced binding of apoE4 to complement factor H promotes amyloid‐β oligomerization and neuroinflammation
Source: EMBO Rep. 2023 May 8;24(7):e56467. doi: 10.15252/embr.202256467 (PMC10328077; doi:10.15252/embr.202256467)
Supplement: Supplementary file 2 — Expanded View Figures PDF [file EMBR-24-e56467-s009.pdf]

Expanded View Figures

Figure EV1. Localization of apoE and FH in brain Aβ plaques. Related to Fig 1.

A, B Immunofluorescence staining of apoE33 and apoE34 geotyped iNPH patient biopsy samples showing (A) (arrow) colocalization of (green) apoE, (red) Aβ and (purple) FH in the brain, (B) colocalization of (green) apoE and (purple) FH around and away from brain capillaries surrounded by (red) Aβ plaques. The areas of colocalization are shown in the bright field images. Scale bar = 50 µm.

C Colocalization of (green) apoE, and (purple) FH around brain capillaries in the vicinity of (red) Aβ plaques. Scale bar = 50 µm.

D Colocalization of (green) apoE and (red) FH around cells covering capillaries in the brain, indicating protection of the blood–brain barrier (BBB) and endothelial cells from complement attack. The blue nuclei were detected using DAPI staining. Scale bar = 50 µm.

E Colocalization of (green) apoE in the (purple) Aβ plaque core. Scale bar = 50 µm.

F A dense (green) amyloid plaque surrounded by cells showing localization of (red) FH around the cells and the Aβ core. (arrow) Localization of FH is shown.

G Related to Fig 1E. (left) Silver staining of a loading control showing the major apoA1 band of HDL and (right) apoE WB of HDL particles isolated from apoE genotyped iNPH patient plasma. HDL samples (5–20 µl based on protein concentration) were incubated with 1 × Bolt™ LDS Sample Buffer and 1 × Bolt™ Sample Reducing Agent (Thermo Fisher) for 10 min at 95°C and run on gel (Mini-PROTEAN TGX Stain-Free precast gels 4–20%, Bio-Rad), 60 min, 120 V in TGS buffer (Bio-Rad). (left) The gel was imaged with GelDoc system (Bio-Rad). (right) WB was performed using 1:5,000 polyclonal antibody against purified human apoE (kind gift from Dr. Matti Jauhiainen) and IRDyeÛ 680RD labeled anti-Rabbit IgG (Cat#926-68073, LI-COR). (left) The major protein, ApoAI, at 25 kDa was found at similar levels in the samples. (right) The 37 kDa band of apoE is indicated.

 © 2023 The Authors

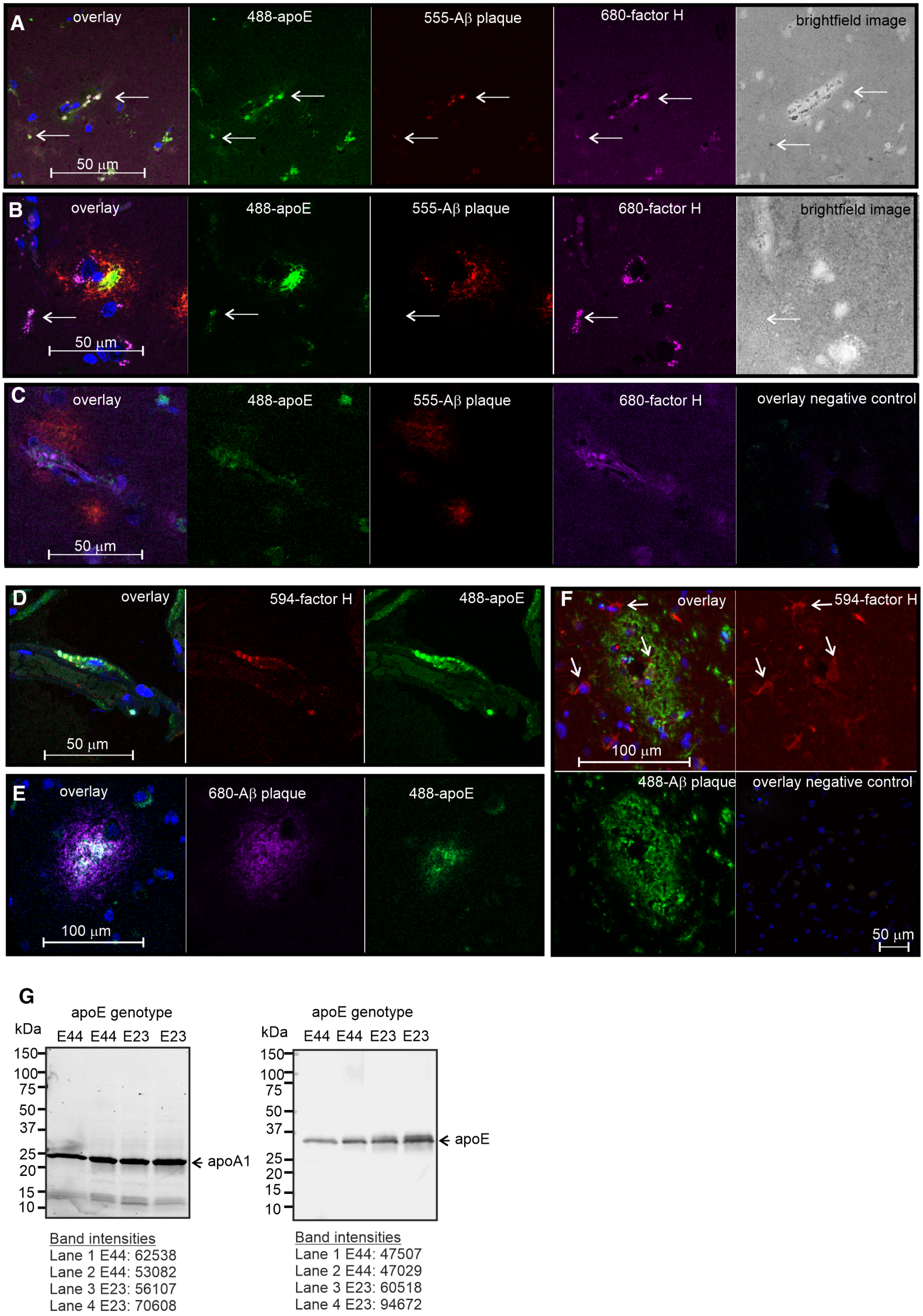
A
overlay
488-apoE
555-Aβ plaque
680-factor H
brightfield image
50 μm
B
overlay
488-apoE
555-Aβ plaque
680-factor H
brightfield image
50 μm
C
overlay
488-apoE
555-Aβ plaque
680-factor H
overlay negative control
50 μm
D
overlay
594-factor H
488-apoE
50 μm
E
overlay
680-Aβ plaque
488-apoE
100 μm
F
overlay
594-factor H
100 μm
488-Aβ plaque
overlay negative control
50 μm
G
apoE genotype
kDa
E44 E44 E23 E23
150
100
75
50
37
25
20
15
10
apoA1
Band intensities
Lane 1 E44: 62538
Lane 2 E44: 53082
Lane 3 E23: 56107
Lane 4 E23: 70608
apoE genotype
kDa
E44 E44 E23 E23
apoE
Band intensities
Lane 1 E44: 47507
Lane 2 E44: 47029
Lane 3 E23: 60518
Lane 4 E23: 94672

Figure EV1.

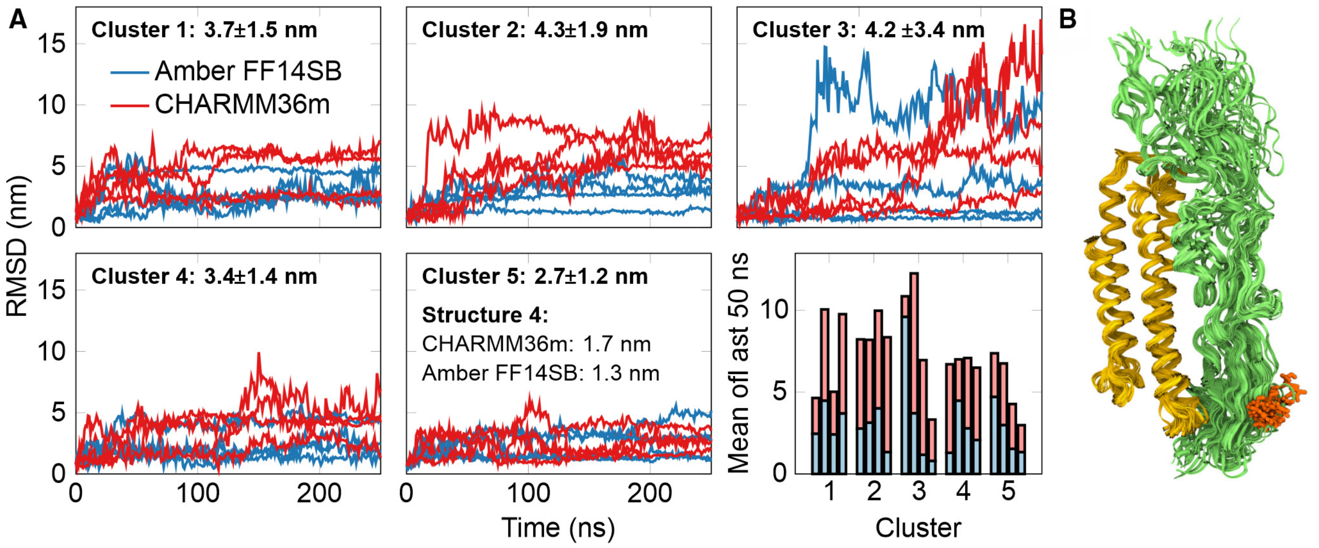

Figure EV2. The trajectories of atomistic MD simulations for the different HADDOCK-predicted structures. Related to Fig 1L.

A The root mean square deviation (RMSD) is calculated for FH5-7 after RMSD-fitting apoE2 to its original conformation. Input data included five clusters each with four structures. MD Data for the two force fields are shown with different colors, and the numbers show the mean RMSD value and its standard deviation for each cluster. The bottom right panel shows the average values of the RMSD curves during the last 50 ns of simulation with the same coloring. Many initial structures were not stable, and the dimer dissociated rapidly during the simulation. Still, we observed that cluster 5 was on average the most stable, and no outliers were observed with either force field. Structure number 4 in this cluster had the smallest total RMSD value from simulations of the two force fields (values provided in the insert) and was thus chosen for further analysis on hydrogen bonds formed at the dimer interface.

B The best MD ensemble structure with residue 402 shown in red, pointing away from the protein interface between apoE2 (yellow) and FH5-7 (green).

 © 2023 The Authors

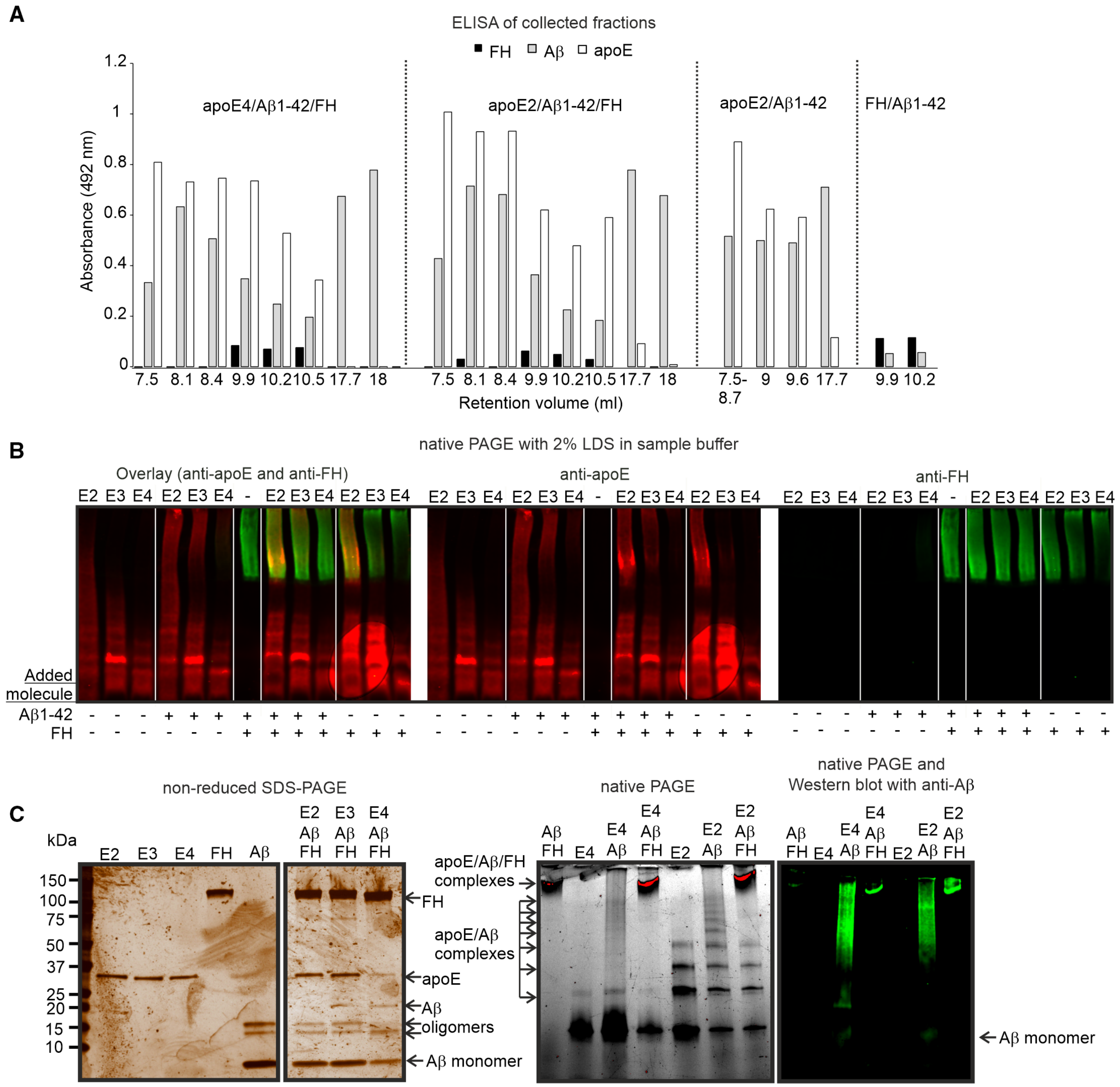
A
ELISA of collected fractions
FH Aβ apoE
Absorbance (492 nm)
apoE4/Aβ1-42/FH
apoE2/Aβ1-42/FH
apoE2/Aβ1-42
FH/Aβ1-42
Retention volume (ml)
B
native PAGE with 2% LDS in sample buffer
Overlay (anti-apoE and anti-FH)
anti-apoE
anti-FH
Added molecule
Aβ1-42
FH
C
non-reduced SDS-PAGE
native PAGE
native PAGE and Western blot with anti-Aβ
kDa
apoE/Aβ/FH complexes
FH
apoE/Aβ complexes
apoE
Aβ oligomers
Aβ monomer

Figure EV3.

◀ **Figure EV3. Analyzing FH, apoE and Aβ1-42 complexes.**

A ELISA of collected fractions at different retention volumes (x-axis) from size exclusion chromatography of apoE and 488-Aβ1-42-incubated samples with and without FH shown in Fig 2A. The collected fractions were diluted 1:2 in sodium bicarbonate buffer, pH 9.6 and coated in 96-well maxisorp plates. The wells were blocked with 3% fatty acid-free BSA in PBS for 2 h and after one wash with PBS, incubated with 1:3,000 goat anti-FH (Calbiochem), 1:3,000 rabbit polyclonal antibody against purified human apoE (from Dr. Matti Jauhiainen) or 1:1,000 rabbit anti-Aβ (Invitrogen) antibodies in 0.3% BSA in PBS for 1 h at 37°C. The wells were washed three times with PBS and incubated with 1:10,000 diluted HRP-conjugated anti-goat or anti-rabbit IgG antibodies (Jackson Immunoresearch) for 45 min at 37°C. After four washes with PBS, the OPD substrate was added in the wells, and the reaction was stopped after sufficient development of color with 3 M H_2SO_4 solution. The absorbance was measured at 492 nm.

B Related to Fig 2B. After 72 h incubation the samples were run on gels to conduct (above) native PAGE with Bolt LDS Sample Buffer (Thermo Fisher Scientific) and western blot of the samples using rabbit anti-apoE and goat anti-FH antibodies followed with dual labeling using IRDyeÛ 680RD Donkey anti-Rabbit IgG (Cat# 926-68073, LI-COR) and IRDyeÛ800CW labeled anti-Goat IgG (Cat# 926-32213, LI-COR) as described in Materials and Methods. Colocalization of FH with apoE2 and apoE3 (apoE2 > apoE3) but not with apoE4 can be detected in the presence and absence of Aβ1-42.

C Aβ1-42 and FH were all separated in (left) SDS–PAGE, while clear complexes were detected in (right) native PAGE. The presence of Aβ1-42 in native PAGE was detected by WB using anti-Aβ antibody. Loss of Aβ1-42 monomer in the presence of FH and low intensity of Aβ1-42 in complex with FH indicates minimal interaction between FH and Aβ1-42 and aggregation of Aβ1-42 in the absence of apoE. The intensity of apoE/Aβ/FH complexes indicates higher affinity of FH to apoE2/Aβ and then to apoE4/Aβ.

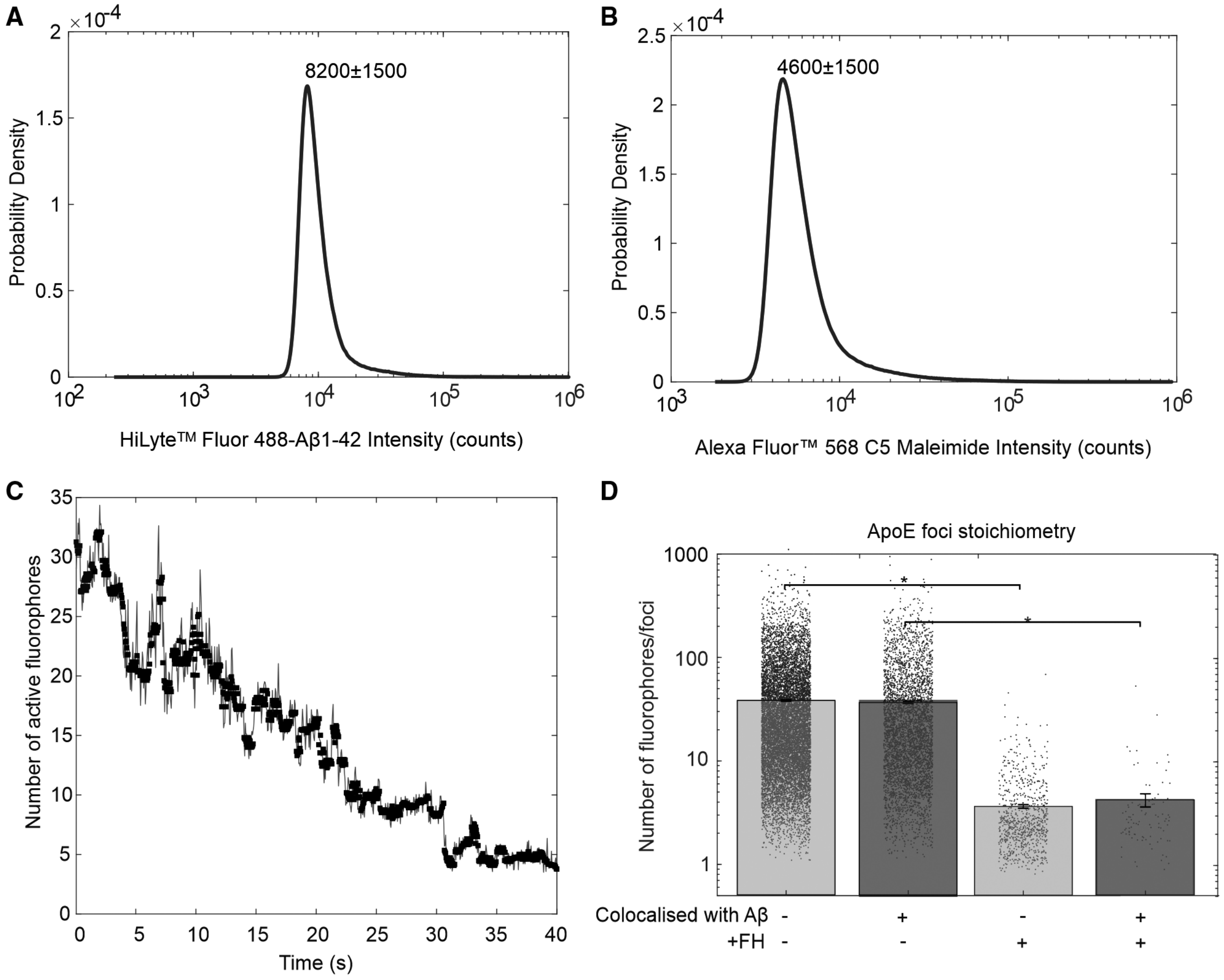

Figure EV4.

 © 2023 The Authors

Figure EV4. Single-molecule characterization of apoE and Aβ1-42 complexes. Related to Fig 2C–E.

A, B Characteristic intensity distribution of single Hylite 488 and AlexaFluor 568 molecules.

C Step-wise photobleach trace of Aβ1-42 oligomer foci. Raw intensity data plotted as line with Chung-Kennedy filtered intensity overlaid as squares (Chung & Kennedy, 1991).

D Jitter plot of apoE2 foci stoichiometry in number of fluorophores colocalized or not with Aβ1-42 and in the presence and absence of FH. Statistics (* $P < 0.05$) was calculated using Student's *t*-test from each colocalized foci in multiple images ($n = 12$) in each sample. Error bars indicate SD values.

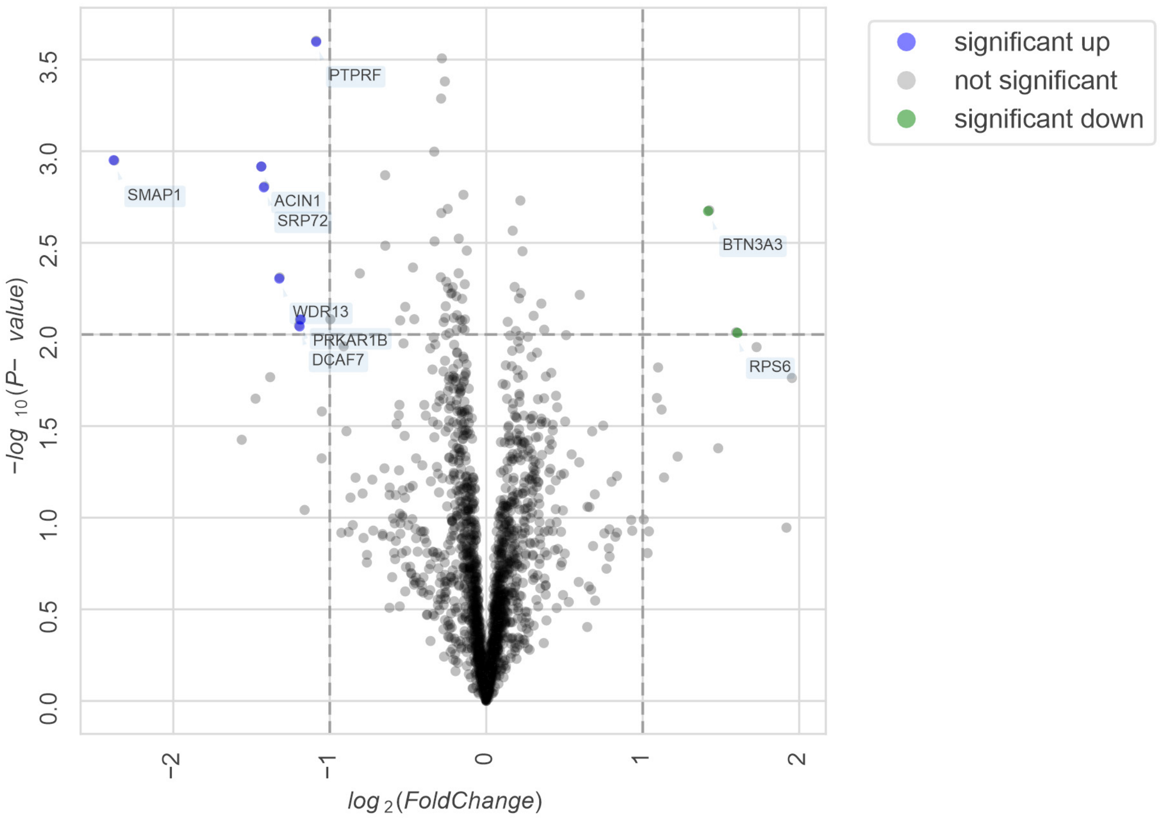

Figure EV5. Volcano plot of differentially expressed proteins. Related to Fig 6A.

The (x-axis) log2 fold change represents the difference between the levels of expression for each protein while (y-axis) −log10 (*P*-values) represents significance of each protein. Proteins with statistical significance and ± 1 log2 fold change are shown.

© 2023 The Authors
