# Supplementary figures and images for: Reduced binding of apoE4 to complement factor H promotes amyloid‐β oligomerization and neuroinflammation
Source: EMBO Rep. 2023 May 8;24(7):e56467. doi: 10.15252/embr.202256467 (PMC10328077; doi:10.15252/embr.202256467)

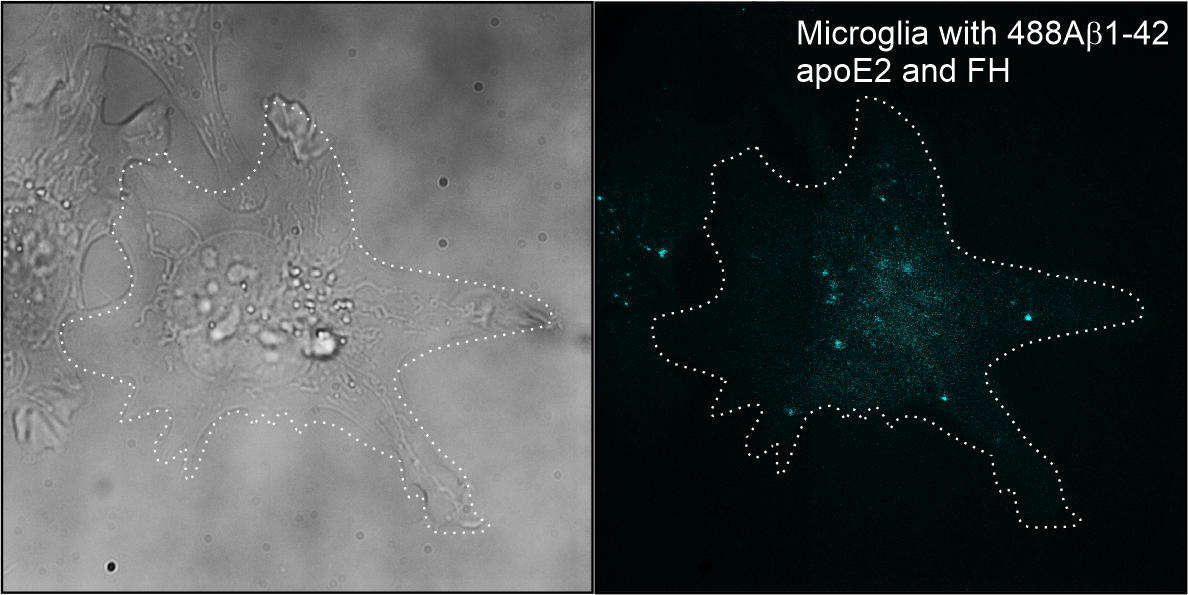

Supplement: Supplementary file 3 — Movie EV1 [file EMBR-24-e56467-s004.zip › Movie EV1/Movie EV1.tif]

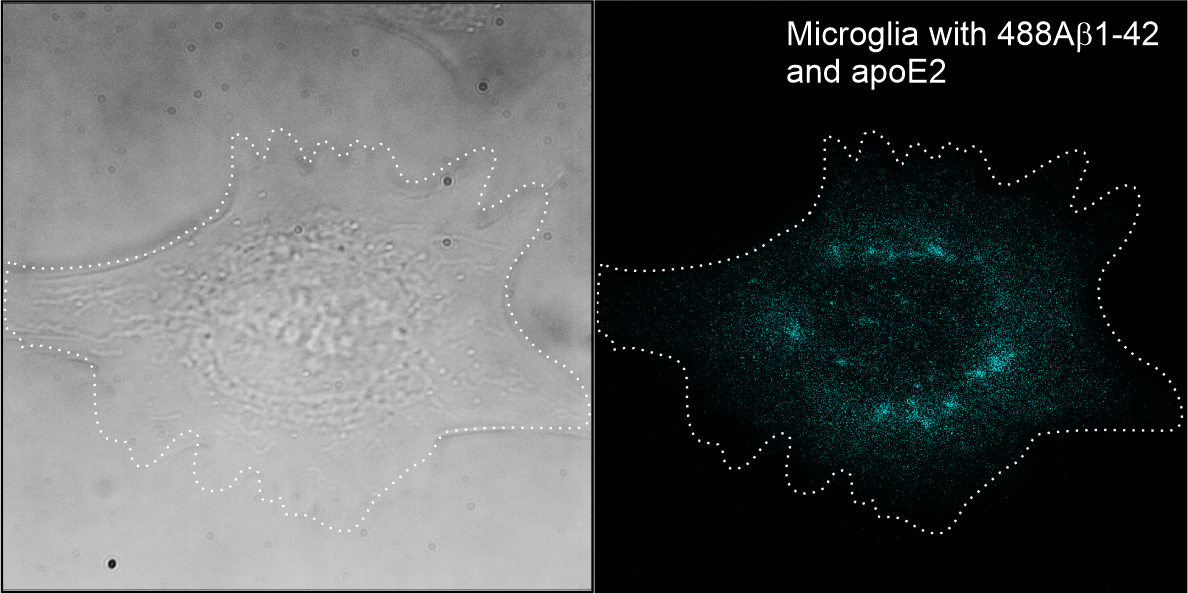

Supplement: Supplementary file 4 — Movie EV2 [file EMBR-24-e56467-s011.zip › Movie EV2/Movie EV2.tif]

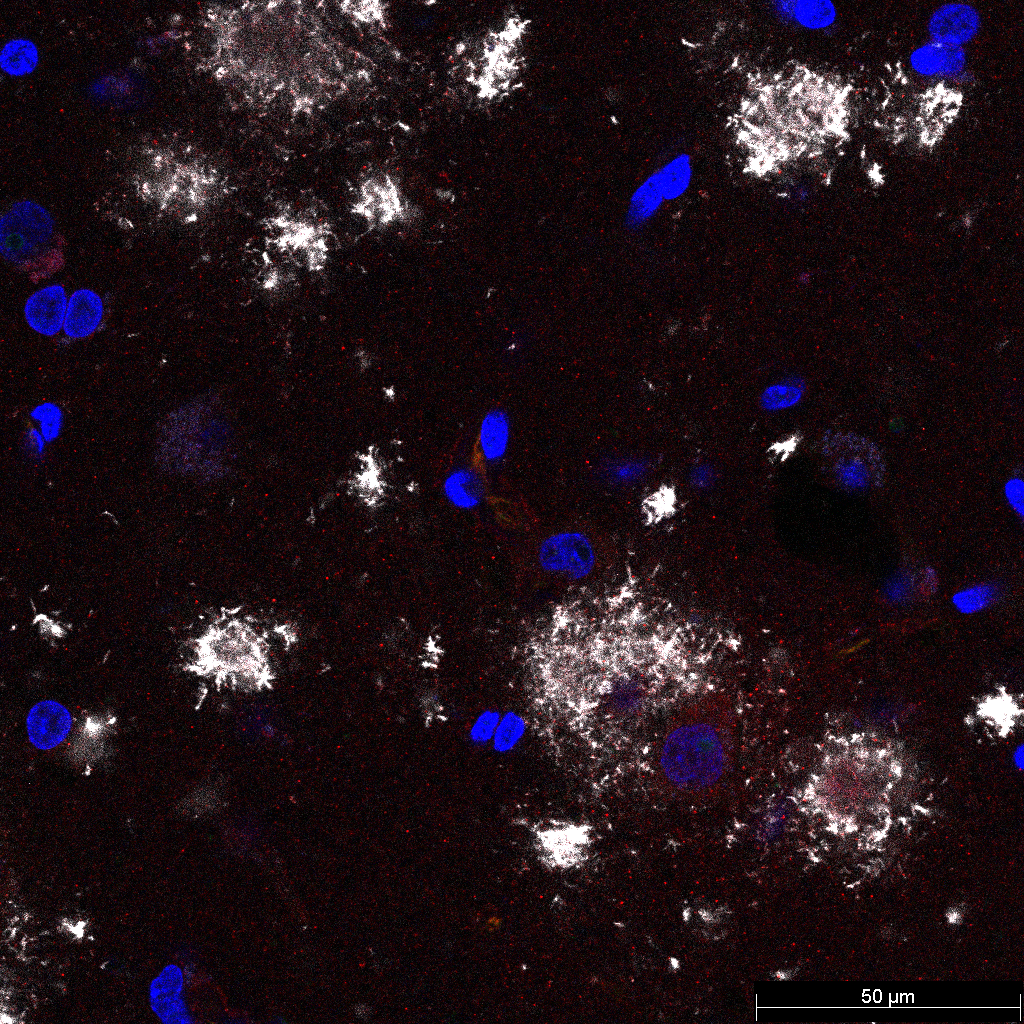

Supplement: Supplementary file 9 — Source Data for Figure 1 [file EMBR-24-e56467-s014.zip › Fig. 1 Source data/Fig 1A-C IF/Fig 1A apoE44 P1 Left.tif]

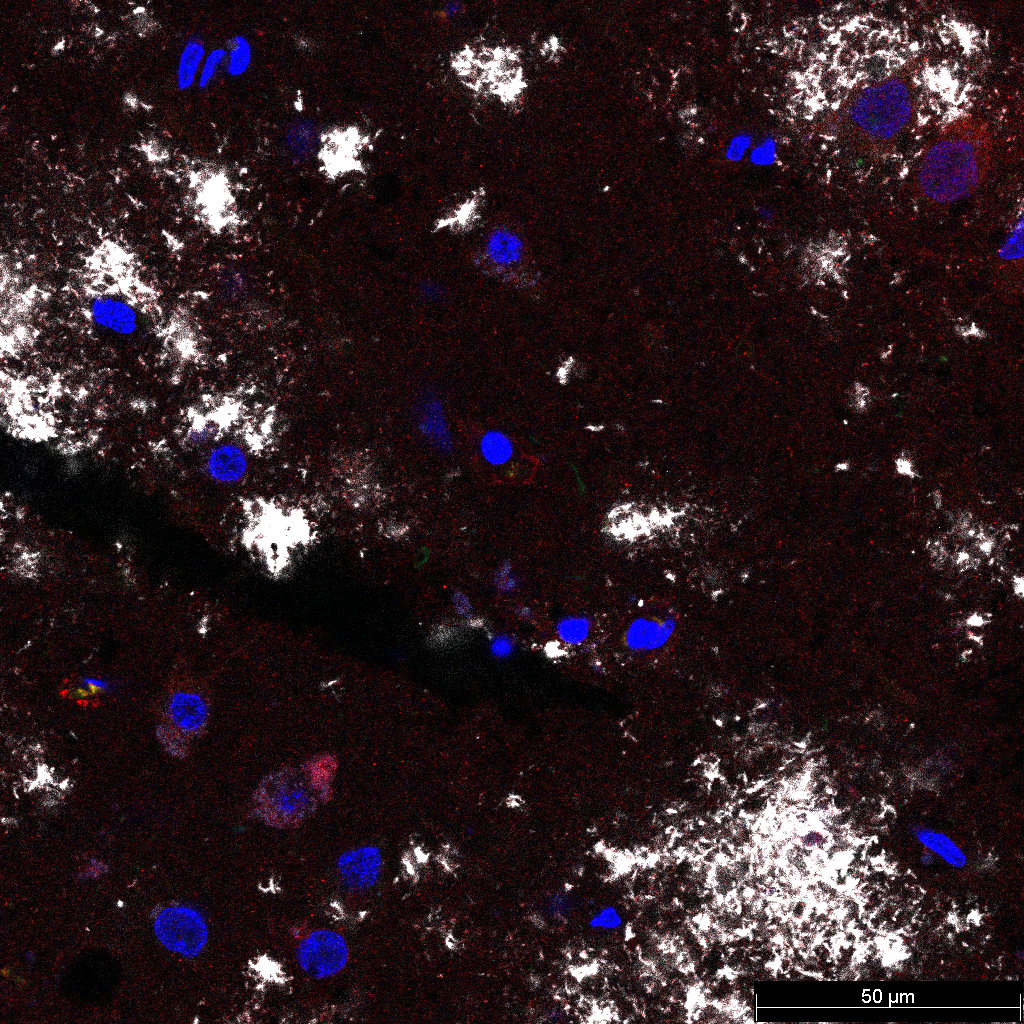

Supplement: Supplementary file 9 — Source Data for Figure 1 [file EMBR-24-e56467-s014.zip › Fig. 1 Source data/Fig 1A-C IF/Fig 1A apoE44 P1 Right.tif]

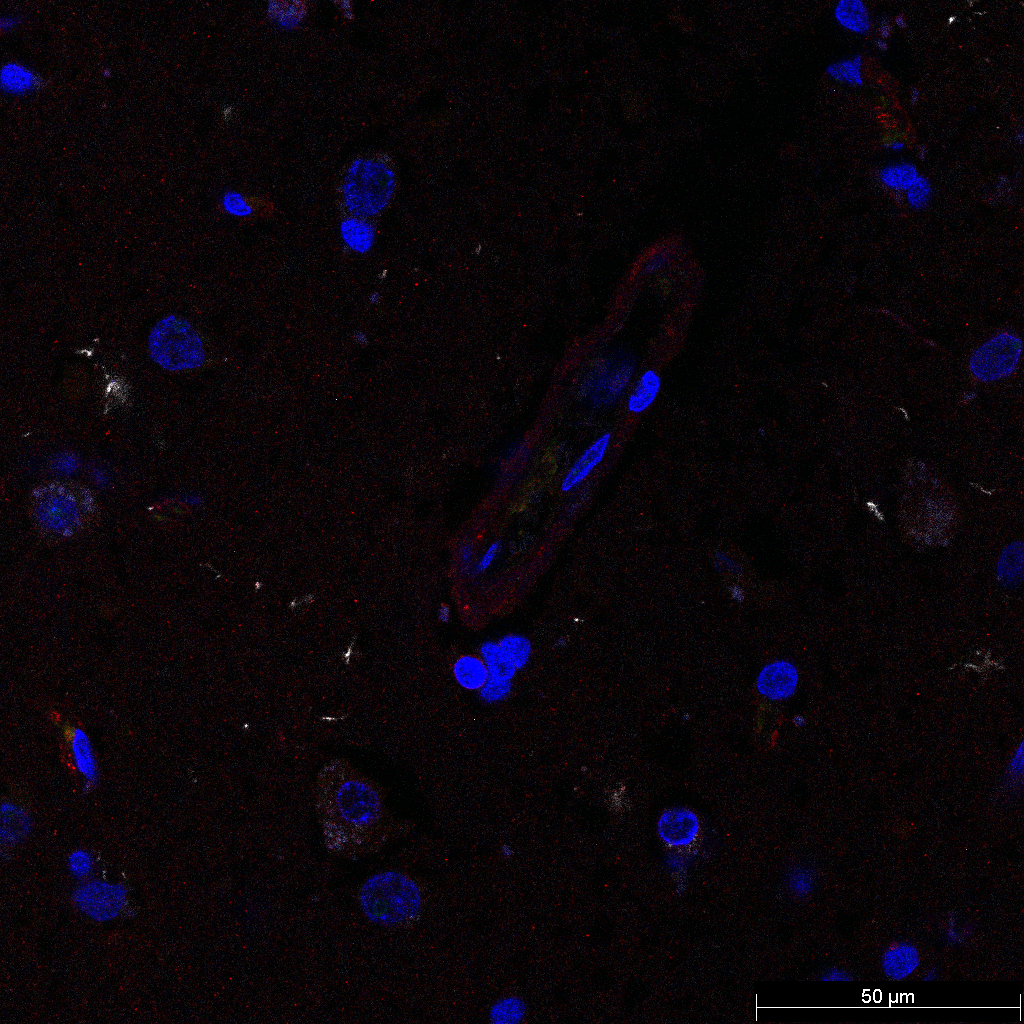

Supplement: Supplementary file 9 — Source Data for Figure 1 [file EMBR-24-e56467-s014.zip › Fig. 1 Source data/Fig 1A-C IF/Fig 1A apoE44 P2 Left.tif]

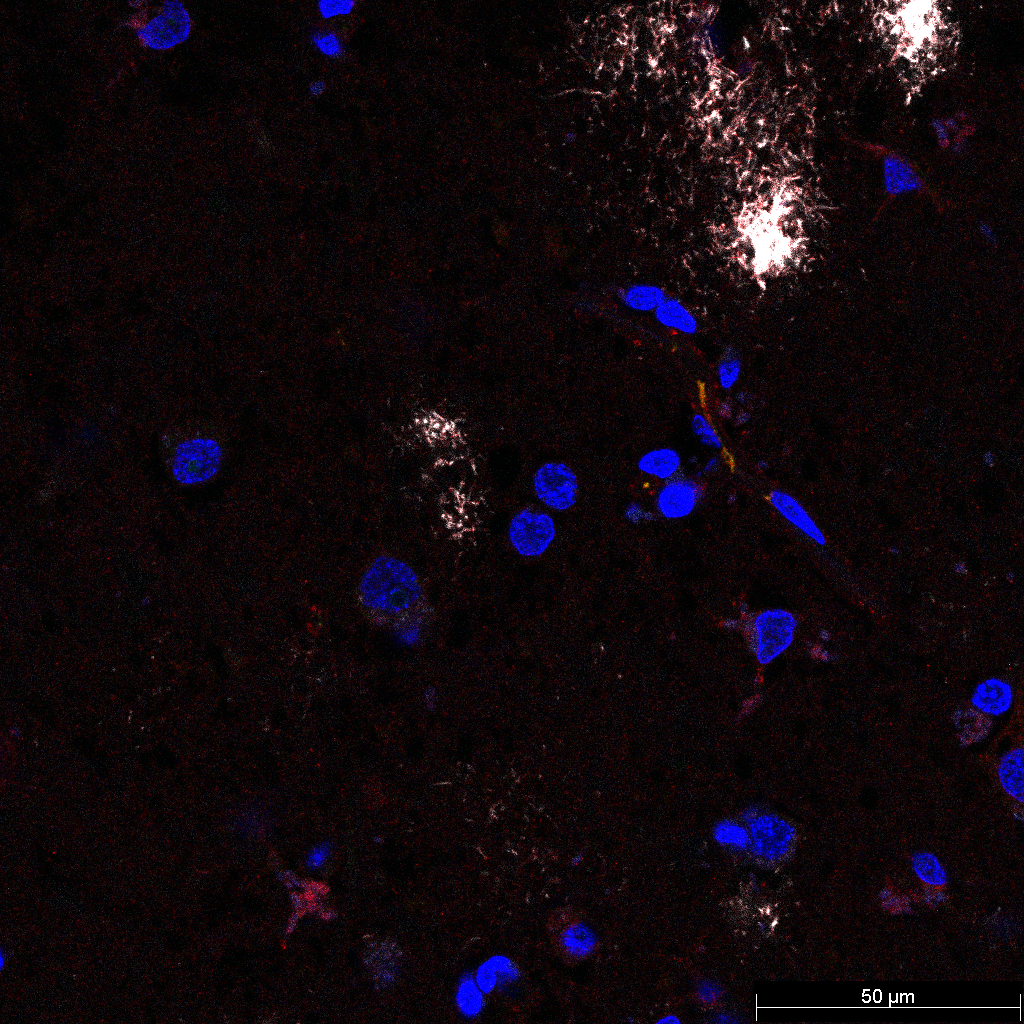

Supplement: Supplementary file 9 — Source Data for Figure 1 [file EMBR-24-e56467-s014.zip › Fig. 1 Source data/Fig 1A-C IF/Fig 1A apoE44 P2 Right.tif]

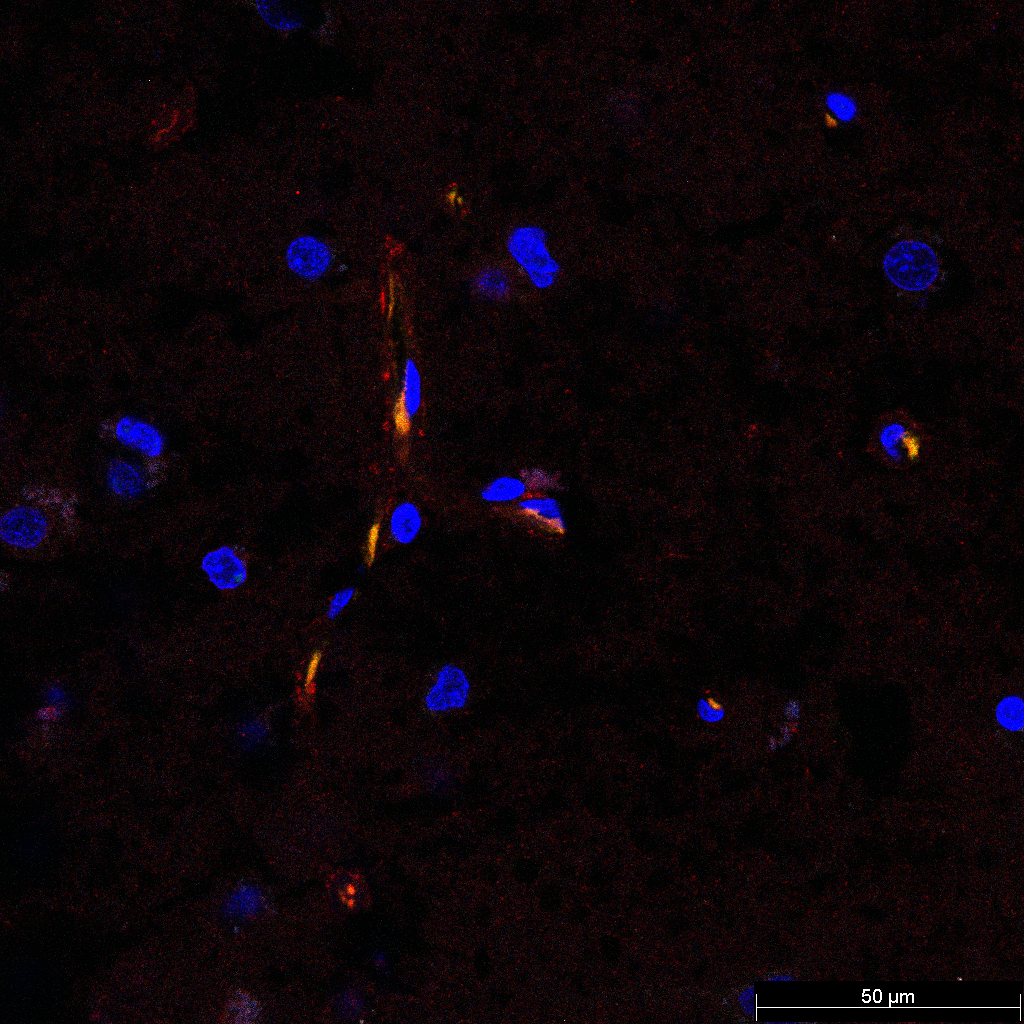

Supplement: Supplementary file 9 — Source Data for Figure 1 [file EMBR-24-e56467-s014.zip › Fig. 1 Source data/Fig 1A-C IF/Fig 1B apoE23 P1 Left.tif]

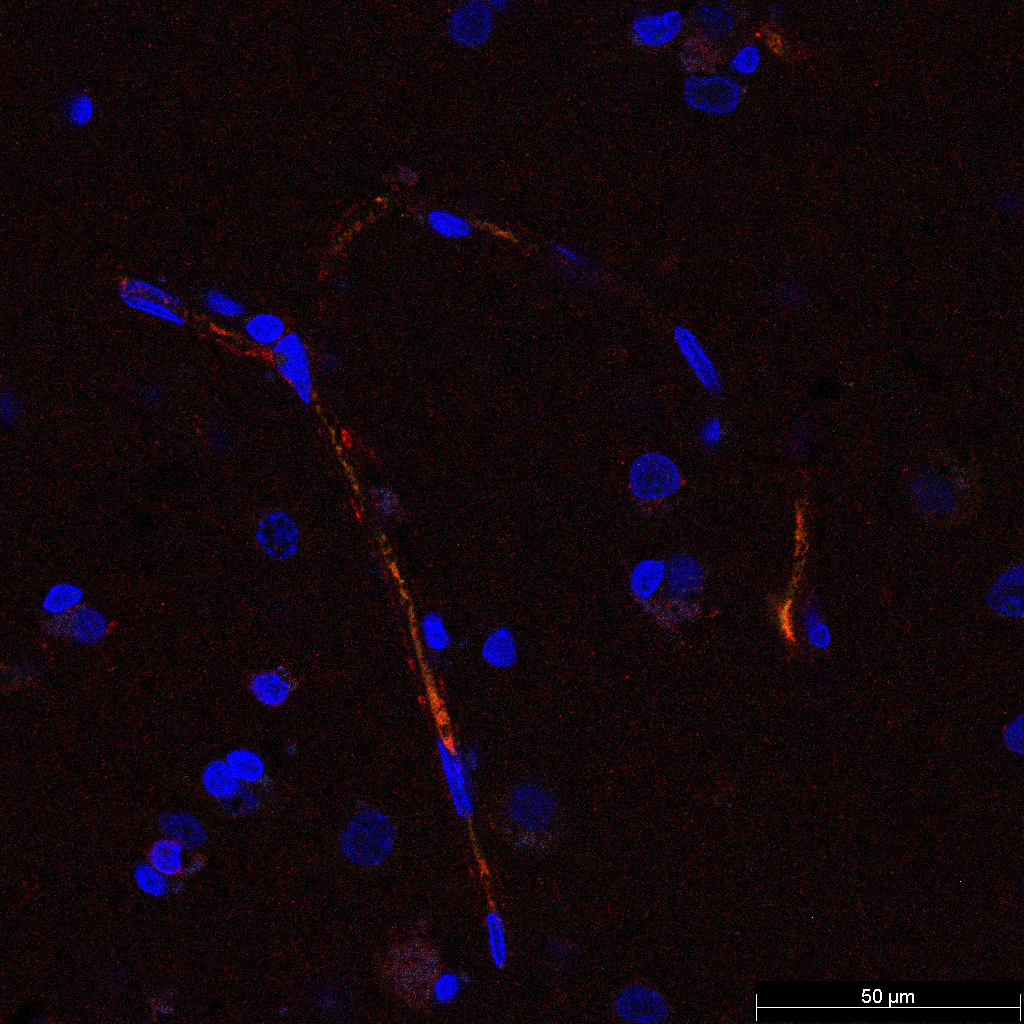

Supplement: Supplementary file 9 — Source Data for Figure 1 [file EMBR-24-e56467-s014.zip › Fig. 1 Source data/Fig 1A-C IF/Fig 1B apoE23 P1 Right.tif]

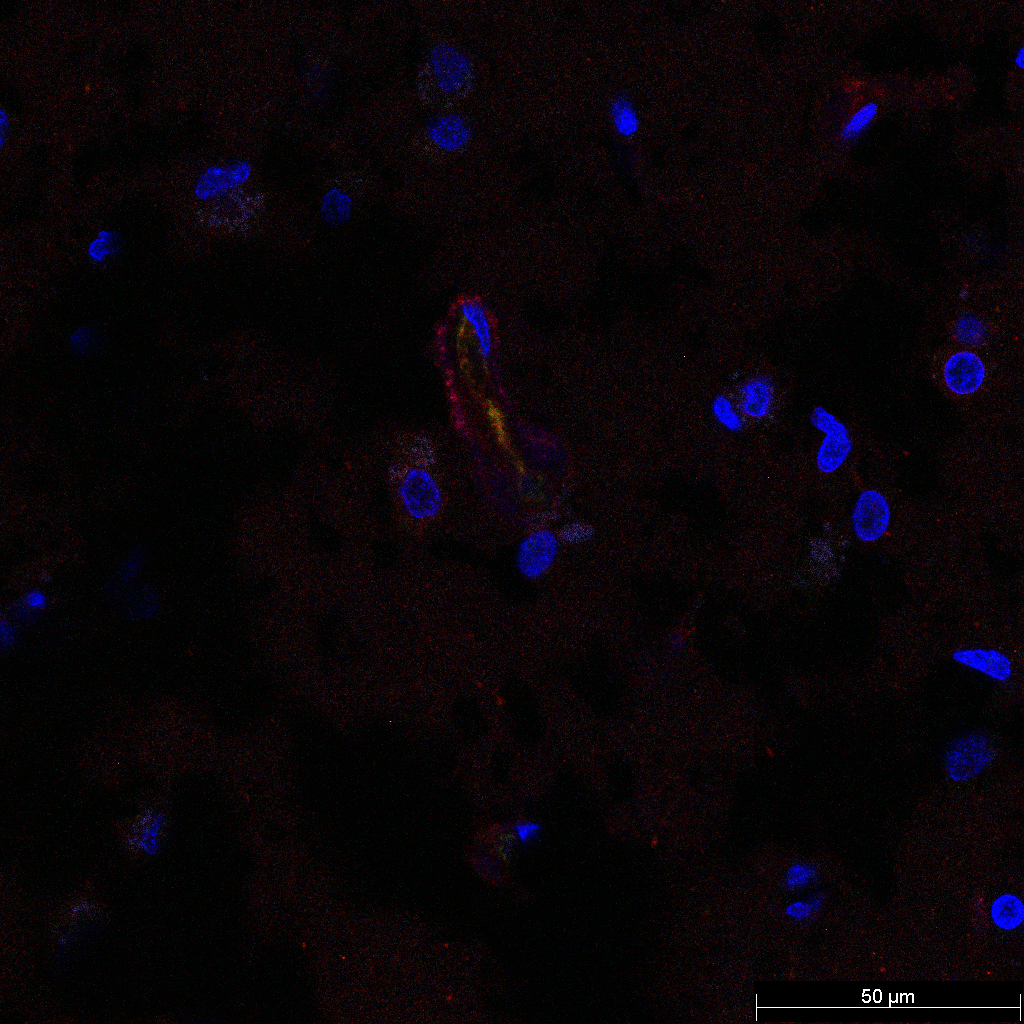

Supplement: Supplementary file 9 — Source Data for Figure 1 [file EMBR-24-e56467-s014.zip › Fig. 1 Source data/Fig 1A-C IF/Fig 1B apoE23 P2 Left.tif]

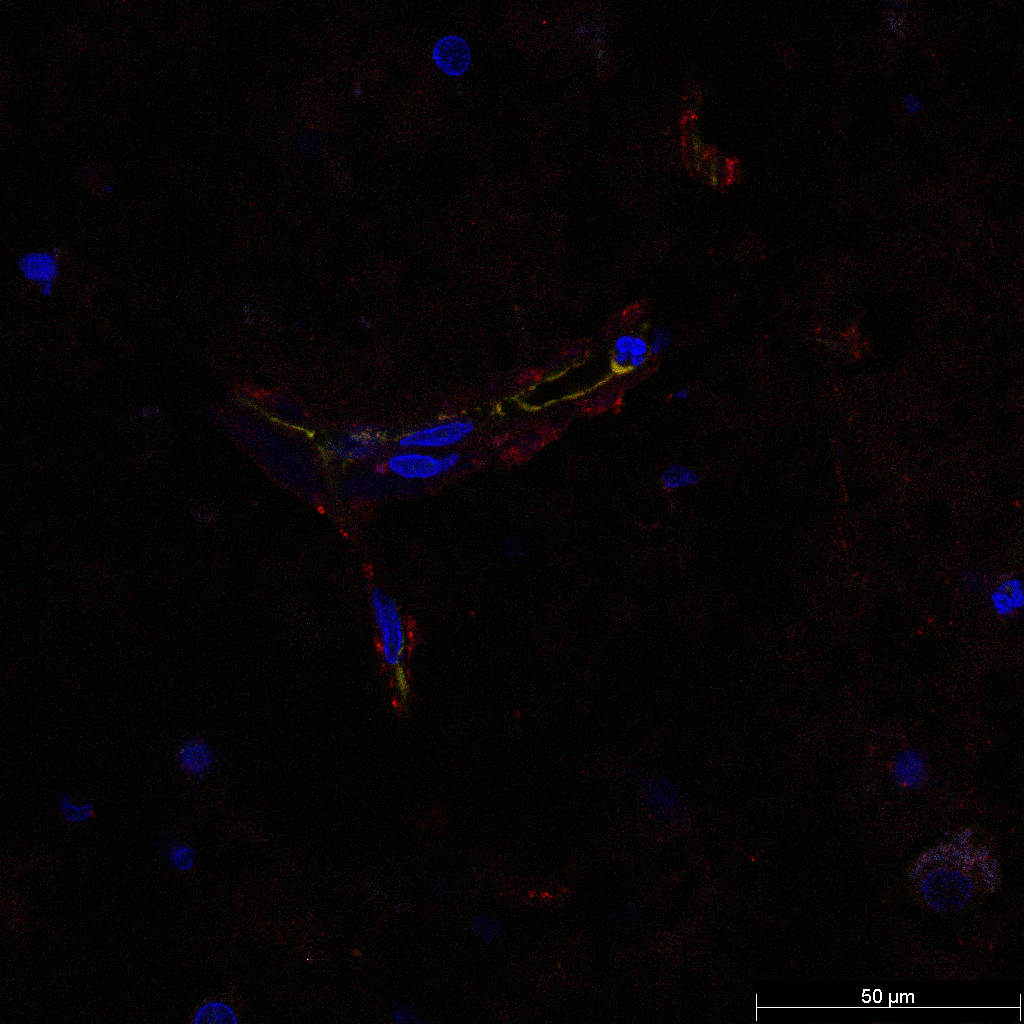

Supplement: Supplementary file 9 — Source Data for Figure 1 [file EMBR-24-e56467-s014.zip › Fig. 1 Source data/Fig 1A-C IF/Fig 1B apoE23 P2 Right.tif]

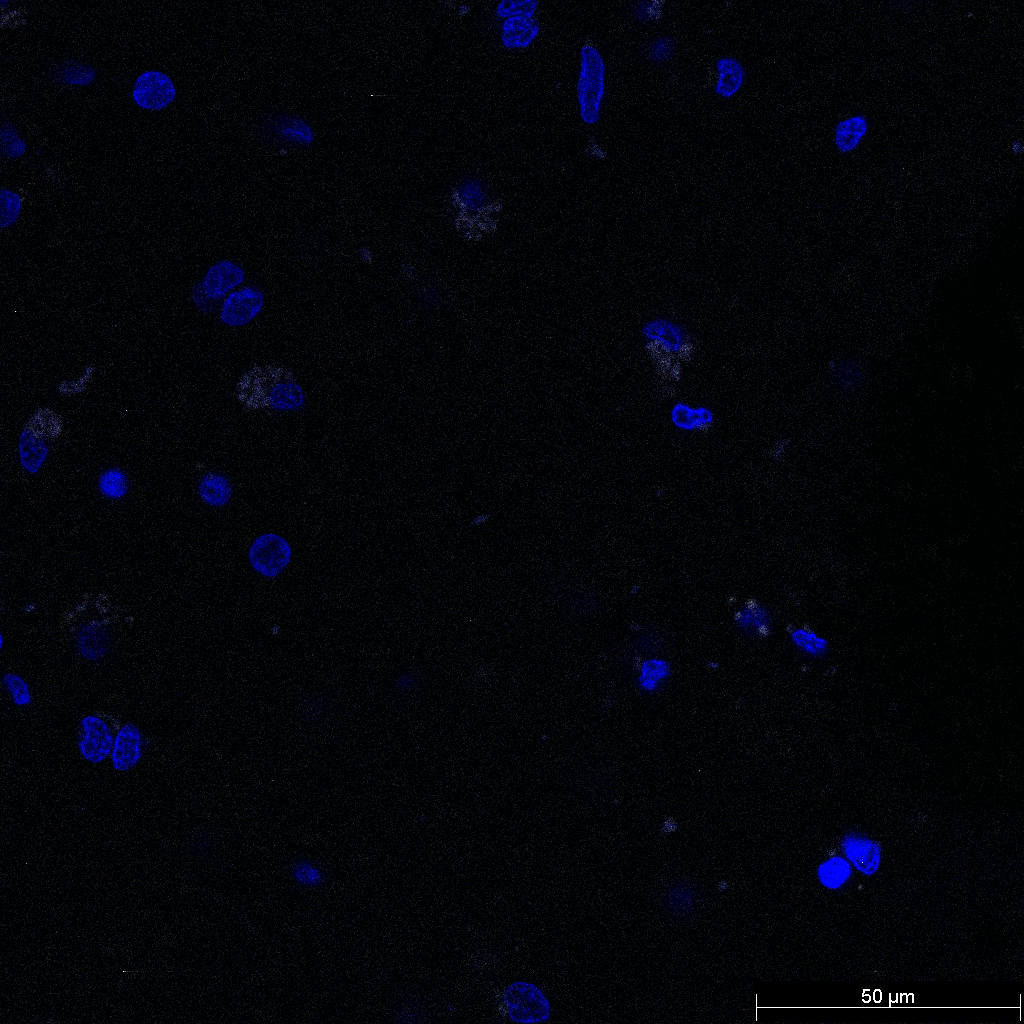

Supplement: Supplementary file 9 — Source Data for Figure 1 [file EMBR-24-e56467-s014.zip › Fig. 1 Source data/Fig 1A-C IF/Fig 1C negative control.tif]

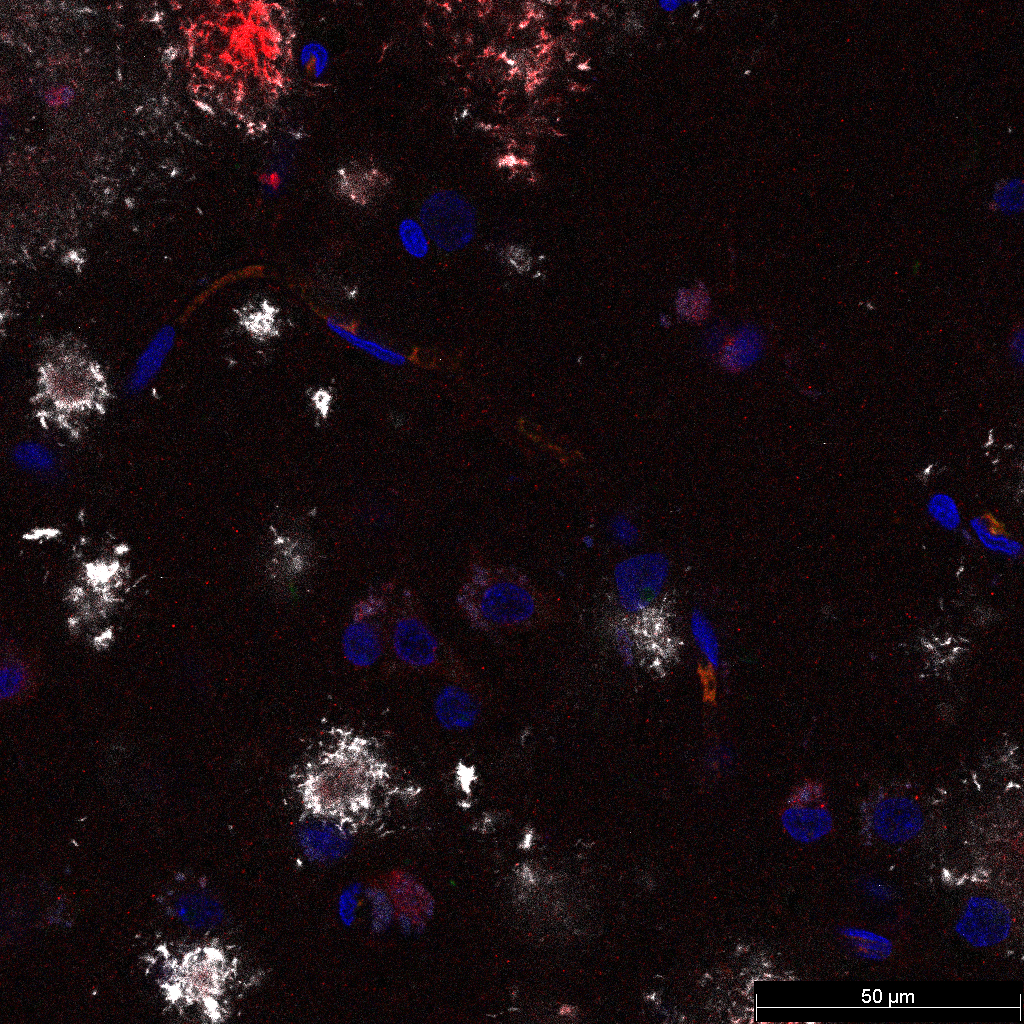

Supplement: Supplementary file 9 — Source Data for Figure 1 [file EMBR-24-e56467-s014.zip › Fig. 1 Source data/Fig 1A-C IF/Fig. 1C apoE44 AD Left.tif]

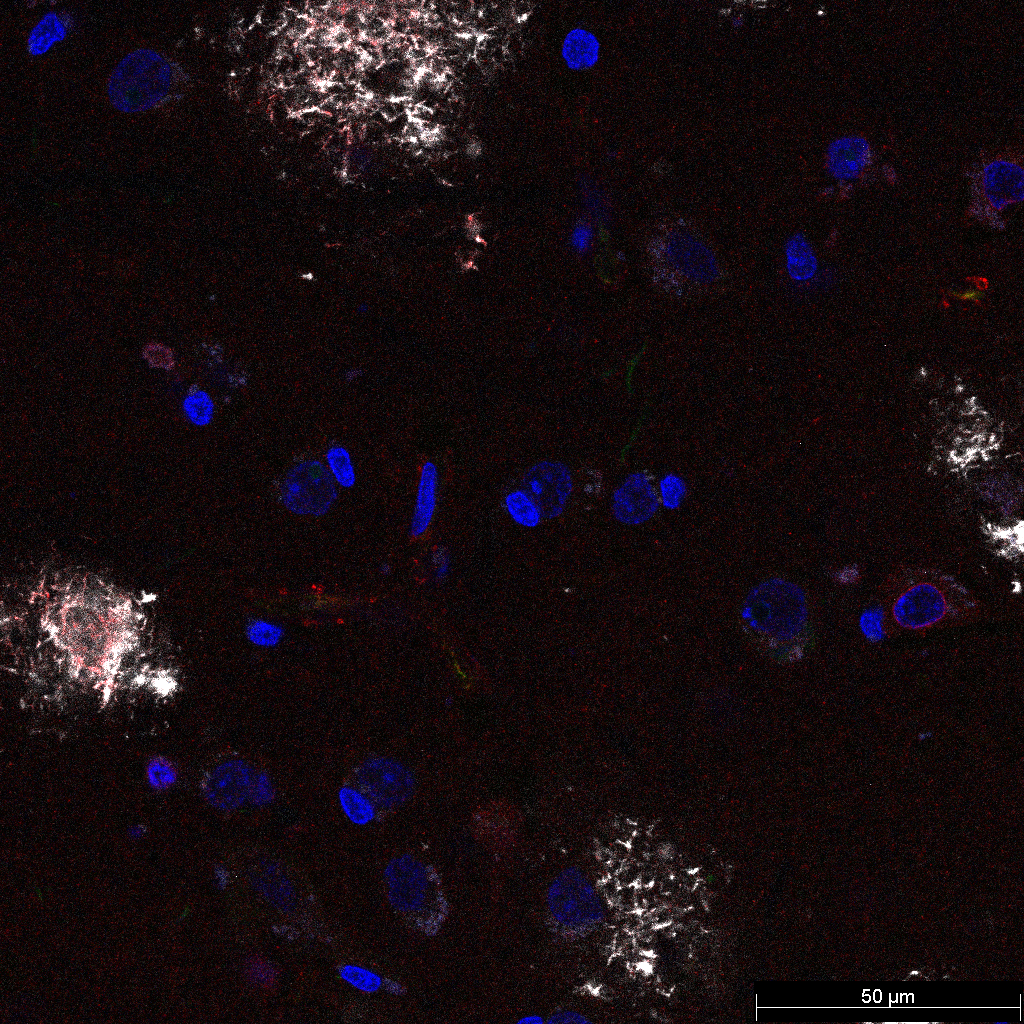

Supplement: Supplementary file 9 — Source Data for Figure 1 [file EMBR-24-e56467-s014.zip › Fig. 1 Source data/Fig 1A-C IF/Fig. 1C apoE44 AD Right.tif]

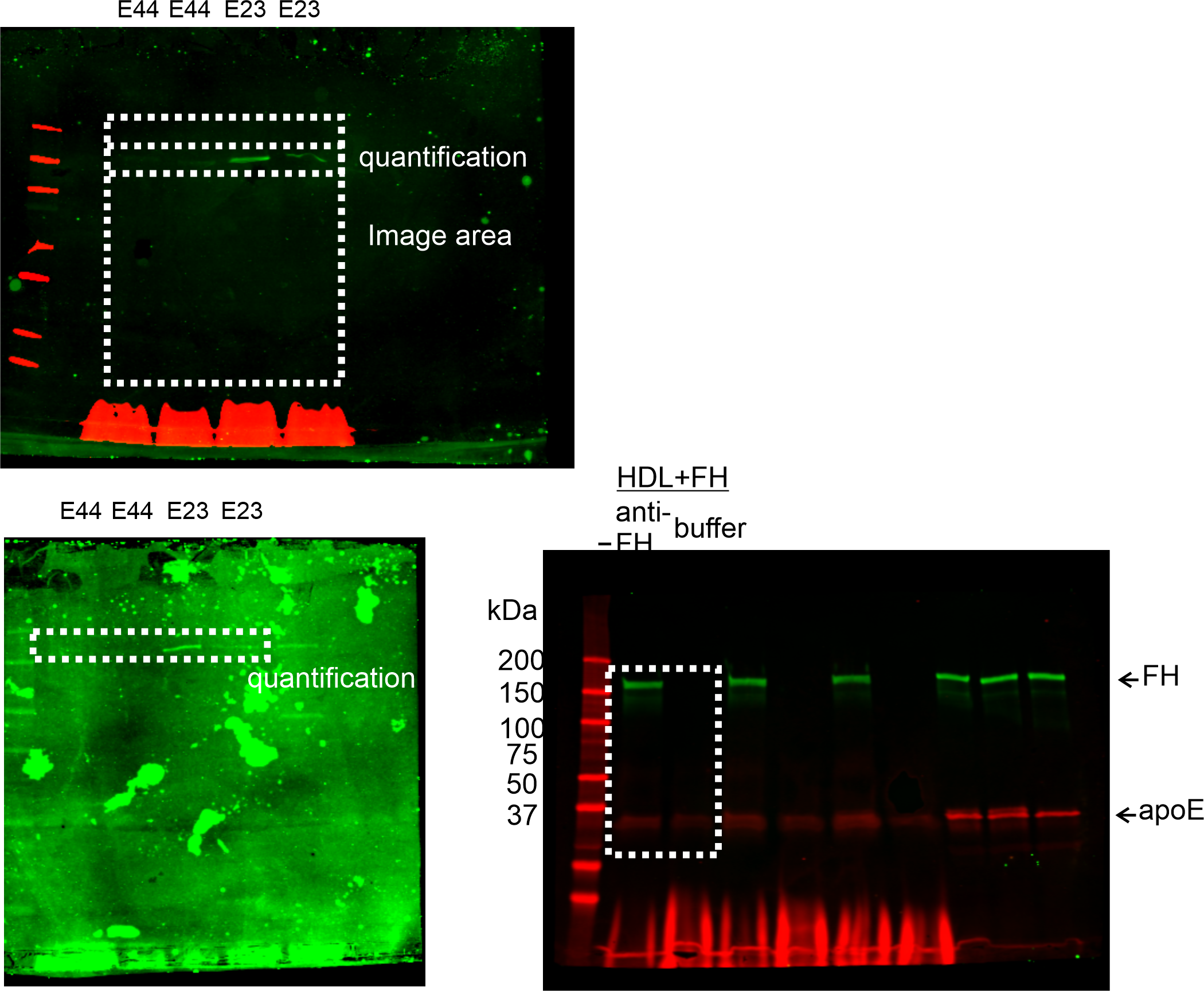

Supplement: Supplementary file 9 — Source Data for Figure 1 [file EMBR-24-e56467-s014.zip › Fig. 1 Source data/Fig 1E gels.tif]

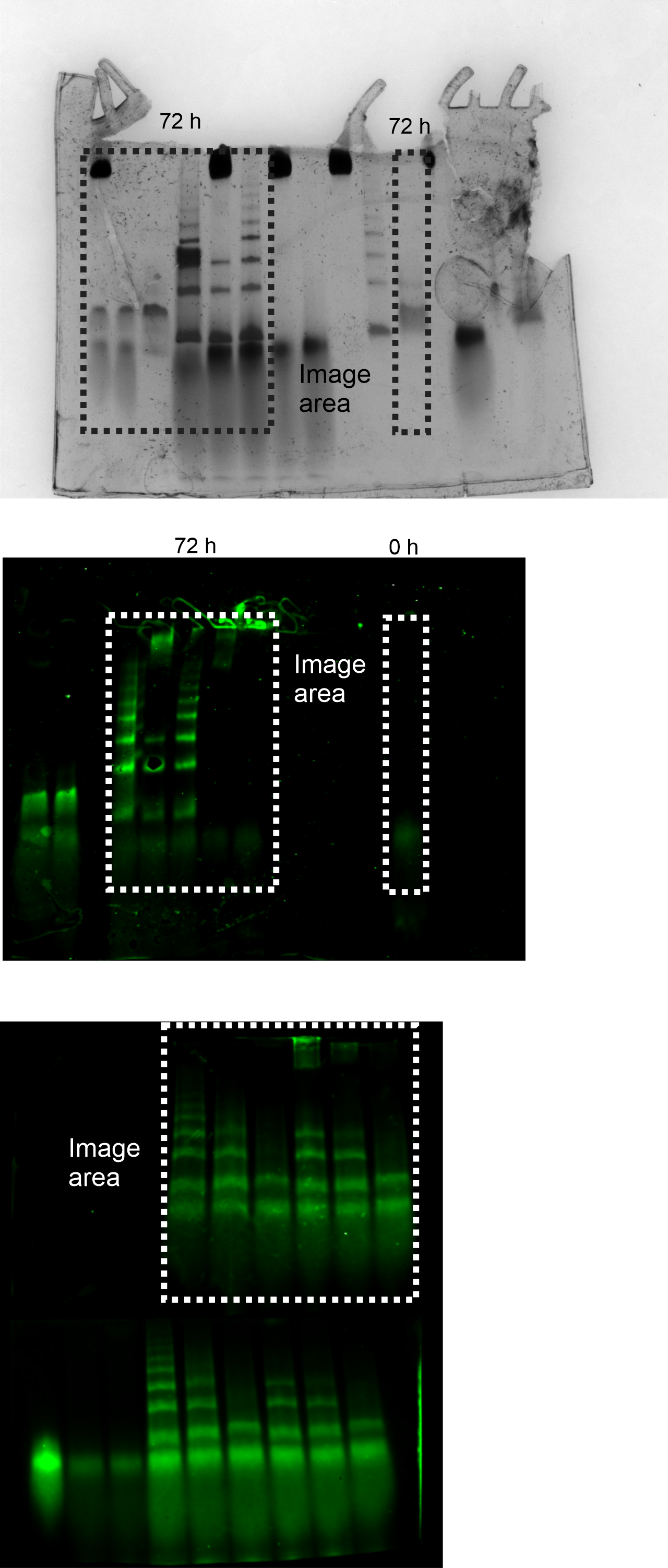

Supplement: Supplementary file 10 — Source Data for Figure 2 [file EMBR-24-e56467-s008.zip › Fig. 2 Source data/Fig 2B gels.tif]

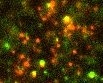

Supplement: Supplementary file 10 — Source Data for Figure 2 [file EMBR-24-e56467-s008.zip › Fig. 2 Source data/Fig. 2C.tif]

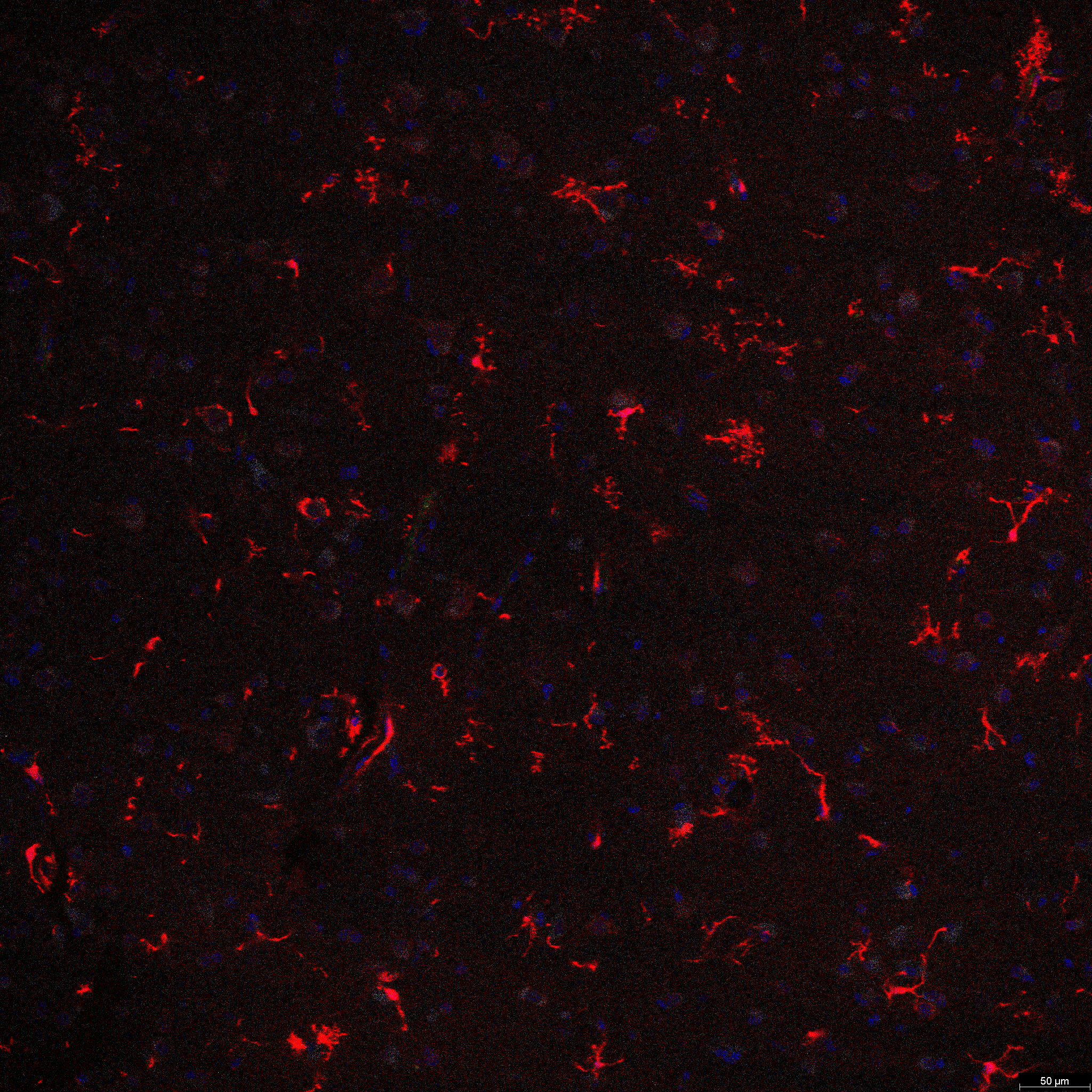

Supplement: Supplementary file 11 — Source Data for Figure 3 [file EMBR-24-e56467-s003.zip › Fig. 3 Source data/Fig 3A and B IF/Fig 3A apoE23 Left.tif]

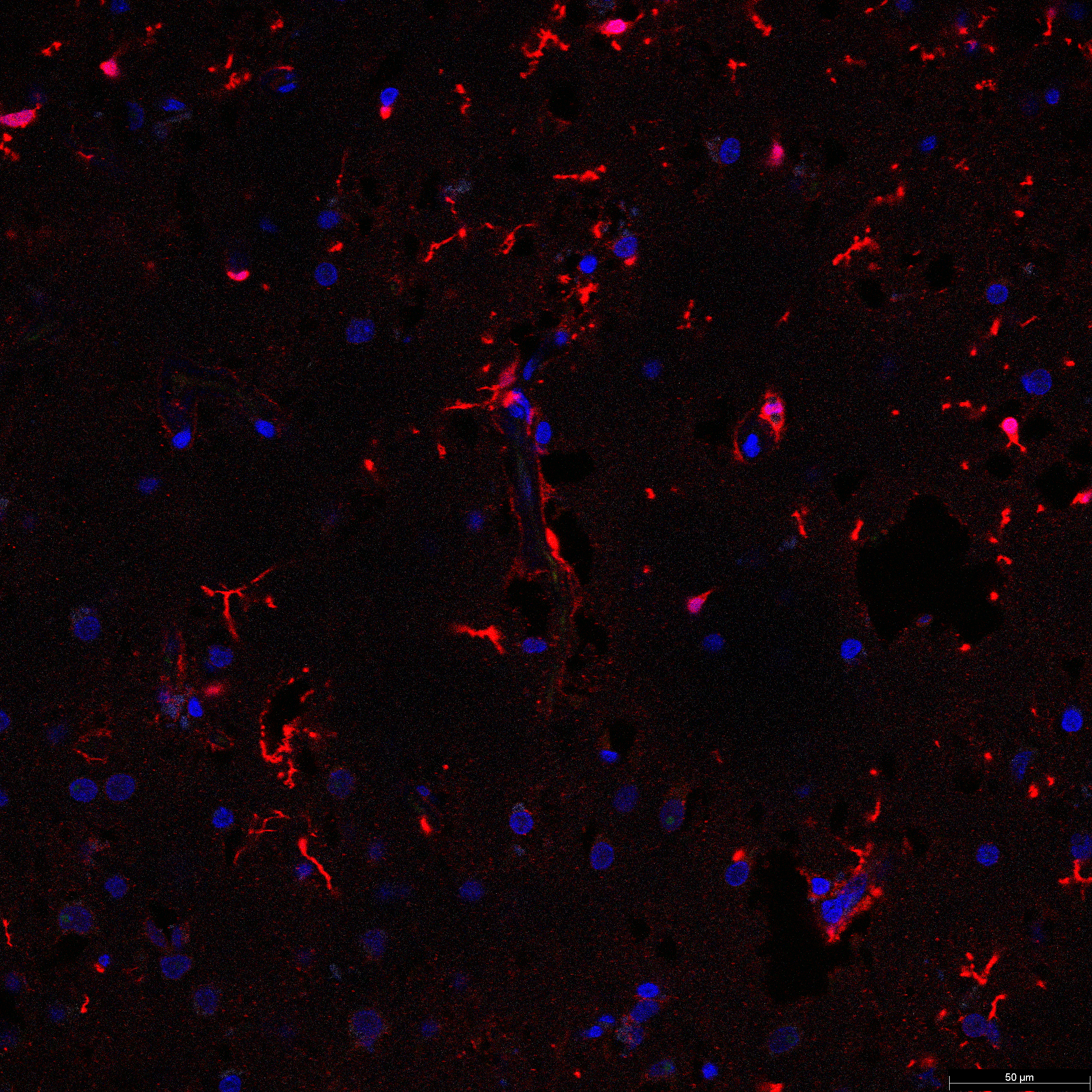

Supplement: Supplementary file 11 — Source Data for Figure 3 [file EMBR-24-e56467-s003.zip › Fig. 3 Source data/Fig 3A and B IF/Fig 3A apoE23 Middle.tif]

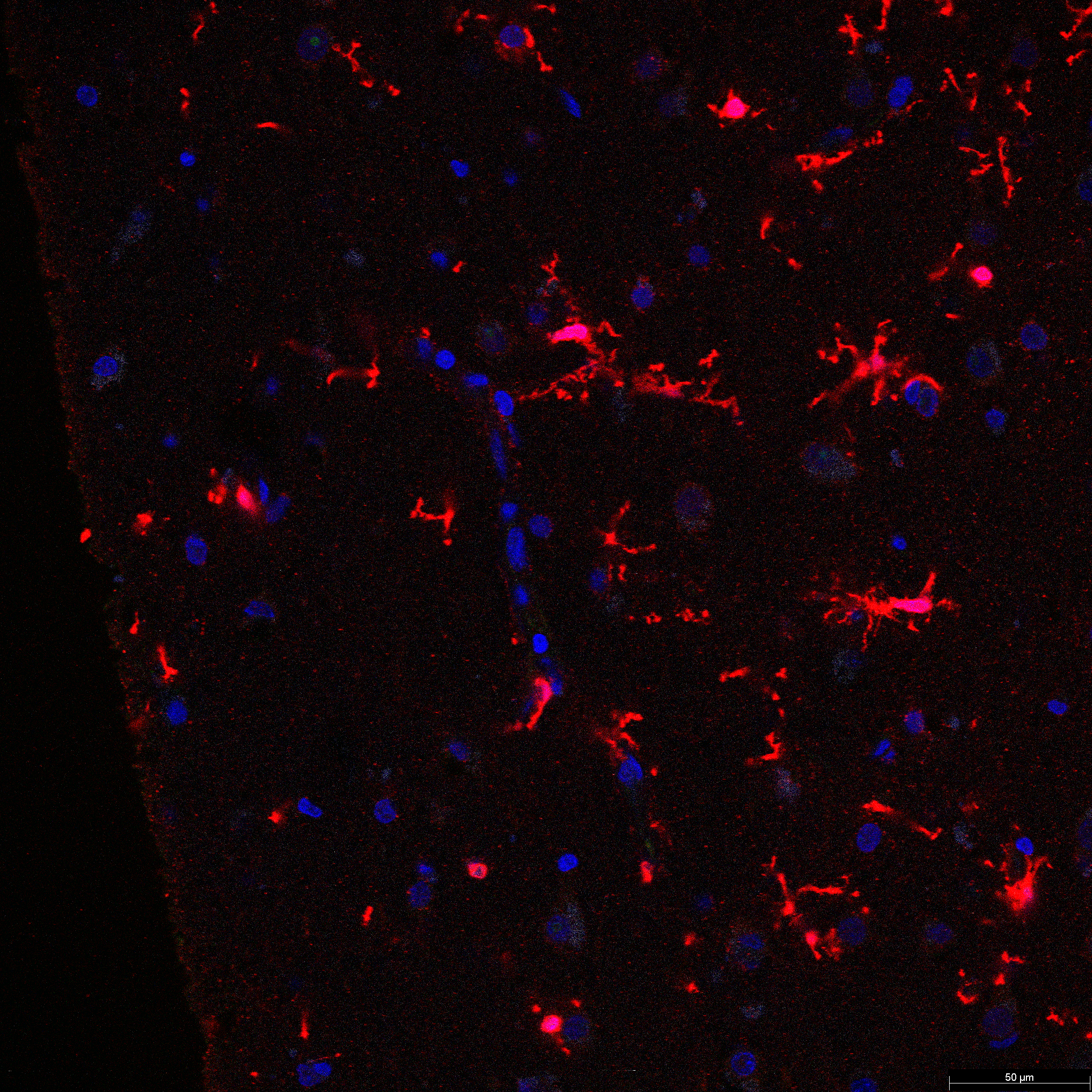

Supplement: Supplementary file 11 — Source Data for Figure 3 [file EMBR-24-e56467-s003.zip › Fig. 3 Source data/Fig 3A and B IF/Fig 3A apoE23 Right.tif]

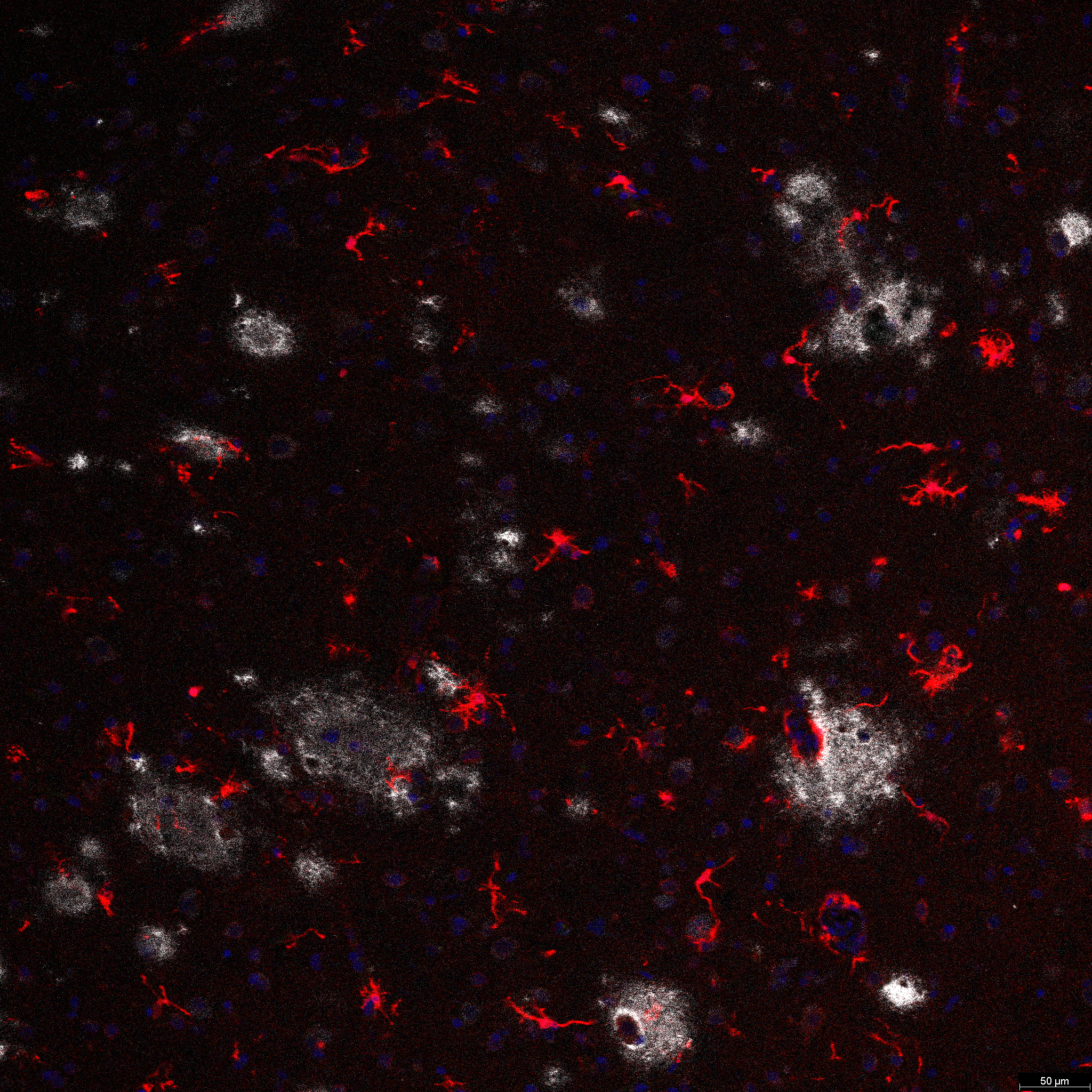

Supplement: Supplementary file 11 — Source Data for Figure 3 [file EMBR-24-e56467-s003.zip › Fig. 3 Source data/Fig 3A and B IF/Fig 3B apoE44 Left.tif]

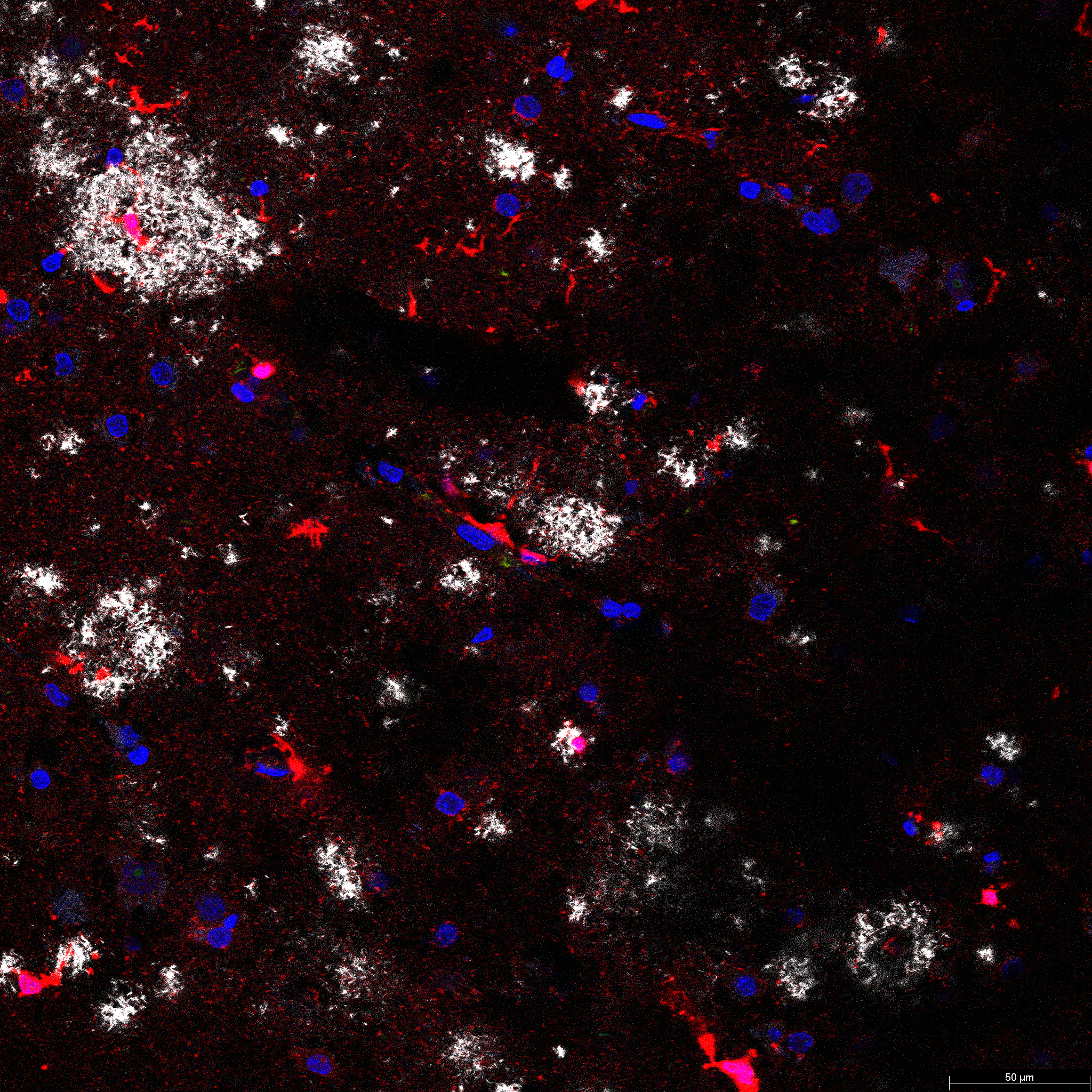

Supplement: Supplementary file 11 — Source Data for Figure 3 [file EMBR-24-e56467-s003.zip › Fig. 3 Source data/Fig 3A and B IF/Fig 3B apoE44 Middle.tif]

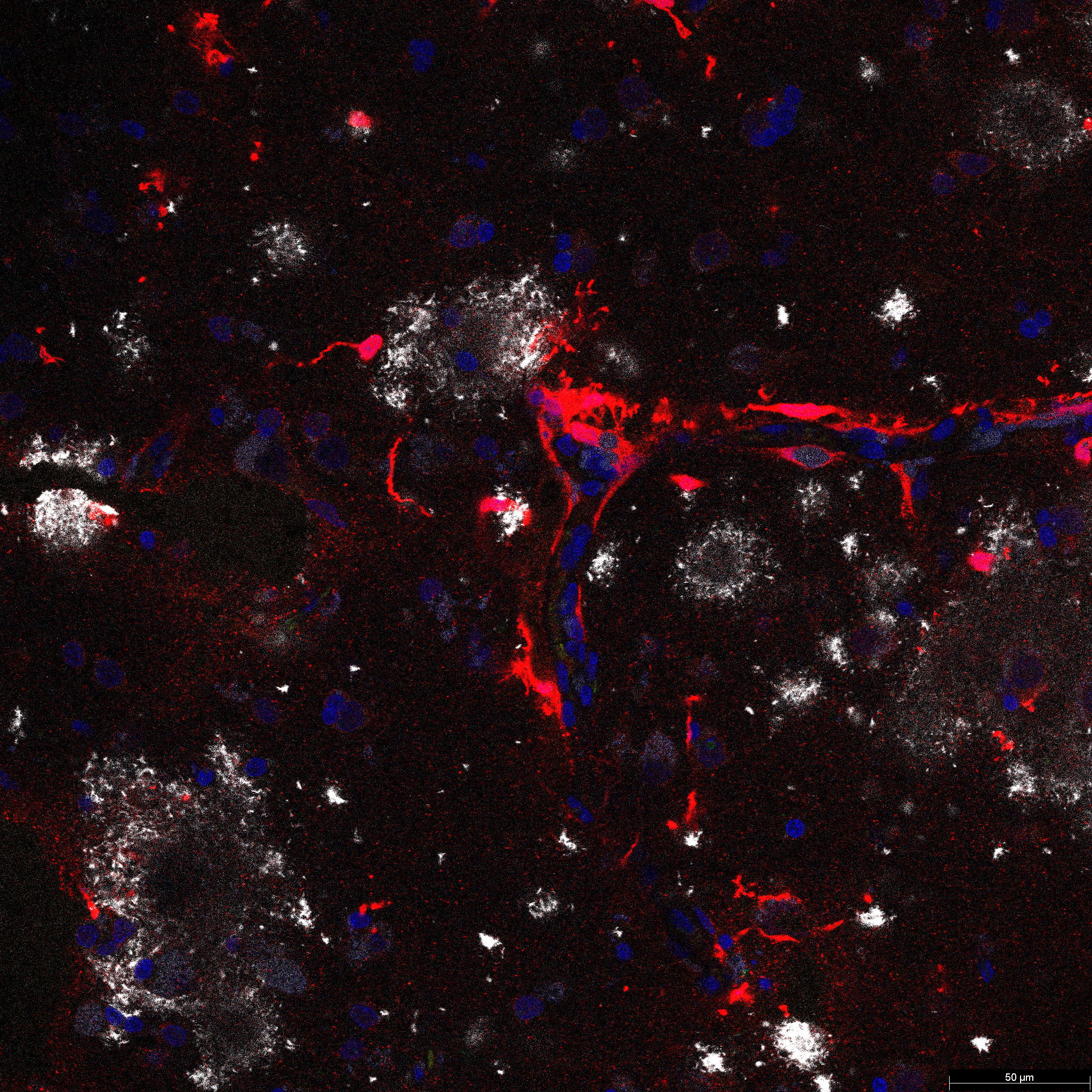

Supplement: Supplementary file 11 — Source Data for Figure 3 [file EMBR-24-e56467-s003.zip › Fig. 3 Source data/Fig 3A and B IF/Fig 3B apoE44 Right.tif]

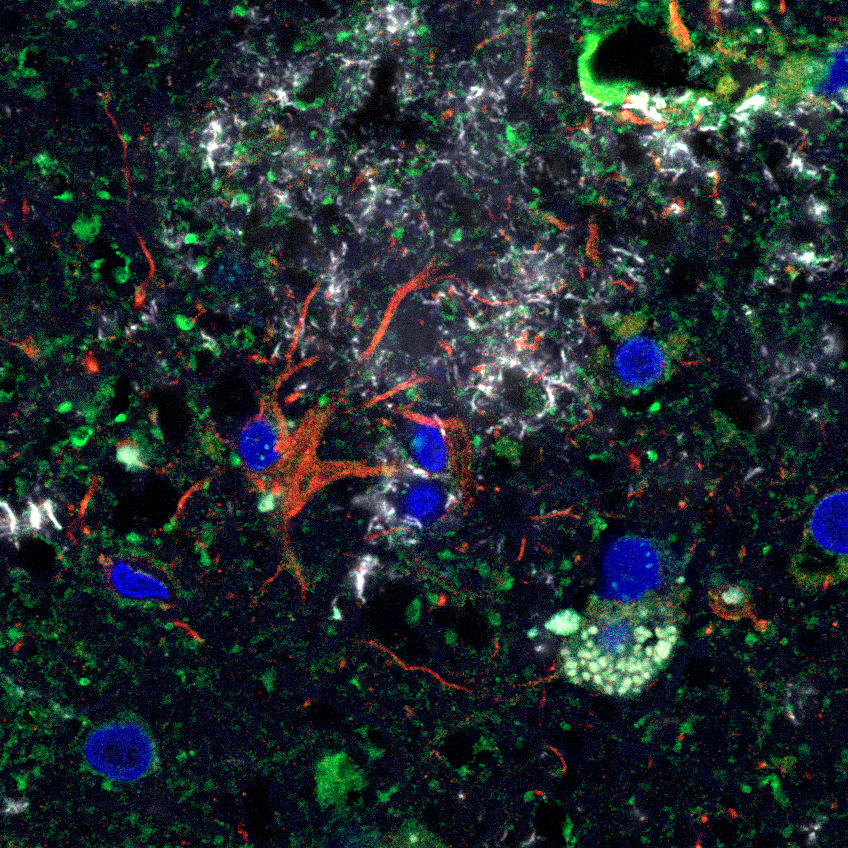

Supplement: Supplementary file 12 — Source Data for Figure 4 [file EMBR-24-e56467-s005.zip › Fig. 4 Source data/Fig 4B Image.tiff]

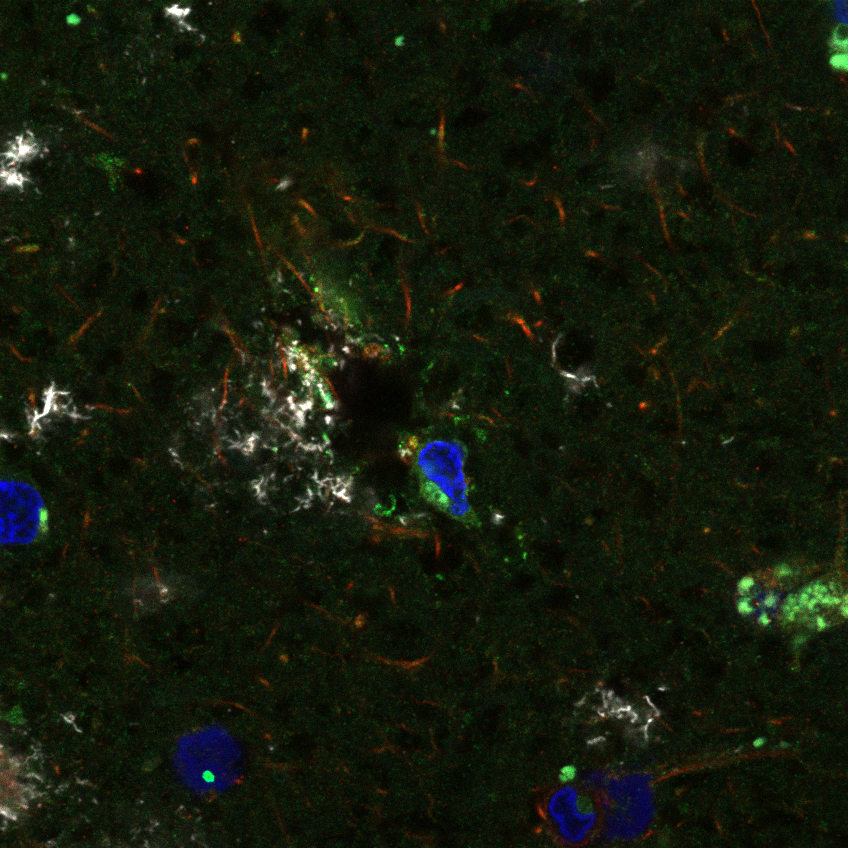

Supplement: Supplementary file 12 — Source Data for Figure 4 [file EMBR-24-e56467-s005.zip › Fig. 4 Source data/Fig. 4A Image.tiff]
