# Supplementary material for: Reduced binding of apoE4 to complement factor H promotes amyloid‐β oligomerization and neuroinflammation
Source: EMBO Rep. 2023 May 8;24(7):e56467. doi: 10.15252/embr.202256467 (PMC10328077; doi:10.15252/embr.202256467)
Supplement: Supplementary file 15 [file EMBR-24-e56467-s013.pdf]

EMBO reports

Reduced binding of apoE4 to complement factor H promotes amyloid-β oligomerization and neuroinflammation

Larisa Chernyaeva, Giorgio Ratti, Laura Teirilä, Satoshi Fudo, Uni Rankka, Anssi Pelkonen, Paula Korhonen, Katarzyna Leskinen, Salla Keskitalo, Kari Salokas, Christina Gkolfinopoulou, Katrina Crompton, Matti Javanainen, Lotta Happonen, Markku Varjosalo, Tarja Malm, Ville Leinonen, Angelika Chroni, Päivi Saavalainen, Seppo Meri, Tommi Kajander, Adam Wollman, Eija Nissilä, and Karita Haapasalo
DOI: 10.15252/embr.202256467

Corresponding author(s): Karita Haapasalo (karita.haapasalo@helsinki.fi)

Review Timeline:

Submission Date:	11th Nov 22
Editorial Decision:	23rd Dec 22
Revision Received:	1st Mar 23
Editorial Decision:	6th Apr 23
Revision Received:	8th Apr 23
Accepted:	24th Apr 23

Editor: Martina Rembold

Transaction Report:

(Note: With the exception of the correction of typographical or spelling errors that could be a source of ambiguity, letters and reports are not edited. Depending on transfer agreements, referee reports obtained elsewhere may or may not be included in this compilation. Referee reports are anonymous unless the Referee chooses to sign their reports.)

Dear Dr. Haapasalo

Thank you for the submission of your research manuscript to our journal. We have now received the full set of referee reports that is copied below.

As you will see, the referees acknowledge that the findings are potentially interesting, but they also raise a number of concerns and have a number of suggestions how to strengthen your data, which should be addressed.

I am also available to discuss the revisions further via e-mail or video call, if you wish.

Given these constructive comments, we would like to invite you to revise your manuscript with the understanding that the referee concerns (as detailed above and in their reports) must be fully addressed and their suggestions taken on board. Please address all referee concerns in a complete point-by-point response. Acceptance of the manuscript will depend on a positive outcome of a second round of review. It is EMBO reports policy to allow a single round of revision only and acceptance or rejection of the manuscript will therefore depend on the completeness of your responses included in the next, final version of the manuscript.

We realize that it is difficult to revise to a specific deadline. In the interest of protecting the conceptual advance provided by the work, we recommend a revision within 3 months (March 23). Please discuss the revision progress ahead of this time with the editor if you require more time to complete the revisions.

*****IMPORTANT NOTE:
We perform an initial quality control of all revised manuscripts before re-review. Your manuscript will FAIL this control and the handling will be DELAYED if the following APPLIES:

1) A data availability section providing access to data deposited in public databases is missing. If you have not deposited any data, please add a sentence to the data availability section that explains that.

2) Your manuscript contains statistics and error bars based on n=2. Please use scatter blots in these cases. No statistics should be calculated if n=2.

When submitting your revised manuscript, please carefully review the instructions that follow below. Failure to include requested items will delay the evaluation of your revision.*****

When submitting your revised manuscript, we will require:

1) a .docx formatted version of the manuscript text (including legends for main figures, EV figures and tables). Please make sure that the changes are highlighted to be clearly visible.

2) individual production quality figure files as .eps, .tif, .jpg (one file per figure).
Please download our Figure Preparation Guidelines (figure preparation pdf) from our Author Guidelines pages https://www.embopress.org/page/journal/14693178/authorguide for more info on how to prepare your figures.

3) a .docx formatted letter INCLUDING the reviewers' reports and your detailed point-by-point responses to their comments. As part of the EMBO Press transparent editorial process, the point-by-point response is part of the Review Process File (RPF), which will be published alongside your paper.

4) a complete author checklist, which you can download from our author guidelines (). Please insert information in the checklist that is also reflected in the manuscript. The completed author checklist will also be part of the RPF.

5) Please note that all corresponding authors are required to supply an ORCID ID for their name upon submission of a revised manuscript (). Please find instructions on how to link your ORCID ID to your account in our manuscript tracking system in our Author guidelines
()

6) We replaced Supplementary Information with Expanded View (EV) Figures and Tables that are collapsible/expandable online. A maximum of 5 EV Figures can be typeset. EV Figures should be cited as 'Figure EV1, Figure EV2" etc... in the text and their respective legends should be included in the main text after the legends of regular figures.

- For the figures that you do NOT wish to display as Expanded View figures, they should be bundled together with their legends in a single PDF file called *Appendix*, which should start with a short Table of Content. Appendix figures should be referred to in the main text as: "Appendix Figure S1, Appendix Figure S2" etc. See detailed instructions regarding expanded view here:

- Additional Tables/Datasets should be labeled and referred to as Table EV1, Dataset EV1, etc. Legends have to be provided in a separate tab in case of .xls files. Alternatively, the legend can be supplied as a separate text file (README) and zipped together with the Table/Dataset file.

7) Before submitting your revision, primary datasets such as the RNAseq analysis produced in this study need to be deposited in an appropriate public database (see < https://www.embopress.org/page/journal/14693178/authorguide#dataavailability>).

Please remember to provide a reviewer password if the datasets are not yet public.

The accession numbers and database should be listed in a formal "Data Availability " section (placed after Materials & Method) that follows the model below (see also < https://www.embopress.org/page/journal/14693178/authorguide#dataavailability>). Please note that the Data Availability Section is restricted to new primary data that are part of this study.

Data availability

The datasets (and computer code) produced in this study are available in the following databases:

- RNA-Seq data: Gene Expression Omnibus GSE46843 (https://www.ncbi.nlm.nih.gov/geo/query/acc.cgi?acc=GSE46843)
- [data type]: [name of the resource] [accession number/identifier/doi] ([URL or identifiers.org/DATABASE:ACCESSION])

*** Note - All links should resolve to a page where the data can be accessed. ***

8) At EMBO Press we ask authors to provide source data for the main figures. Our source data coordinator will contact you to discuss which figure panels we would need source data for and will also provide you with helpful tips on how to upload and organize the files.

Additional information on source data and instruction on how to label the files are available .

9) The journal requires a statement specifying whether or not authors have competing interests (defined as all potential or actual interests that could be perceived to influence the presentation or interpretation of an article). In case of competing interests, this must be specified in your disclosure statement. Further information: https://www.embopress.org/competing-interests

10) Figure legends and data quantification:
The following points must be specified in each figure legend:

- the name of the statistical test used to generate error bars and P values,
- the number (n) of independent experiments (please specify technical or biological replicates) underlying each data point,
- the nature of the bars and error bars (s.d., s.e.m.)

- If the data are obtained from n {less than or equal to} 5, show the individual data points in addition to the SD or SEM.
- If the data are obtained from n {less than or equal to} 2, use scatter blots showing the individual data points.

Discussion of statistical methodology can be reported in the materials and methods section, but figure legends should contain a basic description of n, P and the test applied.

See also the guidelines for figure legend preparation:
https://www.embopress.org/page/journal/14693178/authorguide#figureformat

- Please also include scale bars in all microscopy images.

11) Our journal encourages inclusion of *data citations in the reference list* to directly cite datasets that were re-used and obtained from public databases. Data citations in the article text are distinct from normal bibliographical citations and should directly link to the database records from which the data can be accessed. In the main text, data citations are formatted as follows: "Data ref: Smith et al, 2001" or "Data ref: NCBI Sequence Read Archive PRJNA342805, 2017". In the Reference list, data citations must be labeled with "[DATASET]". A data reference must provide the database name, accession number/identifiers and a resolvable link to the landing page from which the data can be accessed at the end of the reference. Further instructions are available at .

12) As part of the EMBO publication's Transparent Editorial Process, EMBO reports publishes online a Review Process File to accompany accepted manuscripts. This File will be published in conjunction with your paper and will include the referee reports, your point-by-point response and all pertinent correspondence relating to the manuscript.

You are able to opt out of this by letting the editorial office know (emboreports@embo.org). If you do opt out, the Review Process File link will point to the following statement: "No Review Process File is available with this article, as the authors have chosen not to make the review process public in this case."

We would also welcome the submission of cover suggestions, or motifs to be used by our Graphics Illustrator in designing a cover.

I look forward to seeing a revised form of your manuscript when it is ready. Please use this link to submit your revision: https://embor.msubmit.net/cgi-bin/main.plex

Yours sincerely,

Martina Rembold, PhD
Senior Editor
EMBO reports

****

Referee #1:

In this study, Chernyaeva et al. explored how ApoE interacts with complement regulator factor H (FH) to impact amyloid beta (Aβ) toxicity, plaque formation, and neuroinflammation. Leveraging a combination of transcriptomics, biochemical, and flow cytometric approaches they report here that binding of FH to ApoE reduces ApoE/Aβ1-42 oligomerization, complement activation, phagocytosis, and toxicity of ApoE/Aβ1-42 complexes. Furthermore, they show that FH forms complement-resistant ApoE oligomers with ApoE/Aβ1-42 complexes in an isoform-specific manner (apoE2>apoE4), which leads to reduced Aβ toxicity. Overall, I am quite enthusiastic about this work given their compelling findings and the sound nature of their studies. However, several issues were noted, which I believe need to be addressed in order to solidify their central findings and conclusions.
Specific comments:
1. Additional analysis is needed to corroborate the ability of FH to augment Aβ-mediated inflammation. This effect on neuroinflammation is highlighted in multiple key places throughout the manuscript including the title, abstract, and results section; however, they have not performed a comprehensive assessment of the key neuroinflammatory molecules that have been classically associated with Aβ-mediated neuroinflammation (e.g. proinflammatory cytokines, reactive oxygen species, etc.). The major readouts currently exploited by them to conclude an effect on neuroinflammation appear to be the generation of complement activation markers. A more comprehensive evaluation of the changes in key Alzheimer's disease-related inflammatory markers, other than just the complement pathway, would help to increase both the scope and impact of this work.
2. To increase the readability of their figures, they may want to consider including colored denotations of the ligands that were stained as opposed to highlighting the fluorochromes. For instance, DAPI written in blue, FH written in green, etc.
3. In the figure legends (i.e. Figure 1A-B, 3A-B) it would be helpful if they briefly indicated what regions of the brains were stained in these studies.
4. In regards to the statement that spans lines 89-91, it would be interesting to know whether any of the reported mutations or polymorphisms in FH have been linked to Alzheimer's disease or any other neurological disorders.
5. In their human immunofluorescence studies, they conclude that all Iba-1-expressing cells are microglia. Given that both microglia and peripheral-derived macrophages can express Iba-1 in the inflamed brain, they should consider clarifying this statement or bolstering their studies with the analysis of other microglia-specific markers.
6. In the first sentence of the second paragraph describing the data presented in Figure 3, they casually make a biological link between CR3 and CD11b. Before discussing the data interpretation in regards to CD11b (Figure 3F and G), it would be better to provide additional background information on the subunit composition of CR3, especially for those outside of the complement field.
7. In relation to the data presented in Figure 4G, they conclude that their findings demonstrating reduced Aβ1-42 phagocytosis point towards a prevention of plaque growth. However, they have not rigorously tested this in their model and it would be better to tone down this conclusion to limit data over-interpretation. Conversely, if they feel as though this is an important facet of their story, they should then conduct more extensive and targeted studies to directly assess this.
8. The transcriptomics data presented in Figure 5 could be quite informative; however, they have only provided a preliminary, surface-level assessment especially of the differences between the two most relevant groups (i.e. Aβ+FH+ApoE2 vs. Aβ+FH+ApoE4). Deeper analysis could be illuminating and provide additional insights into potential neuroinflammatory differences between the groups (see point number one above). At the very least they should consider performing pathway analysis (KEGG, GO, etc.) and providing a volcano plot highlighting major differentially expressed genes between the Aβ+FH+apoE2 and Aβ+FH+apoE4 experimental groups.

9. The 2016 Tarasoff-Conway et al. manuscript that they cite in the Results and Discussion sections to support their assertion that the majority of extracellular Aβ is removed across the BBB is a review article and it would be better to include citations from the primary literature that supports this statement.

Referee #2:

In this manuscript, Chernyaeva and colleagues investigate the relationship of APOE with complement factor H in the context of amyloid beta. The authors focus on idiopathic hydrocephalus, an aging-associated neurodegenerative disease that is characterized by amyloid beta deposition. The authors wanted to investigate whether interactions between APOE and Factor H could regulate the interactions between Abeta and APOE and thus reduce the neurotoxic effects of Abeta. They show that Factor H binds to APOE in an isoform-dependent manner (E2>E3>E4). Binding of Factor H to APOE reduces Abeta42 aggregation, toxicity and its effect on immune activation. The results are new and inform on the importance of complement-APOE interaction in the context of Abeta.
A general comment - the authors seem to weave in and out of iNPH and AD especially in the beginning of the Results section. It would be great if the authors could focus on one line of thought in the Results section. This associative inference may be more appropriately placed in the Discussion section. In addition, it would be appropriate not to refer to presence of Abeta as AD especially in patients (in the absence of tau pathology or dementia).

Some comments on the data:
(1) Fig1A is a little confusing - the key placed inside the panel depicts all ligands in white pseudocolor but the panels have fluors of different colors. Importantly, no APOE (red) is visible on amyloid deposits (white) - this seem to be somewhat confusing as well. Also, CFH is predominantly expressed from endothelial cells - could authors surmise how this is leaked out into the brain parenchyma? In panel 1D, statistics has been done on n=2 - is this statistically acceptable?
An additional piece of data would be useful as authors mention (line 98-100) that iNPH patients show colocalization .... as markers of early AD pathology in vivo'. In lines 112-113, they additionally mention that iNPH is a prodromal form of AD. In light of this, it will be good to compare APOE/FH/Abeta colocalization in AD brains vs iNPH brains.
(2) In Fig 2, please remove the cartoons - these are distracting and did not help. No APOE4 (negative) or APOE3 data is provided for panels C-E. This is important as APOE3 is the reference allele and APOE2 the protective allele as far as Abeta is concerned
Another comment is that since in Fig 1, the authors show that APOE4 does not bind to FH, would it not imply that experiments testing FH binding to APOE4-Abeta will not change anything (Fig 2)?
(3) In Fig 3, confocal microscopy or other type of analysis need to be shown to establish colocalization of FH with microglia and Abeta plaques.
Most importantly, there is no change in FH intensity between the two genotypes (panel E), which contradicts the premise of the paper (APOE isoform dependent binding to FH).
Also, could the authors explain using U937 cells instead of primary microglia?
No data with the reference isoform (APOE3) was provided in Fig. 3D-G.
(4) Usage of the AMD associated mutant is informative but is this relevant to abeta oligomerization?
(5) In Fig 5, TREM2 does not seem to be upregulated in the data - generally Trem2 or sTrem2 levels track with Abeta. Can the authors comment?
(6) Fig. 6 is confusing. Has the proteomics been done on healthy individuals or iNPH individuals? If these are not from iNPH individuals or AD individuals, then is it fair to summarize these results as written in lines 305-307?

Dear Editor,

We thank you for the opportunity to resubmit our manuscript EMBOR-2022-56467V2:" Reduced binding of apoE4 to complement factor H promotes amyloid-β oligomerization and neuroinflammation" to EMBO Reports. We highly respect the effort that the reviewers have done as they have given valuable feedback and suggested essential experiments to support our conclusions.

Please find below the point-to-point responses describing how we have fully addressed the concerns and suggestions pointed out by the reviewers

The data availability section has been included in the manuscript and links for the data for reviewers can be found here:

- For mRNA data the following details are needed to access the data: GEO accession. GSE193513: Go to: https://www.ncbi.nlm.nih.gov/geo/query/acc.cgi?acc=GSE193513
- For proteomic data use the following link: MassIVE MSV000088618 Link: https://massive.ucsd.edu/ProteoSAFe/dataset.jsp?task=595bf56584824151bb0b73e81f3caa73
- Link to MS crosslinking data: http://massive.ucsd.edu/ProteoSAFe/status.jsp?task=40074352d93d41e8859034cc2f677bb0 Access for reviewers: [username and password]
- Molecular dynamics simulation input and output data, see DOI:10.5281/zenodo.7533587. Link: https://doi.org/10.5281/zenodo.7533587

Point-to-point replies

Referee #1:

In this study, Chernyaeva et al. explored how ApoE interacts with complement regulator factor H (FH) to impact amyloid beta (Aβ) toxicity, plaque formation, and neuroinflammation. Leveraging a combination of transcriptomics, biochemical, and flow cytometric approaches they report here that binding of FH to ApoE reduces ApoE/Aβ1-42 oligomerization, complement activation, phagocytosis, and toxicity of ApoE/Aβ1-42 complexes. Furthermore, they show that FH forms complement-resistant ApoE oligomers with ApoE/Aβ1-42 complexes in an isoform-specific manner (apoE2>apoE4), which leads to reduced Aβ toxicity. Overall, I am quite enthusiastic about this work given their compelling findings and the sound nature of their studies. However, several issues were noted, which I believe need to be addressed in order to solidify their central findings and conclusions.

Specific comments:
1. Additional analysis is needed to corroborate the ability of FH to augment Aβ-mediated inflammation. This effect on neuroinflammation is highlighted in multiple key places throughout the manuscript including the title, abstract, and results section; however, they have not performed a comprehensive assessment of the key neuroinflammatory molecules that have been classically associated with Aβ-mediated neuroinflammation (e.g. proinflammatory cytokines, reactive oxygen species, etc.). The major readouts currently exploited by them to conclude an effect on neuroinflammation appear to be the generation of complement activation markers. A more

comprehensive evaluation of the changes in key Alzheimer's disease-related inflammatory markers, other than just the complement pathway, would help to increase both the scope and impact of this work.

We thank the reviewer for these comments. We have now studied both the mRNA and proteomic data for the presence of the classical AD related inflammatory markers to increase the scope of the work.

From the proteomic data we were able to detect inflammatory markers CDK5, S100B and S100A1. Interestingly, the levels of S100B, which has been shown to suppress Aβ aggregation, were increased in E33/23 samples when compared to E44/E34. This observation is now included as Fig 6C in the manuscript and shortly discussed in lines 305-307 along with the included references:

1. *AD markers: Yang, S. H. (2019). "Cellular and Molecular Mediators of Neuroinflammation in Alzheimer Disease." Int Neurourol J 23(Suppl 2): S54-62.*
2. *J. S. Cristovao, V. K. Morris, I. Cardoso, S. S. Leal, J. Martinez, H. M. Botelho, et al. The neuronal S100B protein is a calcium-tuned suppressor of amyloid-beta aggregation*

In Fig 6B we also corrected x-axis title of mRNA transcripts that should be Aβ+FH+E2 and Aβ+FH+E4 not E44/43 and E33/23

In the mRNA data several inflammatory markers were detected: CCL2, CCL3, CCL4, CD36, CDK5, CX3CL1, CXCL8, IL1B, IL6, ITGAM, NFKB1, PTGS1, PTGS2, S100A1, S100A6, S100A8, S100B, TLR2, TLR4, TLR6, TLR9, TNF and TREM2. From these CXCL8 was significantly reduced in the presence of apoE2 while the inflammatory marker S100A6 was reduced in the presence of apoE2 and FH when compared to samples with Aβ only. On the contrary, apoE2 and FH increased TLR6 expression levels while apoE4 increased complement factor D and TREM2 expression levels. These results are now included in the manuscript Fig 5B panel and in lines 285-289

2. To increase the readability of their figures, they may want to consider including colored denotations of the ligands that were stained as opposed to highlighting the fluorochromes. For instance, DAPI written in blue, FH written in green, etc.

Thank you. Adding colored denotations in the microscopy images is a good idea. These are now included in the revised manuscript

3. In the figure legends (i.e. Figure 1A-B, 3A-B) it would be helpful if they briefly indicated what regions of the brains were stained in these studies.

The samples are from the right frontal cortex. This is now indicated clearly in the figure legends.

4. In regards to the statement that spans lines 89-91, it would be interesting to know whether any of the reported mutations or polymorphisms in FH have been linked to Alzheimer's disease or any other neurological disorders.

Yes. There are conflicting results in two genetics studies on association of Y402H polymorphism in AD. This possible link is important to point out and is now mentioned in the manuscript lines 81-84 with appropriate references:

1. *Zetterberg M, Landgren S, Andersson ME, Palmer MS, Gustafson DR, Skoog I, et al. Association of complement factor H Y402H gene polymorphism with Alzheimer's disease. Am J Med Genet B Neuropsychiatr Genet (2008) 147B:720–6. doi: 10.1002/ajmg.b.30668*
2. *Le Fur I, Laumet G, Richard F, Fievet N, Berr C, Rouaud O, et al. Neurobiol Aging (2010) 31:165–6. doi: 10.1016/j.neurobiolaging.2008.03.003*

5. In their human immunofluorescence studies, they conclude that all Iba-1-expressing cells are microglia. Given that both microglia and peripheral-derived macrophages can express Iba-1 in the inflamed brain, they should consider clarifying this statement or bolstering their studies with the analysis of other microglia-specific markers.

This is true that Iba-1 expression does not necessary only stain microglia as peripheral macrophages can infiltrate the brain at certain stages of AD. We have now toned down this statement in the manuscript by describing peripheral macrophages in lines 174-176 and by replacing microglia with phagocytic cells in line 163 and the synopsis Figure.

6. In the first sentence of the second paragraph describing the data presented in Figure 3, they casually make a biological link between CR3 and CD11b. Before discussing the data interpretation in regards to CD11b (Figure 3F and G), it would be better to provide additional background information on the subunit composition of CR3, especially for those outside of the complement field.

We agree with the reviewer that additional information on the subunit composition of CR3 is needed. This has now been explained in lines 184-188 in the revised manuscript before describing the results in Figure 3 including a reference:
Lamers C, Pluss CJ, Ricklin D (2021) The Promiscuous Profile of Complement Receptor 3 in Ligand Binding, Immune Modulation, and Pathophysiology. Front Immunol 12: 662164

7. In relation to the data presented in Figure 4G, they conclude that their findings demonstrating reduced Aβ1-42 phagocytosis point towards a prevention of plaque growth. However, they have not rigorously tested this in their model and it would be better to tone down this conclusion to limit data over-interpretation. Conversely, if they feel as though this is an important facet of their story, they should then conduct more extensive and targeted studies to directly assess this.

It is true that the role of increased microglial Aβ1-42 uptake and increased plaque growth is not addressed by the experiments of this manuscript and this statement is based on earlier studies:

Baik SH, Kang S, Son SM, Mook-Jung I (2016) Microglia contributes to plaque growth by cell death due to uptake of amyloid beta in the brain of Alzheimer's disease mouse model. Glia 64: 2274-2290

Huang Y, Happonen KE, Burrola PG, O'Connor C, Hah N, Huang L, Nimmerjahn A, Lemke G (2021b) Microglia use TAM receptors to detect and engulf amyloid beta plaques. Nat Immunol 22: 586-594

This conclusion has now been toned down in the revised manuscript and the text in line 257 has been changed by the removing sentence "implying that reduced phagocytosis could, as shown by others, prevent plaque growth and reduce inflammatory and neurotoxic signals mediated by microglial cells that may lead to defective Aβ clearance (Baik et al., 2016)", and lines 365 "On the contrary, current observations suggest that microglial phagocytosis rather promotes than inhibits plaque development", and 367 "Our study also supports a minor and even a harmful role of cellular uptake in Aβ clearance". We also changed the meaning of sentence in Line 370: "Therefore, inhibition of uptake of Aβ > controlled uptake of Aβ".

8. The transcriptomics data presented in Figure 5 could be quite informative; however, they have only provided a preliminary, surface-level assessment especially of the differences between the two most relevant groups (i.e. Aβ+FH+ApoE2 vs. Aβ+FH+ApoE4). Deeper analysis could be illuminating and provide additional insights into potential neuroinflammatory differences between the groups (see point number one above). At the very least they should consider performing pathway analysis (KEGG, GO, etc.) and providing a volcano plot highlighting major differentially expressed genes between the Aβ+FH+apoE2 and Aβ+FH+apoE4 experimental groups.

We have done additional analysis of the mRNA data focusing on traditional neuroinflammatory markers (see answer 1). In Fig. 5C we have added a volcano plot pointing out those genes that showed differential expression between Aβ+FH+apoE2 and Aβ+FH+apoE4.

In addition, we have performed pathway analysis of these genes (see Fig. below) from which we have selected the most relevant pathways to be included in the revised manuscript as Fig. 5E.

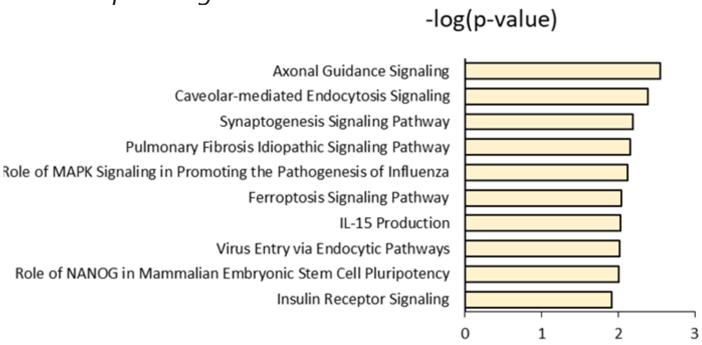

9. The 2016 Tarasoff-Conway et al. manuscript that they cite in the Results and Discussion sections to support their assertion that the majority of extracellular Aβ is removed across the BBB is a review article and it would be better to include citations from the primary literature that supports this statement.

We agree that original articles should be mentioned here. We replaced the review article with two original articles showing importance of BBB in Aβ removal in lines 245, 344 and 365 in the revised manuscript:

1. *Zhao Z, Sagare AP, Ma Q, Halliday MR, Kong P, Kisler K, Winkler EA, Ramanathan A, Kanekiyo T, Bu G et al (2015) Central role for PICALM in amyloid-beta blood-brain barrier transcytosis and clearance. Nat Neurosci 18: 978-987*
2. *Iliff JJ, Wang M, Liao Y, Plogg BA, Peng W, Gundersen GA, Benveniste H, Vates GE, Deane R, Goldman SA et al (2012) A paravascular pathway facilitates CSF flow through the brain parenchyma and the clearance of interstitial solutes, including amyloid beta. Sci Transl Med 4: 147ra111*

Referee #2:

In this manuscript, Chernyaeva and colleagues investigate the relationship of APOE with complement factor H in the context of amyloid beta. The authors focus on idiopathic hydrocephalus, an aging-associated neurodegenerative disease that is characterized by amyloid beta deposition. The authors wanted to investigate whether interactions between APOE and Factor H could regulate the interactions between Abeta and APOE and thus reduce the neurotoxic effects of Abeta. They show that Factor H binds to APOE in an isoform-dependent manner (E2>E3>E4). Binding of Factor H to APOE reduces Abeta42 aggregation, toxicity and its effect on immune activation. The results are new and inform on the importance of complement-APOE interaction in the context of Abeta.
A general comment - the authors seem to weave in and out of iNPH and AD especially in the beginning of the Results section. It would be great if the authors could focus on one line of thought in the Results section. This associative inference may be more appropriately placed in the Discussion section. In addition, it would be appropriate not to refer to presence of Abeta as AD especially in patients (in the absence of tau pathology or dementia).

> *We have now removed the first sentence before line 99: "Although apoE isoforms differ by only two amino acid residues at positions 112 and 158 the observed AD association and the location of these residues indicate functional and structural differences between these proteins (Frieden & Garai, 2012). The age-related macular degeneration (AMD) risk variant 402H of FH (FH402H) been suggested to associate with AD (Zetterberg et al., 2008).". Also the first sentence in introduction has been removed from line 43 to 56-58 to keep focus on the iNPH patient samples. AD is presented in the transcriptomic and proteomic data, as the genes of this disease have been extensively studied in the context of Aβ clearance, and is presented in the searched databases.* To avoid referring to the presence of Aβ in patients we have removed sentence "as markers of early AD pathology in vivo" in line 92.

Some comments on the data:
(1) Fig1A is a little confusing - the key placed inside the panel depicts all ligands in white pseudocolor but the panels have fluors of different colors. Importantly, no APOE (red) is visible on amyloid deposits (white) - this seem to be somewhat confusing as well. Also, CFH is predominantly expressed from endothelial cells - could authors surmise how this is leaked out into the brain parenchyma? In panel 1D, statistics has been done on n=2 - is this statistically acceptable?

> *We thank for a thorough review. Adding colored denotations in the microscopy images is a good idea. These are now included in the revised manuscript*

It is true that apoE is found on Aβ plaques and that is what we also observed in the biopsy samples with Aβ. We have now added representative microscopy images in Fig. 1C from biopsy sample taken from a patient that has also been diagnosed for Alzheimer's clinical syndrome (ACS) (based on the next suggestion by the reviewer). Colocalization of Aβ and apoE is shown with an arrow. However, there were also Aβ without apoE. This is not clear in the current version of the manuscript and we have pointed this out in line 106-107 in the revised manuscript in addition to showing representative images in Fig. 1C and all images in Appendix Fig. S2.

It is also true that FH is found on endothelial cells and perivascular space and therefore some of detected FH can be from blood due to leakage of BBB as BBB is compromised in AD. Colocalization of apoE and FH in non-perivascular space is also shown in EV1 A and B. The role of BBB leakage in AD is now discussed in the revised manuscript in lines 327-329.

The statistics has been removed from panel 1D

An additional piece of data would be useful as authors mention (line 98-100) that iNPH patients show colocalization .... as markers of early AD pathology in vivo'. In lines 112-113, they additionally mention that iNPH is a prodromal form of AD. In light of this, it will be good to compare APOE/FH/Abeta colocalization in AD brains vs iNPH brains.

It is true that in iNPH a number of patients have comorbid prodromal AD. We decided not to analyze post mortem AD biopsy samples as they would not be comparable to the biopsy samples taken from living humans. Also, comparing apoE23 and apoE44 within AD patients is impossible with the patient samples collected, as AD patients carry apoE23 genotype very rarely. To be able to analyze colocalization in AD brains and compare to the biopsy samples we have now included two iNPH cases that have both Aβ and Tau pathology that indicate a definitive neuropathological diagnosis. From these two patients one has also been diagnosed for Alzheimer's clinical syndrome (ACS) and has the risk allele. We have performed colocalization analysis of the frontal cortex biopsy sample of this patient and included the data in Fig 1C and Fig1D in apoE44 dataset. This sample is also included in the proteomic data as sample apoE44.

(2) In Fig 2, please remove the cartoons - these are distracting and did not help.

The cartoons have been removed to clarify the data

No APOE4 (negative) or APOE3 data is provided for panels C-E. This is important as APOE3 is the reference allele and APOE2 the protective allele as far as Abeta is concerned
Another comment is that since in Fig 1, the authors show that APOE4 does not bind to FH, would it not imply that experiments testing FH binding to APOE4-Abeta will not change anything (Fig 2)?

We have used maleimide labeling chemistry that lables cysteine in the protein to be able to perform single molecule fluorescence imaging and photobleaching experiments. Changing the chemistry to a non-specific multi-site fluorescent label is not optimal for high resolution single-molecule imaging. Because apoE4 does not contain a free cysteine and cannot be labeled using maleimide chemistry we chose to perform the experiment with the reference isoform apoE3. These data are included in Fig. 2D and E in the revised

manuscript showing that "Reduction in the number of fluorophores/foci that measures the stoichiometry of the apoE/Aβ1-42 complex was more striking on apoE2/Aβ1-42 complexes than apoE3/Aβ1-42 complexes in the presence of FH." as described in lines 153-155. This lines very well with our finding where FH binds especially to apoE2 in apoE2/Aβ1-42 complex and therefore reduces apoE/Aβ1-42 oligomerization.

We agree that apoE4 will not change anything and this is shown in Fig. 2B where apoE4 protein is similar in the presence and absence of FH while less oligomerization of apoE2 can be seen in the presence than in absence of FH.

(3) In Fig 3, confocal microscopy or other type of analysis need to be shown to establish colocalization of FH with microglia and Abeta plaques.

We apologize for unclear conclusions. We did not measure colocalization between FH, microglia and Aβ but observed that FH can be found in the vicinity of microglia and Aβ plaques. We have been more clear with this in line 176 in the revised manuscript.

Most importantly, there is no change in FH intensity between the two genotypes (panel E), which contradicts the premise of the paper (APOE isoform dependent binding to FH).

It is true that the quantity of FH did not differ between the samples. We only observed significantly increased colocalization between apoE33/23 when compared to apoE44/34 (Fig. 1). This is consistent with our in vitro data (Fig. 1) showing that FH binds apoE2/3 but not apoE4. We have clarified this in lines 178-179 in the revised manuscript.

Also, could the authors explain using U937 cells instead of primary microglia?

Using primary microglia is indeed a biologically more relevant option than cell lines. The motivation to use CR3 overexpressed cells vs empty cells was to be able to study the role of complement activation, iC3b formation by FH and interaction with CR3. Because the main focus of our study was on apoE we could also exclude the effect of different apoE genotypes in the experiment as U937 cells are genetically uniform. This has been explained more thoroughly in lines 184-185 in the revised manuscript.

No data with the reference isoform (APOE3) was provided in Fig. 3D-G.

We thank the reviewer of this important point. We have now performed apoE3 experiments for Figs3 F-H. Binding of 405-apoE3 is now shown in Fig. 3H and Aβ binding to CR3 in the presence of apoE3 and apoE3+FH in Fig. 3I right. In addition, we have corrected a typo in Fig. 3H > activated as + sign is missing from activated CR3.

(4) Usage of the AMD associated mutant is informative but is this relevant to abeta oligomerization?

We did not study the role of FH402H (AMD mutant) in Aβ oligomerization in this study as both the wild type FH402Y and AMD associated FH402H both bound apoE2 equally well. Based on the structural MS crosslinking data and molecular dynamics analysis, the amino acid 402 is not located near the apoE-FH interaction site (See Fig.

below where residue 402 is highlighted in red in the complex model). This question is, however, relevant and we have shortly discussed this in lines 347-350 in the revised manuscript and added a Fig. EV2B of the structure.

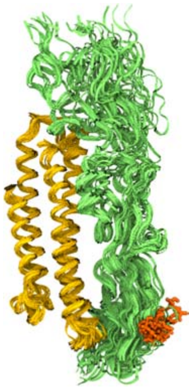

(5) In Fig 5, TREM2 does not seem to be upregulated in the data - generally Trem2 or sTrem2 levels track with Abeta. Can the authors comment?

We thank reviewer for pointing this out and we have now analyzed TREM2 mRNA expression levels in the revised manuscript. Interestingly, we can detect increased TREM2 expression in the presence of apoE4. Here, the presence of FH did not affect TREM2 expression levels. This data is now included in Fig. 5B and discussed in lines 285-289 in the revised manuscript. TREM2 expression could not be detected in sufficient levels in the proteomic data.

(6) Fig. 6 is confusing. Has the proteomics been done on healthy individuals or iNPH individuals? If these are not from iNPH individuals or AD individuals, then is it fair to summarize these results as written in lines 305-307?

We apologize for unclearness. The proteomics have been done from iNPH biopsy samples. This has been clarified in the revised manuscript in lines 297 and 681.

1st Revision - Editorial Decision 6th Apr 2023

Dear Dr. Haapasalo

Thank you for the submission of your revised manuscript to EMBO reports. We have now received the full set of referee reports that is copied below.

As you will see, both referees are very positive about the study and support publication.

Browsing through the manuscript myself, I noticed a few editorial things that we need before we can proceed with the official acceptance of your study.

- Please reduce the number of keywords to 5.

- Please update the 'Conflict of interest' paragraph to our new 'Disclosure and competing interests statement'. For more information see
https://www.embopress.org/page/journal/14693178/authorguide#conflictsofinterest

- Regarding the Author Contributions, we now use CRediT to specify the contributions of each author in the journal submission system. CRediT replaces the author contribution section, which therefore needs to be removed from the manuscript text. You can use the free text box in our system if you wish to provide more detailed descriptions. See also guide to authors https://www.embopress.org/page/journal/14693178/authorguide#authorshipguidelines.

- Please note that all data described and mentioned need to be shown in the manuscript. In this respect we notice a statement on 'data not shown' on page 10.

- Funding information: The Academy of Finland grant number is 1331108 in our online submission system but 331108 in the manuscript. Please check and correct.

- Please add callouts to Fig 3G, EV2A&B, and EV4D.

- Datasets: Please add the name e.g., 'Dataset EV1' to the sheet. Please remove the dataset legends from the manuscript file and add it to a tab in the .xls file.

- Appendix: Please add page numbers to the table of content.

- Appendix Fig. S1: please define the size of the scale bar in the legend, not in the image itself. The text in the blue channel is hardly visible, please choose a different shade of blue. Text size could also be increased.

- Appendix Fig. S2: please add a scale bar and define its size in the legend. The text in the individual images should be in larger font. It is hardly visible, in particular in the blue channel.

- Please remove the movie legends from the manuscript file and supply them as README.txt files instead. Then ZIP each movie with its legend and upload the ZIP files.

- It appears that the source data for Fig 2E is missing. Can you please provide it?

- I attach to this email a related manuscript file with comments by our data editors. Please address all comments and upload a revised file with tracked changes with your final manuscript submission.

- Finally, the synopsis image is accompanied online by A) a short (1-2 sentences) summary of the findings and their significance, and B) 2-3 bullet points highlighting key results. Please send us this information along with the revised manuscript.
In the synopsis image you show 'apoE binding' at the top. Would 'affinity for factor H' not be more intuitive? Because then the image shows the apoE isoforms and the difference between them is their binding to FH.

We look forward to seeing a final version of your manuscript as soon as possible.

With kind regards,

Martina Rembold, PhD
Senior Editor
EMBO reports

****

Referee #1:

The authors have done an outstanding job of addressing my comments.

Referee #2:

The authors have responded to all critiques satisfactorily.

The authors have addressed all minor editorial requests.

2nd Revision - Editorial Decision 24th Apr 2023

Dr. Karita Haapasalo
University of Helsinki
Finland

Dear Dr. Haapasalo,

I am very pleased to accept your manuscript for publication in the next available issue of EMBO reports. Thank you for your contribution to our journal.

At the end of this email I include important information about how to proceed. Please ensure that you take the time to read the information and complete and return the necessary forms to allow us to publish your manuscript as quickly as possible.

As part of the EMBO publication's Transparent Editorial Process, EMBO reports publishes online a Review Process File to accompany accepted manuscripts. As you are aware, this File will be published in conjunction with your paper and will include the referee reports, your point-by-point response and all pertinent correspondence relating to the manuscript.

If you do NOT want this File to be published, please inform the editorial office within 2 days, if you have not done so already, otherwise the File will be published by default [contact: emboreports@embo.org]. If you do opt out, the Review Process File link will point to the following statement: "No Review Process File is available with this article, as the authors have chosen not to make the review process public in this case."

Thank you again for your contribution to EMBO reports and congratulations on a successful publication. Please consider us again in the future for your most exciting work.

Yours sincerely,

Martina Rembold, PhD
Senior Editor
EMBO reports

****

THINGS TO DO NOW:

Please note that you will be contacted by Wiley Author Services to complete licensing and payment information. The required 'Page Charges Authorization Form' is available here: https://www.embopress.org/pb-assets/embo-site/er_apc.pdf - please download and complete the form and return to embopressproduction@wiley.com

EMBO Press participates in many Publish and Read agreements that allow authors to publish Open Access with reduced/no publication charges. Check your eligibility: https://authorservices.wiley.com/author-resources/Journal-Authors/open-access/affiliation-policies-payments/index.html

You will receive proofs by e-mail approximately 2-3 weeks after all relevant files have been sent to our Production Office; you should return your corrections within 2 days of receiving the proofs.

Please inform us if there is likely to be any difficulty in reaching you at the above address at that time. Failure to meet our deadlines may result in a delay of publication, or publication without your corrections.

All further communications concerning your paper should quote reference number EMBOR-2022-56467V3 and be addressed to emboreports@wiley.com.

Should you be planning a Press Release on your article, please get in contact with emboreports@wiley.com as early as possible, in order to coordinate publication and release dates.

EMBO Press Author Checklist

Corresponding Author Name: Karita Haapasalo	
Journal Submitted to: EMBO Reports	
Manuscript Number: EMBOR-2022-56467V2	

USEFUL LINKS FOR COMPLETING THIS FORM
The EMBO Journal - Author Guidelines
EMBO Reports - Author Guidelines
Molecular Systems Biology - Author Guidelines
EMBO Molecular Medicine - Author Guidelines

Reporting Checklist for Life Science Articles (updated January 2022)
This checklist is adapted from Materials Design Analysis Reporting (MDAR) Checklist for Authors. MDAR establishes a minimum set of requirements in transparent reporting in the life sciences (see Statement of Task: 10.31222/osf.io/9sm4x). Please follow the journal's guidelines in preparing your manuscript.
Please note that a copy of this checklist will be published alongside your article.

Abridged guidelines for figures
1. Data
The data shown in figures should satisfy the following conditions:
- the data were obtained and processed according to the field's best practice and are presented to reflect the results of the experiments in an accurate and unbiased manner.
- ideally, figure panels should include only measurements that are directly comparable to each other and obtained with the same assay.
- plots include clearly labeled error bars for independent experiments and sample sizes. Unless justified, error bars should not be shown for technical replicates.
- if n<5, the individual data points from each experiment should be plotted. Any statistical test employed should be justified.
- Source Data should be included to report the data underlying figures according to the guidelines set out in the authorship guidelines on Data Presentation.

2. Captions
Each figure caption should contain the following information, for each panel where they are relevant:
- a specification of the experimental system investigated (eg cell line, species name).
- the assay(s) and method(s) used to carry out the reported observations and measurements.
- an explicit mention of the biological and chemical entity(ies) that are being measured.
- an explicit mention of the biological and chemical entity(ies) that are altered/varied/perturbed in a controlled manner.
- the exact sample size (n) for each experimental group/condition, given as a number, not a range;
- a description of the sample collection allowing the reader to understand whether the samples represent technical or biological replicates (including how many animals, litters, cultures, etc.).
- a statement of how many times the experiment shown was independently replicated in the laboratory.
- definitions of statistical methods and measures:
  - common tests, such as t-test (please specify whether paired vs. unpaired), simple χ2 tests, Wilcoxon and Mann-Whitney tests, can be unambiguously identified by name only, but more complex techniques should be described in the methods section;
  - are tests one-sided or two-sided?
  - are there adjustments for multiple comparisons?
  - exact statistical test results, e.g., P values = x but not P values < x;
  - definition of 'center values' as median or average;
  - definition of error bars as s.d. or s.e.m.

Please complete ALL of the questions below.
Select "Not Applicable" only when the requested information is not relevant for your study.

Materials

Newly Created Materials	Information included in the manuscript?	In which section is the information available? (Reagents and Tools Table, Materials and Methods, Figures, Data Availability Section)
New materials and reagents need to be available; do any restrictions apply?	Not Applicable	

Antibodies	Information included in the manuscript?	In which section is the information available? (Reagents and Tools Table, Materials and Methods, Figures, Data Availability Section)
For **antibodies** provide the following information:<br>- Commercial antibodies: RRID (if possible) or supplier name, catalogue number and or/clone number<br>- Non-commercial: RRID or citation	Yes	Materials and Methods

DNA and RNA sequences	Information included in the manuscript?	In which section is the information available? (Reagents and Tools Table, Materials and Methods, Figures, Data Availability Section)
**Short novel DNA or RNA including primers, probes**: provide the sequences.	Not Applicable	

Cell materials	Information included in the manuscript?	In which section is the information available? (Reagents and Tools Table, Materials and Methods, Figures, Data Availability Section)
**Cell lines:** Provide species information, strain. Provide accession number in repository **OR** supplier name, catalog number, clone number, and/**OR** RRID.	Yes	Materials and Methods
**Primary cultures:** Provide species, strain, sex of origin, genetic modification status.	Not Applicable	
Report if the cell lines were recently **authenticated** (e.g., by STR profiling) and tested for mycoplasma contamination.	Yes	Materials and Methods

Experimental animals	Information included in the manuscript?	In which section is the information available? (Reagents and Tools Table, Materials and Methods, Figures, Data Availability Section)
**Laboratory animals or Model organisms:** Provide species, strain, sex, age, genetic modification status. Provide accession number in repository **OR** supplier name, catalog number, clone number, **OR** RRID.	Not Applicable	
**Animal observed in or captured from the field:** Provide species, sex, and age where possible.	Not Applicable	
Please detail **housing and husbandry conditions**.	Not Applicable	

Plants and microbes	Information included in the manuscript?	In which section is the information available? (Reagents and Tools Table, Materials and Methods, Figures, Data Availability Section)
**Plants:** provide species and strain, ecotype and cultivar where relevant, unique accession number if available, and source (including location for collected wild specimens).	Not Applicable	
**Microbes:** provide species and strain, unique accession number if available, and source.	Yes	Materials and methods

Human research participants	Information included in the manuscript?	In which section is the information available? (Reagents and Tools Table, Materials and Methods, Figures, Data Availability Section)
If collected and within the bounds of privacy constraints report on age, sex and gender or ethnicity for all study participants.	Yes	Appendix Table S1

Core facilities	Information included in the manuscript?	In which section is the information available? (Reagents and Tools Table, Materials and Methods, Figures, Data Availability Section)
If your work benefited from core facilities, was their service mentioned in the acknowledgments section?	Not Applicable	

Design

Study protocol	Information included in the manuscript?	In which section is the information available? (Reagents and Tools Table, Materials and Methods, Figures, Data Availability Section)
If study protocol has been **pre-registered, provide DOI in the manuscript**. For clinical trials, provide the trial registration number **OR** cite DOI.	Not Applicable	
Report the **clinical trial registration number** (at ClinicalTrials.gov or equivalent), where applicable.	Not Applicable	

Laboratory protocol	Information included in the manuscript?	In which section is the information available? (Reagents and Tools Table, Materials and Methods, Figures, Data Availability Section)
Provide DOI OR other citation details if **external detailed step-by-step protocols** are available.	Not Applicable	

Experimental study design and statistics	Information included in the manuscript?	In which section is the information available? (Reagents and Tools Table, Materials and Methods, Figures, Data Availability Section)
Include a statement about **sample size** estimate even if no statistical methods were used.	Yes	Figure legends and Materials and methods
Were any steps taken to minimize the effects of subjective bias when allocating animals/samples to treatment (e.g. **randomization procedure**)? If yes, have they been described?	Not Applicable	
Include a statement about **blinding** even if no blinding was done.	Not Applicable	
Describe **inclusion/exclusion criteria** if samples or animals were excluded from the analysis. Were the criteria pre-established?<br><br>If sample or data points were omitted from analysis, report if this was due to attrition or intentional exclusion and provide justification.	Not Applicable	
For every figure, are **statistical tests** justified as appropriate? Do the data meet the assumptions of the tests (e.g., normal distribution)? Describe any methods used to assess it. Is there an estimate of variation within each group of data? Is the variance similar between the groups that are being statistically compared?	Yes	Materials and Methods

Sample definition and in-laboratory replication	Information included in the manuscript?	In which section is the information available? (Reagents and Tools Table, Materials and Methods, Figures, Data Availability Section)
In the figure legends: state number of times the experiment was **replicated** in laboratory.	Yes	Figure legends
In the figure legends: define whether data describe **technical or biological replicates**.	Yes	Figure legends

Ethics

Ethics	Information included in the manuscript?	In which section is the information available? (Reagents and Tools Table, Materials and Methods, Figures, Data Availability Section)
Studies involving **human participants**: State details of **authority granting ethics approval** (IRB or equivalent committee(s), provide reference number for approval.	Yes	Materials and Methods
Studies involving **human participants**: Include a statement confirming that **informed consent** was obtained from all subjects and that the experiments conformed to the principles set out in the WMA Declaration of Helsinki and the Department of Health and Human Services Belmont Report.	Yes	Materials and Methods
Studies involving **human participants:** For publication of **patient photos**, include a statement confirming that consent to publish was obtained.	Not Applicable	
Studies involving experimental **animals**: State details of **authority granting ethics approva**l (IRB or equivalent committee(s), provide reference number for approval. Include a statement of compliance with ethical regulations.	Not Applicable	
Studies involving **specimen and field samples:** State if relevant **permits** obtained, provide details of authority approving study; if none were required, explain why.	Not Applicable	

Dual Use Research of Concern (DURC)	Information included in the manuscript?	In which section is the information available? (Reagents and Tools Table, Materials and Methods, Figures, Data Availability Section)
Could your study fall under dual use research restrictions? Please check biosecurity documents and list of **select agents and toxins** (CDC): https://www.selectagents.gov/sat/list.htm	Not Applicable	
If you used a select agent, is the security level of the lab appropriate and reported in the manuscript?	Not Applicable	
If a study is subject to dual use research of concern regulations, is the name of the **authority granting approval and reference number** for the regulatory approval provided in the manuscript?	Not Applicable	

Reporting

The MDAR framework recommends adoption of discipline-specific guidelines, established and endorsed through community initiatives. Journals have their own policy about requiring specific guidelines and recommendations to complement MDAR.

Adherence to community standards	Information included in the manuscript?	In which section is the information available? (Reagents and Tools Table, Materials and Methods, Figures, Data Availability Section)
State if relevant guidelines or checklists (e.g., **ICMJE, MIBBI, ARRIVE, PRISMA**) have been followed or provided.	Not Applicable	
For **tumor marker prognostic studies,** we recommend that you follow the **REMARK** reporting guidelines (see link list at top right). See author guidelines, under 'Reporting Guidelines'. Please confirm you have followed these guidelines.	Not Applicable	
For **phase II and III randomized controlled trials**, please refer to the **CONSORT** flow diagram (see link list at top right) and submit the CONSORT checklist (see link list at top right) with your submission. See author guidelines, under 'Reporting Guidelines'. Please confirm you have submitted this list.	Not Applicable	

Data Availability

Data availability	Information included in the manuscript?	In which section is the information available? (Reagents and Tools Table, Materials and Methods, Figures, Data Availability Section)
Have **primary datasets** been deposited according to the journal's guidelines (see 'Data Deposition' section) and the respective accession numbers provided in the Data Availability Section?	Yes	Data Availability Section
Were **human clinical and genomic datasets** deposited in a public access-controlled repository in accordance to ethical obligations to the patients and to the applicable consent agreement?	Yes	Data Availability Section
Are **computational models** that are central and integral to a study available without restrictions in a machine-readable form? Were the relevant accession numbers or links provided?	Not Applicable	
If publicly available data were reused, provide the respective **data citations in the reference list**.	Not Applicable	
